# Randomised double-blind placebo-controlled trial protocol to evaluate the therapeutic efficacy of lyophilised faecal microbiota capsules amended with next-generation beneficial bacteria in individuals with metabolic dysfunction-associated steatohepatitis

**DOI:** 10.1136/bmjopen-2024-088290

**Published:** 2025-01-09

**Authors:** Quinten J. J. Augustijn, Aldo Grefhorst, Pleun de Groen, Koen Wortelboer, Jos F ML Seegers, Ismail Sahin Gül, Peter Suenaert, Joanne Verheij, Willem M. de Vos, Hilde Herrema, Max Nieuwdorp, Adriaan G. Holleboom

**Affiliations:** 1 Department of Experimental Vascular Medicine, Amsterdam University Medical Centres, Amsterdam, Netherlands; 2 University of Amsterdam, Amsterdam, Netherlands; 3 Caelus Health, Heiloo, Netherlands; 4 Akkermansia Company, Mont-Saint-Guibert, Belgium; 5 Pathology, AMC, Amsterdam, Netherlands; 6 Wageningen University, Wageningen, Netherlands; 7 Amsterdam University Medical Centres, Amsterdam, Netherlands; 8 Department of Internal Medicine, Amsterdam UMC, Vrije Universiteit Amsterdam, Academisch Medisch Centrum, Amsterdam, Netherlands; 9 Amsterdam UMC Locatie AMC, Amsterdam, Netherlands

**Keywords:** Hepatology, Vascular medicine, GASTROENTEROLOGY

## Abstract

**Background:**

The spectrum of metabolic dysfunction-associated steatotic liver disease (MASLD) is highly prevalent, affecting 30% of the world’s population, with a significant risk of hepatic and cardiometabolic complications. Different stages of MASLD are accompanied by distinct gut microbial profiles, and several microbial components have been implicated in MASLD pathophysiology. Indeed, earlier studies demonstrated that hepatic necroinflammation was reduced in individuals with MASLD after allogenic faecal microbiota transplantation (FMT) from healthy donors on a vegan diet. Here, we further investigate the therapeutic potential of gut microbiome modulation using a syntrophic combination of next-generation beneficial bacteria with FMT in individuals with advanced MASLD.

**Methods and analysis:**

This trial is a randomised, double-blind, placebo-controlled study investigating the therapeutic potential of lyophilised faecal microbiota capsules (LFMCs) in individuals with metabolic dysfunction-associated steatohepatitis. In this study, 48 participants will be randomised 1:1 to receive either healthy vegan donor LFMCs or placebo for 24 weeks. In addition, all participants will be supplemented with a set of next-generation beneficial bacteria, including *Anaerobutyricum soehngenii*, pasteurised *Akkermansia muciniphila* and *Bifidobacterium animalis* subsp. *lactis*, as well as fructo-oligosaccharides. A liver biopsy will be performed at baseline and at the end of the trial. In addition, participants will be assessed through MRI, FibroScan, blood tests, faecal samples and continuous glucose monitoring. The first participant was enrolled on 25 April 2023.

**Ethics and dissemination:**

Ethical approval was obtained from the Medical Ethics Committee of the University Medical Centre of Amsterdam. The results of this study will be disseminated through peer-reviewed journals.

**Trial Registration number:**

The trial is registered on clinicaltrials.gov (NCT05821010).

**STRENGTHS AND LIMITATIONS OF THIS STUDY**

- Participants receive continuous faecal microbiota transplantation therapy via capsules.
- In addition to liver histology and RNAseq data in liver biopsies, this study uses multiparametric MRI, transient elastography and enhanced liver fibrosis (ELF) panel as secondary outcome measures.
- The study does not have an arm in which participants receive no treatment.
- This is a single-centre study.

## Introduction

 The spectrum of metabolic dysfunction-associated steatotic liver disease (MASLD) is highly prevalent, affecting 30% of the global population.^1^ The prevalence has increased by an alarming 50% over the past two decades, paralleling the rise in obesity and type 2 diabetes.^1^ Approximately 7–29% of individuals with MASLD have metabolic dysfunction-associated steatohepatitis (MASH), an advanced stage marked by lobular inflammation and characteristic liver cell injury known as 'ballooning’.^2^ Subsequently, MASH can induce hepatic fibrosis, which may ultimately lead to cirrhosis and hepatocellular carcinoma. Fibrotic MASH is independently associated with atherosclerotic cardiovascular disease, chronic kidney disease and liver-related and overall mortality.^3^,^9^

Interestingly, the gut microbiome and MASLD are strongly correlated.^10^,^12^ Compared with healthy controls, individuals with MASLD have altered gut microbial signatures, and these alterations correlate with incremental stages of MASLD. Typically, individuals with MASLD have reduced microbial diversity and increased abundances of *Escherichia*, *Prevotella* and *Streptococcus*, while genera associated with health benefits such as *Coprococcus*, *Faecalibacterium* and *Ruminococcus* are decreased.^10 13^ Moreover, more severe fibrotic stages exhibited an increased abundance of Gram-negative bacteria *Escherichia coli* and *Bacteroides vulgatus*.^14^ Some studies report the effect of gut microbial modulation on MASLD and its associated cardiometabolic diseases. For instance, the use of pre- and probiotics to modulate the gut microbiome may have beneficial effects as reflected by changes in MASLD-related biomarkers, yet the sample size and endpoint readouts of these trials thus far were limited.^15 16^

Although several studies show the beneficial effects of lean donor faecal microbiota transplantation (FMT) on cardiometabolic parameters in individuals with metabolic syndrome, to date only three studies have specifically investigated the therapeutic potential of FMT in individuals with MASLD.^17^,^19^ First, Xue *et al* compared colonic-administered FMT with oral probiotics in a non-randomised open-label trial with a 1 month follow-up.^20^ Although this study found a decrease in liver fat attenuation scores assessed by FibroScan after FMT treatment, the implications are difficult to interpret due to the study design, outcome measures, short follow-up and incomplete reporting of potential confounders. Second, a Canadian randomised placebo-controlled trial in 21 individuals comparing a single dose of allogenic with autologous FMT via nasoduodenal tube found an improvement in intestinal barrier function, but no improvement in liver fat content as assessed by MRI proton density fat fraction (MRI-PDFF).^21^ However, this study is also difficult to interpret, mainly due to its single-dose nature, short 6 week follow-up and small sample size. Thirdly, a randomised controlled trial from our group compared three infusions of allogenic versus autologous FMT via nasoduodenal tube administered every 8 weeks.^22^ Although this study also had a small sample size, an allogenic FMT from lean donors on a vegan diet reduced the histopathological hepatic necroinflammation score in individuals with MASLD. Participants treated with the allogenic FMT displayed an increased relative abundance of butyrate-producing bacteria such as *Faecalibacterium prausnitzii* and *Anaerobutyricum soehngenii* (previously termed *Eubacterium hallii*^23^). Unfortunately, the latter study did not use validated imaging outcome measures to confirm histopathological scores and did not identify a clear causal mechanistic explanation.

The multifaceted interaction between the gut microbiota, intestine and the liver is commonly referred to as the ‘gut-liver axis’.^24^ One of the mechanisms involved is the production of gut microbial metabolites. Beneficial metabolites include short-chain fatty acids (SCFAs) such as butyrate, propionate and acetate. These metabolites may increase insulin sensitivity and reduce inflammation, pathways central in MASH pathogenesis.^25^ In contrast, the production of harmful metabolites such as ethanol might spur MASLD and MASH progression.^26^ A second mechanism linking gut microbes to MASLD is gut barrier function.^24 27^ Although difficult to study in humans, animal studies show that an impaired gut barrier function leads to increased uptake of pro-inflammatory agents, such as lipopolysaccharides (LPS), which drain directly into the liver via the portal vein, causing low-grade inflammation.^2527^,^30^ The gut microbiome strongly influences gut barrier function.^27^ Various gut microbes and gut-derived metabolites have been reported to hamper barrier function, while others, such as *A. soehngenii* and *Akkermansia muciniphila*, exert a beneficial effect on the gut barrier function.^31^

**Figure 1:**
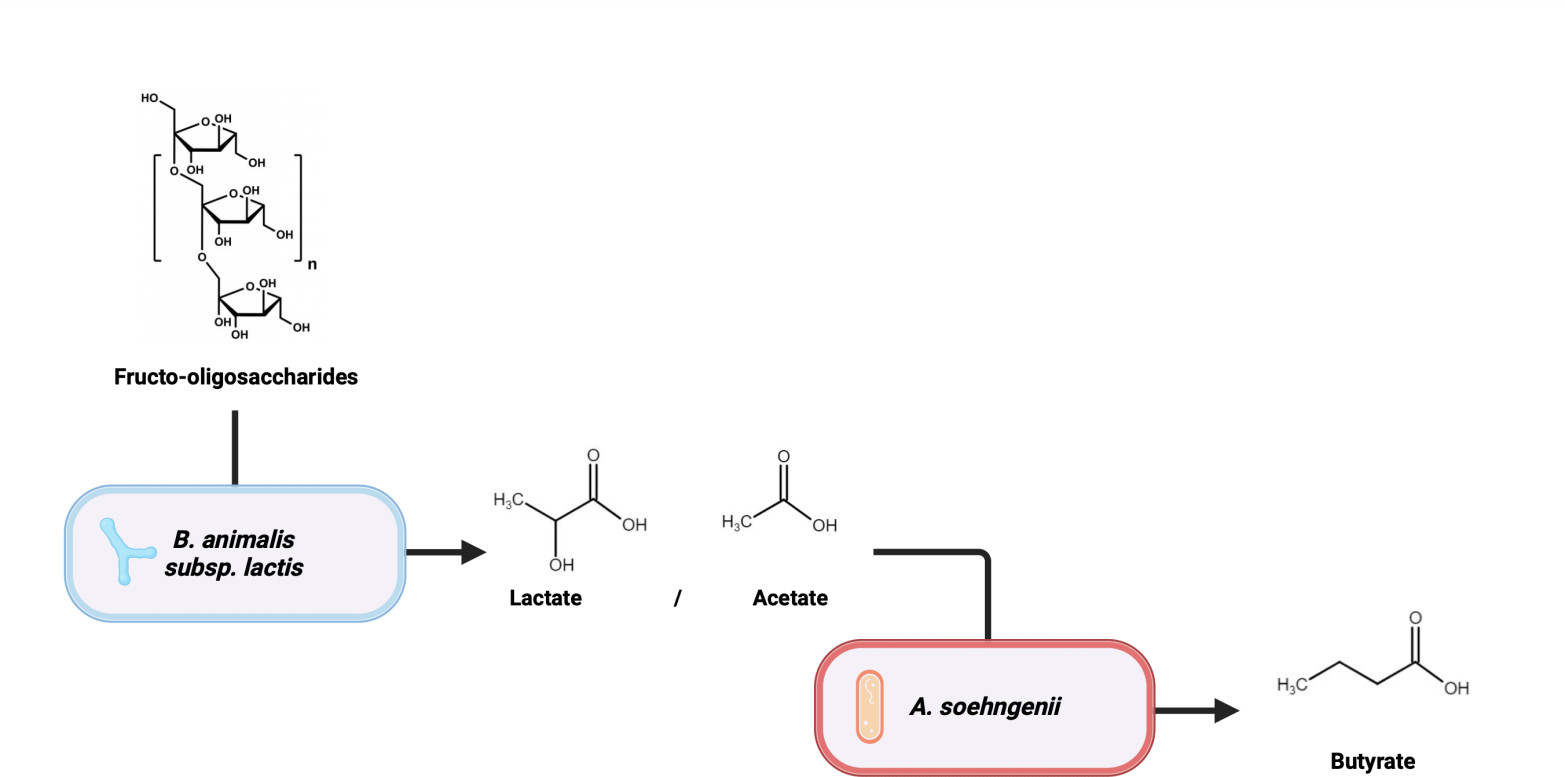
Trophic chain converting fructo-oligosaccharides to butyrate through *B. animalis* subsp*. lactis* and *A. soehngenii* subsequently.

Next-generation beneficial bacteria, such as *A. soehngenii* and *A. muciniphila*, have shown great potential in the field of metabolic diseases.^32 33^ *A. soehngenii* is a gut commensal with beneficial metabolic effects for the host organism, which are considered to be mediated at least in part through the production of butyrate.^33^ Among other effects, butyrate stimulates the release of glucagon-like peptide 1 (GLP-1) by L-cells in the duodenum, leading to improved insulin sensitivity.^33 34^ In vitro, *A. soehngenii* performs well in a syntrophic chain with certain *Bifidobacteria* species that use inulin-type fructans (eg*,* fructo-oligosaccharides (FOSs)) to produce acetate and lactate, which are substrates for *A. soehngenii* (figure 1).^35^

*A. muciniphila* is a mucin-degrading bacterium that resides in the intestinal mucus layer in a healthy gut and has numerous health benefits, such as maintaining a healthy gut barrier function, improving insulin sensitivity and exerting anti-inflammatory effects.^30 32 36 37^ A randomised controlled trial studying daily supplementation with *A. muciniphila* in overweight/obese, insulin-resistant human participants surprisingly showed that pasteurised *A. muciniphila* improved several metabolic parameters, such as insulin sensitivity, insulinemia, plasma cholesterol and several blood biomarkers of liver dysfunction and inflammation.^30 38^

In conclusion, the aim of the current treatment strategy is multifaceted. FOSs are metabolised by *B. animalis* subsp. *lactis* into lactate and acetate. Lactate and acetate, in turn, can be metabolised by *A. soehngenii* into butyrate. The latter metabolite increases GLP1 production in enteroendocrine L-cells, with subsequent beneficial metabolic effects. Vegan FMT is rich in butyrate-producers and has previously been shown to reduce hepatic inflammation.^22^ Furthermore, pasteurised *A. muciniphila* improves the gut barrier, reduces inflammation and exerts beneficial metabolic effects. This study is the first to study a combination of FMT and next-generation beneficial bacteria as a treatment for MASH.

### Primary objective

To investigate the therapeutic potential of the combination of *A. soehngenii*, pasteurised *A. muciniphila*, *Bifidobacterium animalis* subsp. *lactis*, FOSs and lyophilised faecal microbiota of vegan donors to reduce MASH, as assessed by histopathological scoring.

### Key secondary objectives

- Investigate the impact of the combination therapy on non-invasive outcomes of MASLD: multiparametric MRI of the liver and surrounding subcutaneous adipose tissue (MRI-PDFF, MR elastography and corrected T1), FibroScan elastography, controlled attenuation parameters, plasma aspartate aminotransferase, alanine aminotransferase, gamma-glutamyl transferase and alkaline phosphatase, the enhanced liver fibrosis (ELF) plasma panel.
- Examine the influence of the combination therapy on the hepatic mRNA expression of genes associated with MASLD and MASH.
- Evaluate the effect of the combination therapy on glycaemic control through continuous glucose monitoring (CGM) (Freestyle Libre 2, Abbott Diabetes Care, Maidenhead, UK) and Homeostatic Model Assessment of Insulin Resistance.
- Investigate the impact of the combination therapy on parameters of the metabolic syndrome associated with MASLD and MASH: plasma lipid profiles, lipidomics and metabolomics.
- Assess the impact of the combination therapy on parameters of gut barrier dysfunction through the analysis of plasma LPSs and faecal albumin.
- Investigate changes in faecal microbiota composition due to the combination.
- Examine the effect of the combination therapy on systemic low-grade inflammation and associated inflammatory pathways in liver tissue and plasma.
- Assess the impact of the combination therapy on quality of life using the Short Form Health Survey (SF-36) and the chronic liver disease questionnaire for non-alcoholic steatohepatitis (CLDQ-NASH).
- Evaluate the effects of the combination therapy on dietary intake through a detailed food frequency questionnaire available at mijn.voedingscentrum.nl/nl/eetmeter.

### Methods and analyses: participants, interventions and outcomes

#### Study design

**Figure 2:**
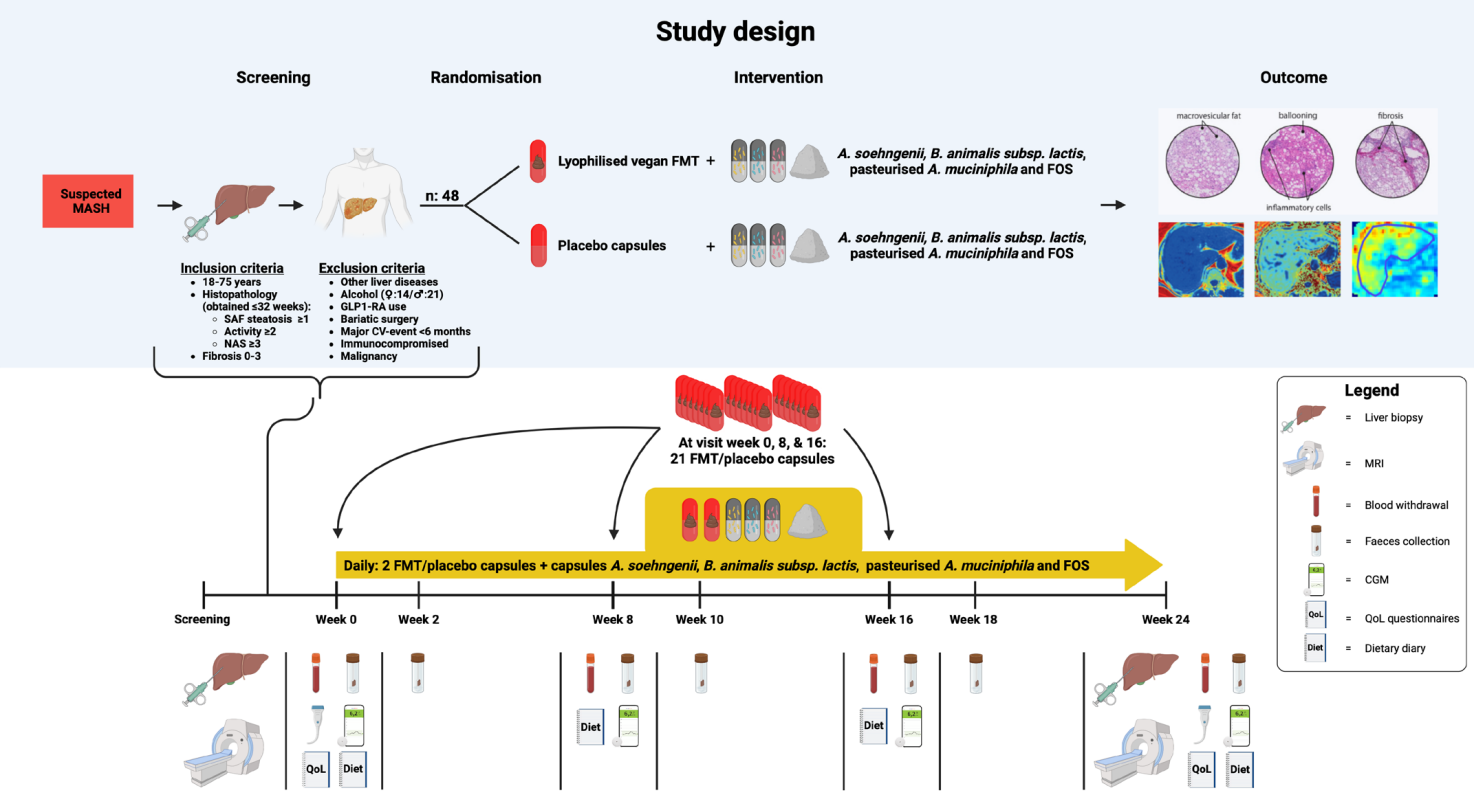
Overview of the study design. CGM, continuous glucose monitoring; CV-event, cardiovascular event; FMT, faecal microbiota transplantation; FOSs, fructo-oligosaccharides; GLP1-RA, glucagon-like peptide-1 receptor agonist; -MASH, metabolic dysfunction-associated steatohepatitis; NAS, NAFLD activity score; QoL, quality of life questionnaires; SAF, Steatosis, Activity, and Fibrosis scoring system.

The SYNCH trial is a randomised, double-blind, placebo-controlled, single-centre phase 2 trial (protocol V. 5). Participants will receive treatment for 24 weeks. All included individuals will daily receive *A. soehngenii*, pasteurised *A. muciniphila*, *B. animalis* subsp. *lactis* and FOSs. Participants are randomised 1:1 to receive either an FMT originating from lean, healthy vegan donors or a placebo FMT. Donor stool from vegans is cryopreserved, lyophilised and encapsulated to produce lyophilised faecal microbiota capsules (LFMCs). Participants will receive a loading dose of 21 LFMCs at baseline, week 8 and week 16. In addition, participants will take two LFMCs (or placebo capsules) daily for the entire 24 week study duration (figure 2).

#### Study setting

Participants are recruited through the outpatient clinics of the Amsterdam University Medical Centres (Amsterdam UMC) and affiliated secondary hospitals. Lean, healthy, vegan donors are recruited through advertisements in the hospital and on social media. All interventions and study visits will be conducted at Amsterdam UMC, location AMC, in the Netherlands. This centre features a specialised MASLD outpatient clinic and has extensive expertise in performing FMTs. The first participant was recruited on 25 April 2023. The end-of-study is scheduled for December 2025.

#### Eligibility criteria

##### Participant inclusion criteria

- Age 18–75 years.
- Active MASH: biopsy-proven NASH obtained up to 32 weeks before screening: SAF (Steatosis, Activity, Fibrosis scoring system) steatosis score ≥1, activity ≥2, Fibrosis <4; 50% of participants should at least have NASH fibrosis stage 1, 2, or 3 according to the NASH -Clinical Research Network (NASH CRN) fibrosis staging system based on tandem reading of two expert liver pathologists.
- Fluency in Dutch or English.
- Able to understand the information and give informed consent.

Exclusion criteria are displayed in online supplemental table 1.

### Interventions

#### Investigational products

At each of the first three visits, participants will ingest 21 LFMCs or placebo capsules. Each LFMC contains approximately 235 mg of lyophilised FMT (equivalent to 1500 mg of faecal suspension or 3 mL of fresh FMT (for fresh FMT, the dilution is 1:3)). Additionally, participants will take two LFMCs (or placebo capsules) daily for the entire 24 week study duration. Capsules are advised to be ingested with a glass of water on an empty stomach in the morning, preferably an hour before breakfast. The LFMCs are provided to the participants at the study visits and will be kept in the freezer (−20 to −4°C) by the participant.

Starting from the baseline visit until the end of the study (24 weeks later), participants will daily ingest 5 g of FOS powder (Sensus, Roosendaal, the Netherlands). Simultaneously, participants will daily ingest the capsules with doses of 10^9^ *A. soehngenii* CH-106 cells (Caelus Health, Amsterdam, the Netherlands; dosage based on previous studies, including a toxicology study),^18 39^ 10^10^ *B. animalis* subsp. *lactis* BLC1 cells (SynbioTec, Camerino, Italy) and 3×10^10^ cells of pasteurised *A. muciniphila* strain Muc^T40^(dosage based on European Food Safety Administration (EFSA) approval and obtained from the Akkermansia Company, Mont-Saint-Guibert, Belgium) for the entire 24 weeks. All participants receive extensive verbal and written instructions on correctly storing and ingesting the capsules and sachets with powder. Remaining capsules and sachets are counted at each study visit. If participants are required to take antibiotics, study visits are conducted at an earlier stage in order to minimise the period of non-treatment.

#### Preparation of LFMCs

##### Stool donors

Healthy, lean individuals (body mass index (BMI): 18–25 kg/m^2^) adhering to a stable plant-based vegan diet for at least 3 months prior to donation are recruited as stool donors through social media. Potential donors received information about the screening and donation processes before providing informed consent. The screening process involved a questionnaire, physical examination and a series of (microbiological) tests on blood and faecal samples in accordance with the International FMT guidelines.^41^ The donor screening consisted of three stages: (1) a questionnaire and physical exam, (2) stool screening for parasites and (3) stool and blood testing for the presence of pathogenic (multidrug-resistant) bacteria, viruses or haematological, hepatic or renal deviations. Eligible donors that passed the entire screening could donate stool for 2 months, whereafter they had to pass a complete rescreening (in line with guidelines for stool banking).^42^ Only faecal donations obtained and stored between two negative screenings are further processed into LFMCs.

##### Stool donor inclusion criteria

- Lean: BMI: 18–25 kg/m^2^.
- Aged 18 to 75 years.
- Adherence to a vegan diet >3 months.

##### Stool donor exclusion criteria

- Exclusion criteria are displayed in online supplemental table 2.

##### Processing donor stool

**Figure 3:**
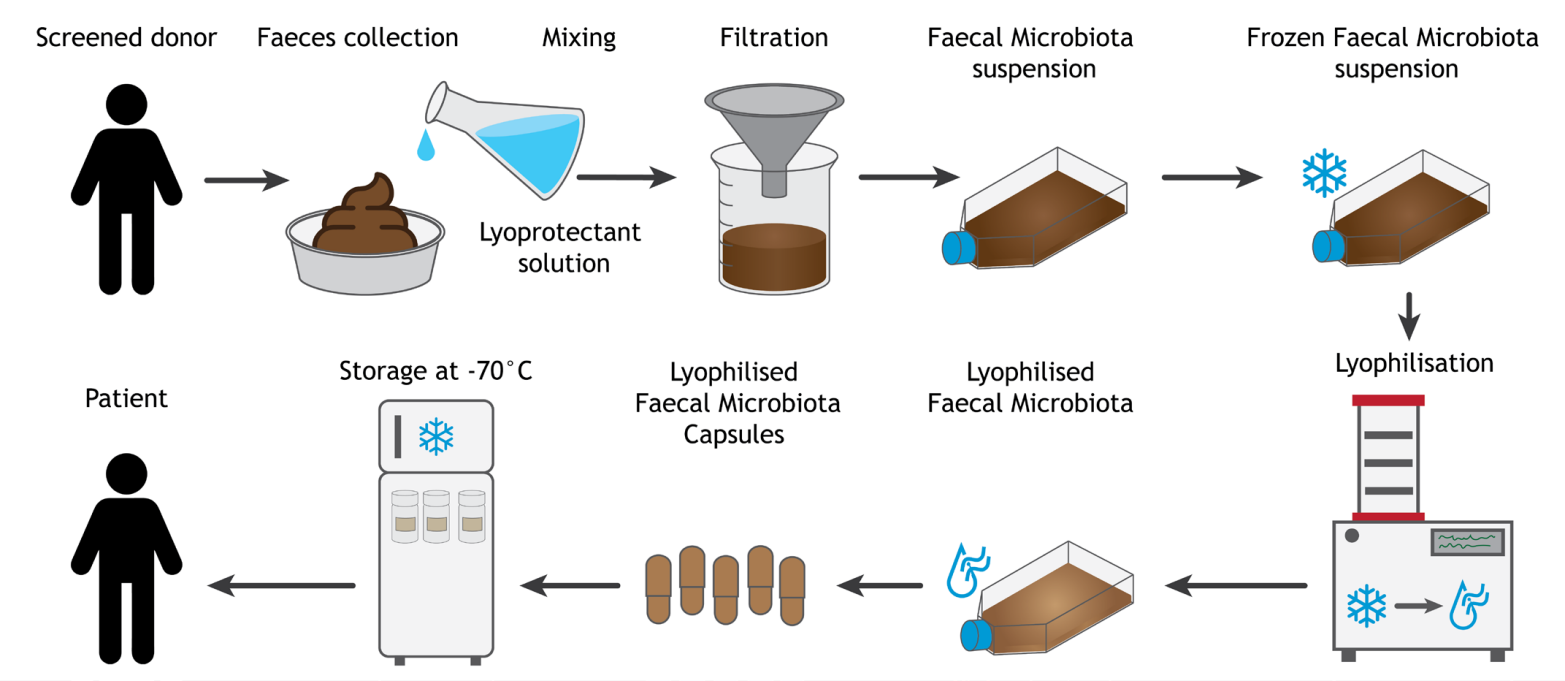
Production process of lyophilised faecal microbiota transplantation capsules.

Donor stool has to be delivered to the hospital within 2 hours of collection. The stool is kept cool and directly transferred to a refrigerator (2–8°C) on reception. To preserve microbial viability and prevent (unwanted) shifts in composition, faeces are homogenised in a 1:1 ratio with a lyoprotectant solution, which protects the microbes during both freezing and lyophilisation (figure 3). We tested different combinations of lyoprotectants and found that a combination of 10% trehalose and 5% maltodextrin best preserved bacterial viability, which has been reported by others as well.^43^ Subsequently, the faecal suspension is filtered through sterile non-woven gauzes in a metal funnel to remove any particulate matter. The faecal microbiota suspension is then transferred to sterile flasks and frozen at −70°C. These processing steps are performed as soon as possible after receipt of the donor stool and have to be completed within 6 hours of defaecation.

##### Lyophilisation and encapsulation of faecal microbiota

After successful re-screening of the stool donor after 2 months, collected frozen faecal microbiota suspensions are released from quarantine and further processed. First, faecal microbiota are lyophilised for 48 hours under a vacuum with the condenser set at −80°C. The lyophilised faecal microbiota cake is transferred to a mortar, ground to a fine powder, and mixed homogeneously with a lubricant to improve the flow properties of the powder. Lyophilised faecal microbiota from different donors are pooled to increase microbiota diversity and the reproducibility of the intervention.^44^ If necessary, extra maltodextrin is added as filler to fill the capsules evenly.

The powder mixture is encapsulated in white opaque size 0 Enprotect capsules (Lonza Capsugel), which protect the microbes from gastric acid on ingestion. These capsules performed best in preserving the bacterial viability of the LFMCs during preclinical disintegration and dissolution testing. As the lyophilised powder is very fine and difficult to completely remove from the outer capsule shell, the LFMCs are double-encapsulated in red opaque hypromellose capsules size 00. These red outer capsules also provide an indistinguishable look for the LFMC and placebo capsules. Placebo capsules are filled with a mixture of the excipients used in the lyoprotectant solution, trehalose and maltodextrin, and the lubricant.

Finally, LFMCs (or placebos) are packaged in tamper-proof, opaque high-density polyethylene containers with desiccant to preserve a dry environment. Containers are labelled in accordance with Good Manufacturing Practice Annex 13 and stored in the freezer at −70°C until delivery to study participants. During a 2 year stability study, storage at −70°C did not result in any significant reduction in bacterial viability, compared with storage in a refrigerator (2–8°C) or room temperature (15–25°C), probably in part due to the dry freezer environment.

### Study procedures

#### Liver biopsy

Percutaneous liver biopsies will be conducted at screening and at the end of the 24 week study. In addition, if a liver biopsy is performed within the 32 weeks prior to study inclusion, the biopsy will be considered at screening. At 24 weeks, another liver biopsy will be performed to assess the impact of the treatment. Evaluation will be based on the NASH-CRN classification, considering steatosis, inflammation and ballooning on H&E slides, and fibrosis on Sirius red-stained slides.^2^ To minimise the interobserver variability, all biopsies will undergo a tandem read by two specialised liver pathologists, blinded to any other parameter. Furthermore, histopathological features will be quantified using digital whole slide image analysis and using second harmonic generation and two-photon excitation microscopy with artificial intelligence analysis. Finally, hepatic tissue will be snap-frozen for RNA sequencing. Differential gene expression will be assessed over time and by treatment allocation.

#### Multiparametric MRI and vibration-controlled transient elastography

Prior to baseline and at 24 weeks, participants will undergo a multiparametric MRI of the liver and an MRI of visceral and subcutaneous fat using a 3T Philips Ingenia MRI scanner.^45^ The total scan time will be approximately 45 min to estimate visceral and subcutaneous adipose tissue depot volume, hepatic and pancreatic fat content, as well as hepatic fibrosis and inflammation.^45^,^47^ Individuals will be screened for contra-indications for MRI prior to inclusion in this study. In addition, vibration-controlled transient elastography (FibroScan) will be performed to investigate liver stiffness and steatosis.

#### Gut microbiota and metabolite composition

At baseline and subsequently at 2, 8, 10, 16, 18 and 24 week time points, participants will collect faecal samples that will be stored in the freezer at −70°C. Faecal samples will be used for 16S rRNA sequencing, metabolomic profiling and the assessment of calprotectin and albumin as surrogate markers reflecting intestinal barrier function.

#### Fasted blood draw and glucose monitoring

Fasted blood samples will be collected at baseline, 8, 16 and 24 weeks. At baseline and 24 weeks, comprehensive analyses will be conducted on liver enzymes, indicators of glycaemic control, lipid profiles and a broad spectrum of general and NASH-specific parameters. Additionally, in-depth immunological, lipidomic and metabolomic analyses will be performed on the collected blood samples. The blood withdrawals at 8 and 16 weeks are primarily for safety assessment through a complete blood count and a basic metabolic panel to assess kidney and liver function, electrolyte levels and blood glucose.

Moreover, continuous glucose measurements will be conducted at home using portable devices (Freestyle Libre 2, Abbott Diabetes Care). This will occur during a consecutive 7 day period after each study visit.

#### Food diary and quality of life questionnaires

Participants will maintain a diary documenting their daily food intake for 5 days in the week preceding and following each study visit (www.voedingscentrum.nl/eetmeter). Additionally, they will complete the SF-36 questionnaire and CLDQ-NASH assessing physical endurance and quality of life at both baseline and 24 weeks.^48 49^

### Outcomes

#### Primary outcome

Histopathological improvement of liver histology in individuals with MASH and fibrosis stage 0–3, with improvement defined as the reduction of steatohepatitis by ≥1 SAF-A point (the activity part of the SAF scoring system) with no worsening of liver fibrosis or improvement of ≥1 stage in liver fibrosis with no worsening of steatohepatitis.

#### Secondary outcomes

- Non-invasive outcomes of MASLD, that is, multiparametric MRI of the liver and surrounding subcutaneous adipose tissue (MRI-PDFF, MR elastography (MRE) and corrected T1), FibroScan elastography and controlled attenuation parameter and plasma panel ELF panel.
- Change in plasma from baseline to the end of treatment in regard to: liver function, blood counts, lipid profiles, inflammatory and immunological markers, endocrine, metabolic and lipidomic profiles, microbial-derived metabolites and markers for gut barrier integrity.
- Liver gene expression profile: lipogenic, inflammatory and fibrogenic pathways.
- Glycaemic control, insulin resistance, BMI, waist circumference and percentage body fat.
- Microbiome readouts (composition, engraftment and strain tracking) and metabolites, faecal SCFA-composition and faecal albumin.
- Quality of life assessed through the SF-36 (36-items) questionnaire and the CLDQ-NAFLD (non-alcoholic fatty liver disease).
- Food diary.

### Statistical methods

#### Sample-size calculation

Based on our FMT pilot study^22^ and the study of *A. soehngenii* in individuals with metabolic syndrome,^34^ we conducted a power analysis to calculate the number of participants necessary to detect a 25% reduction in our primary outcome parameter, reversal of steatosis and necroinflammation following donor FMT combined with the next-generation beneficial bacteria treatment. For a desired alpha of 0.05 and a desired power of 0.8, a sample size of 24 participants per arm will be required; ergo, 48 participants in total.

### Participant allocation and blinding

Participants will be allocated to either LFMC or placebo capsules, with allocation stratified based on metformin use, proton pump inhibitor use, and fibrosis grade (fibrosis grade F0-1 vs F2-3). This stratification aims to equalise the potential effects of these agents and the fibrosis grade on disease attenuation. The study uses computer-generated block randomisation with block sizes of 2:4:6 to minimise the likelihood of uneven sample sizes. Unblinding will occur once all participants have completed the study, and the database has been locked.

A pharmacist conducts randomisation through the Clinical Electronic Data Capture (EDC) CASTOR database. The pharmacist then provides the opaque capsules to the study physician. All other study personnel, with the exception of the pharmacist, are blinded until the database is locked. In case of an emergency unblinding, an independent physician will unblind the participant.

### Statistical analysis

Statistical analyses will be conducted using SPSS statistical analysis software and R-studio. The normality of the data will be assessed with a Kolmogorov-Smirnov test, and data transformation will be applied if the data is not normally distributed. Primary analyses of the studied parameters will be performed between baseline and endpoint interventions using a two-tailed one-sample t-test or χ^2^ test. In cases where the data are not normally distributed and transformation is not feasible, the Mann-Whitney U test will be employed. Further associations will be evaluated using Pearson’s rank correlation test or Pearson’s χ^2^ test.

Paired categorical data will be examined using McNemar’s test. Paired continuous data will undergo analysis with a paired t-test if normally distributed, and a Wilcoxon signed-rank test will be applied if the data are not normally distributed.

In R, statistical packages using machine-learning algorithms will be employed to identify metabolites and microbiota that best predict a favourable response. An elastic net machine learning classification algorithm, in conjunction with a stability selection procedure, will be employed to identify biological features exhibiting differential changes between the two treatment groups.

### Data collection and management

Data will be collected at five visits by trained, local research staff. Data will be entered into the Clinical EDC CASTOR database. The Clinical Monitoring Centre of the Amsterdam UMC will monitor the conduct of study procedures, data entry and case report forms. All participant data will be coded. The code-to-participant translation file is kept in a file only accessible to study personnel. Research data will be stored for 15 years. An independent data monitoring committee will perform an interim analysis when the first 16 participants have finished the trial.

### Ethics and dissemination

Ethical approval was obtained from the Medical Ethics Committee of the Amsterdam UMC, in accordance with the Declaration of Helsinki (updated version October 2013, Fortaleza, Brazil) and with the Medical Research Involving Human Subjects Act. All participants will provide written informed consent. The trial is registered on clinicaltrials.gov (NCT05821010). Results of the primary study outcomes will be disseminated through a manuscript published in a peer-reviewed journal.

### Adverse events and safety

Participants will be required to daily ingest LFMCs along with pre- and probiotics for a duration of 24 weeks. Additionally, liver biopsies, FibroScans and MRIs will be conducted two times, a CGM device will be applied four times, and blood samples will be drawn on five occasions.

FMT is considered a safe treatment, and studies employing LFMCs have lower complication rates than FMT administered via a nasoduodenal tube.^50^,^53^ The most common side effects of FMT are mild and self-resolving and include abdominal pain, nausea, diarrhoea and flatulence. FOS and *B. animalis* subsp. *lactis* can have the same side effects, also often mild and resolved within weeks.^54^,^57^ Exploratory studies on *A. soehngenii* and pasteurised *A. muciniphila* did not report side effects after 4 weeks and 3 months of administration, respectively.^30 58^ Moreover, the liver biopsy procedure may result in minor bleeding in fewer than 2 in 1000 cases.^59^ To mitigate this risk, coagulation will be assessed prior to the biopsy.

All potential complications are thoroughly communicated to potential participants verbally and through the participant information form. Adverse events, defined as any undesirable experience occurring to a participant during the trial, irrespective of their connection to the trial, will be reported by the investigator.

A serious adverse event is any untoward medical occurrence or effect that:

- Results in death.
- Is life-threatening (at the time of the event).
- Requires hospitalisation or prolongation of existing inpatient hospitalisation.
- Results in persistent or significant disability or incapacity.
- Is a congenital anomaly or a birth defect.
- Is there any other important medical event that did not result in any of the outcomes listed above due to medical or surgical intervention but could have been based on appropriate judgement by the investigator.

### Patient and public involvement

The study was reviewed by a patient who serves on the Medical Ethics Committee at Amsterdam UMC.

### Discussion

The objective of this study is to investigate the potential of continuous FMT treatment via capsules supplemented with next-generation beneficial bacteria as a viable treatment for MASH. We hypothesise that FMT and next-generation beneficial bacteria might not only complement each other but also work synergistically to a surplus effect. The next-generation beneficial bacteria used in this study are less prevalent in patients with MASH. Thus, it is to be expected that these bacteria thrive less in the gut of individuals with MASLD and subsequently have less potential to exert their beneficial effects when given as a solo treatment.^10 12 22^ The introduction of a richer microbial community through FMT creates a more favourable environment for next-generation beneficial bacteria, which could theoretically make these bacteria thrive and engraft more effectively. This could enhance their efficacy, as they benefit from a bacterial network in which they naturally occur in greater abundance. Vice versa, the next-generation beneficial bacteria can stimulate the symbiotic bacteria present in the FMT. We, therefore, postulate that the combination of FMT and next-generation beneficial bacteria has the potential to lead to a more significant improvement in the pathophysiology of MASH than when applied separately.

Many studies suggest that the gut microbiome plays a pivotal role in the multifaceted pathophysiology of MASLD. To date, several studies have investigated the potential of pre- and probiotics as a treatment for MASLD. While some studies have reported positive outcomes with different formulations of pre-, pro- and synbiotics, these studies unfortunately fall short of accurately assessing the potential of gut microbiome modulation in the treatment of MASH in humans.^60 61^ First, the majority of controlled trials that report a reduction in MASLD following conventional probiotic therapy in humans use surrogate endpoints, such as anthropometric parameters, blood tests or controlled attenuation parameters by FibroScan. Second, the majority of studies conducted in humans have only employed the use of *Lactobacillus* or *Bifidobacterium* spp., or a combination thereof, which often are not found in large numbers in the human gut. Of note, certain *Lactobacillus* species may even worsen MASLD by the production of ethanol.^26^ New next-generation beneficial bacteria that derive from the human intestinal tract are increasingly identified as having beneficial properties for the metabolic system.^32 62^ However, despite the growing body of evidence on the potential of next-generation beneficial bacteria in the treatment of a plethora of metabolic diseases in humans and MASLD in mice, studies using next-generation beneficial bacteria in humans with MASLD are yet to be performed. Thirdly, the sample sizes of all probiotic studies are relatively small, possibly attributable to their investigator-initiated nature.

Although several studies indicate the beneficial effects of lean donor FMT on cardiometabolic parameters in metabolic syndrome, only three have specifically examined its therapeutic potential in MASLD.^20^,^22^ Xue *et al* demonstrated a moderate reduction in liver fat attenuation scores assessed by FibroScan post-FMT.^20^ This effect was more pronounced in lean MASH patients, supporting the hypothesis that gut microbiota are causally involved in MASH development independent of caloric overload. However, it should be noted that a fat attenuation score is a variable surrogate marker of hepatic steatosis and, therefore, requires caution in interpretation, especially in small sample sizes. Moreover, the absence of true randomisation, short 1 month follow-up, and incomplete reporting of confounding factors limit the interpretability of this trial. The second study, a placebo-controlled trial from Craven *et al*, did not show changes in liver fat content after FMT, as assessed by MRI-PDFF.^21^ However, it remains debatable if a single FMT of 2 g has the potential to significantly improve MASLD within a 6 week follow-up, especially when studied in only 15 subjects.^63^ Nevertheless, the study did show improvements in gut barrier function after allogenic FMT, which is postulated to play a key role in the gut-liver axis and the development of MASLD.^64^ Finally, Witjes *et al* compared three infusions via a nasoduodenal tube of allogenic versus autologous FMT every 8 weeks.^22^ Allogenic FMT resulted in a reduction in histopathological hepatic necroinflammation and an increase in the faecal abundance of the butyrate-producers *F. prausnitzii* and *A. soehngenii*. However, the modest sample size and the broad inclusion criteria (obese patients with simple steatosis sufficed) are significant limitations in the latter study.

The current study is robust due to its randomised, double-blind design, and the use of liver biopsy as a primary outcome measure, complemented by non-invasive tests such as MRI-PDFF and MRE to mitigate sample error. The use of capsules to administer FMT has been successfully applied in *Clostridium difficile* infections, yet its application in the context of metabolic diseases remains less explored.^65^ However, there are multiple advantages associated with the use of capsules in comparison to the conventional FMT method via nasoduodenal tube. First, capsules are more patient-friendly and safe.^51 52^ Second, FMT via a duodenal tube is administered intermittently, allowing the microbiome to revert to its original state between treatments, which may scrutinise treatment effects. Continuous delivery of FMT via capsules could enhance therapeutic efficacy. Finally, capsules are closest to a regular treatment and, therefore, closest to translation into therapy.

The duration of 24 weeks might be a limitation as it raises questions about its sufficiency for demonstrating effects, particularly on liver fibrosis; however, previous pharmacological studies have shown efficacy within similar timeframes.^66 67^ Another limitation is the absence of a control group or pure placebo group, which hampers the ability to draw definitive conclusions about the treatment’s efficacy. This can be partly overcome by comparing the placebo arm biopsies from previous clinical trials with a comparable study population and duration.

To the best of our knowledge, this is the first study to combine FMT with next-generation beneficial bacteria to treat any metabolic disease. The study will help answer the question if gut microbiome modulation can be a feasible treatment for MASH. It will also test the novel hypothesis of providing a beneficial environment for beneficial bacteria using FMT. The modulation of the gut microbiome is a safe and potentially inexpensive treatment with mild and few side effects. Further understanding of its applicability in common metabolic diseases may be of great value.

## supplementary material

10.1136/bmjopen-2024-088290 online supplemental file 1

10.1136/bmjopen-2024-088290 online supplemental file 2

10.1136/bmjopen-2024-088290 online supplemental file 3

10.1136/bmjopen-2024-088290 online supplemental file 4

10.1136/bmjopen-2024-088290 online supplemental file 5
