# Supplementary material for: Randomised double-blind placebo-controlled trial protocol to evaluate the therapeutic efficacy of lyophilised faecal microbiota capsules amended with next-generation beneficial bacteria in individuals with metabolic dysfunction-associated steatohepatitis
Source: BMJ Open. 2025 Jan 9;15(1):e088290. doi: 10.1136/bmjopen-2024-088290 (PMC11784342; doi:10.1136/bmjopen-2024-088290)
Supplement: online supplemental file 1 [file bmjopen-15-1-s001.docx]

# Keywords / MeSH terms

Non-alcoholic Fatty Liver Disease (NAFLD)

(new term: metabolic dysfunction-associated steatotic liver disease, MASLD)

Faecal Microbiota Transplantation

Akkermansia muciniphila

Anaerobutyricum soehngenii (formerly known as: Eubacterium Hallii)

Bifidobacterium animalis
