# Supplementary material for: Randomised double-blind placebo-controlled trial protocol to evaluate the therapeutic efficacy of lyophilised faecal microbiota capsules amended with next-generation beneficial bacteria in individuals with metabolic dysfunction-associated steatohepatitis
Source: BMJ Open. 2025 Jan 9;15(1):e088290. doi: 10.1136/bmjopen-2024-088290 (PMC11784342; doi:10.1136/bmjopen-2024-088290)
Supplement: online supplemental file 2 [file bmjopen-15-1-s002.docx]

### Supplemental Table 1: Exclusion criteria participants

| - Concomitant other liver diseases, such as (but not limited to): hepatitis B and/or C, auto-immune hepatitis, Wilson’s disease, primary sclerosing cholangitis, primary biliary cholangitis, alpha-1-antitripsine deficiency and hemochromatosis |
| --- |
| - Current or history of significant alcohol consumption for a period of more than 3 consecutive months within 1 year before screening (defined as a daily average consumption of more than 2 international units for females and more than 3 international units for males) |
| - Liver cirrhosis or hepatocellular carcinoma |
| - History of liver transplant, current placement on a liver transplant list |
| - Use of pre-, pro- or synbiotics |
| - Use of systemic antibiotics 3 month prior to randomization |
| - Use of tamoxifen, methotrexate or amiodarone |
| - Prior or planned bariatric surgery |
| - Active GLP-1 receptor agonist treatment |
| - Bleeding disorder |
| - International normalized ratio (INR) of prothrombin time >1.4 or platelet count <100 10^9^/L at screening |
| - Anti-platelet/coagulant therapy that cannot be temporarily discontinued |
| - Any major cardiovascular event within 6 months prior to screening (e.g. myocardial infarction, cerebrovascular accident) |
| - Prolonged compromised immunity (e.g. recent cytotoxic chemotherapy, HIV-infection with a CD4 count < 240 cells/mm^3^) |
| - Active or prior history of invasive malignancy (except for curatively treated in situ carcinomas [e.g., cervix] or non-melanoma skin cancer) unless a complete remission was achieved |
| - Surgery scheduled for the trial duration period, except for minor surgical procedures |
| - Pregnancy or breastfeeding |
| - Any condition which, in the investigator’s opinion, might jeopardize participants’ safety or compliance with the protocol |
| - Participation in another concomitant clinical trial. |
