# Supplementary material for: Randomised double-blind placebo-controlled trial protocol to evaluate the therapeutic efficacy of lyophilised faecal microbiota capsules amended with next-generation beneficial bacteria in individuals with metabolic dysfunction-associated steatohepatitis
Source: BMJ Open. 2025 Jan 9;15(1):e088290. doi: 10.1136/bmjopen-2024-088290 (PMC11784342; doi:10.1136/bmjopen-2024-088290)
Supplement: online supplemental file 3 [file bmjopen-15-1-s003.docx]

### Supplementary Table 2: Exclusion criteria stool donors (adaptation from Keller et al, 2021^38^)

| Medical history and lifestyle |
| --- |
| - - History of chronic, systemic autoimmune disorders with GI involvement |
| - - History of neurological/neurodegenerative disorders |
| - - History of psychiatric conditions |
| - - Presence of chronic low-grade inflammation or metabolic syndrome (NCEP criteria) |
| - - Presence of T1DM, T2DM or hypertension |
| - - History of cholecystectomy |
| - - Use of any medication including proton pump inhibitors, antibiotics and pro-/prebiotics in the past three months or during the study period |
| - - Illicit drug use (MDMA/amphetamine/cocaine/heroin/GHB) in the past three months or use during the study period |
| - - Use of >5 units of alcohol daily on average in the past three months or daily use of >2 units of alcohol during the donation period |
| Infectious disease |
| - - History of, or known exposure to HIV, hepatitis B (HBV) or C virus (HCV), syphilis, human T-lymphotropic virus (HTLV) I and II, malaria, trypanosomiasis, tuberculosis, strongyloidiasis, non-successfully eradicated *Helicobacter pylori* |
| - - Any currently active infection or those of relevance within the past 6 months |
| - - Live attenuated vaccine within the past 8 weeks |
| - - Risk of transmission of diseases caused by prions |
| At risk behaviour |
| - - Current or previous intravenous drug use |
| - - Ongoing high risk sexual behaviour within the past 6 months (anonymous sexual contacts; sexual contacts with prostitutes, drug addicts, individuals with HIV, viral hepatitis, syphilis; work as prostitute; history of sexually transmittable disease) |
| - - Travel to high‐risk foreign countries within the past 6 months |
| - - Medical treatment in poorly hygienic conditions within the past 6 months |
| - - Current occupation in a setting facilitating acquisition of potential pathogens (e.g., veterinarian, animal attendant, gamekeeper, prison worker) |
| - - Body tattoo, piercing, earring, acupuncture within the past 6 months |
| - - Major surgery within the past 6 months |
| - - Contact with human blood (e.g., accident, needle stick injury) within the past 6 months |
| - - Previous prison term |
| - - Previous reception of tissue/organ transplant |
| - - Transfusion of blood products (e.g., packed red cells, plasma, platelets, immunoglobulins) within the past 6‐month |
| Intestinal health |
| - - History of IBS (according to Rome IV criteria), IBD, functional chronic constipation, coeliac disease, other chronic GI disorders |
| - - History of, or high risk for, GI cancer or polyposis |
| - - Gastrointestinal symptoms within the past 3 months (e.g., diarrhoea, constipation, haematochezia, vomiting, abdominal pain) |
| Blood and faeces testing |
| - - Positive Dual Faeces Test (DFT) for *Giardia lamblia*, *Dientamoeba fragilis*, *Entamoeba histolytica*, *Microsporidium* spp. ^b^, *Cryptosporidium* spp., *Cyclospora*, *Isospora* or *Blastocystis hominis^a^*. Positive microscopic exam for eggs, cysts and larves (e.g. helminth eggs) |
| - - Presence of faecal bacterial pathogens *Salmonella* spp., *Shigella* spp., *Campylobacter* spp., *Yersinia* spp., *C. difficile*, *H. pylor***i**, *shigatoxigenic Escherichia coli* (STEC) stx1/stx2, *Aeromonas* spp., *Pleisiomonas shigelloides^b^* or *Vibrio* (if visited or residing in tropical country within the past 6 months) in faeces. |
| - - Presence of extended spectrum beta-lactamase (ESBL) producers, Carbapenemresistant *Enterobacteriaceae* (CRE), vancomycin-resistant *Enterococci* (VRE) or methicillin-resistant *Staphylococcus aureus* (MRSA) in faeces |
| - - Presence of *Rotavirus*, *Norovirus* I/II, *Enterovirus^b^*, *Parechovirus^b^*, *Astrovirus^b^*, *Sapovirus^b^*, *Adenovirus^b^* or SARS-CoV-2 in faeces |
| - - Positive serologic test for HIV 1/2, *Hepatitis A virus* (HAV), HBV, HCV, *Hepatitis E virus* (HEV), HTLV 1/2, active *Cytomegalovirus* (CMV)^b^ or active *Epstein–Barr virus* (EBV)^b^, *Strongyloides stercoralis* or *Treponema pallidum* |
| - - Abnormal liver or renal function (creatinine, ureum, ASAT, ALAT, AF, GGT, bilirubin) or impaired immunity (CRP, haemoglobin, MCV, leukocytes, thrombocytes). |
| - - Elevated faecal calprotectin |
| ^a^*Colonisation with (a low amount of) B. hominis is not considered an exclusion criterium, but the effects of transmission should be monitored.* |
| ^b^*Screening tests for Microsporidia, Pleisiomonas shigelloides, Enterovirus, Parechovirus, Astrovirus, Sapovirus, Adenovirus CMV and EBV are only advised for immunocompromised individuals (including toxoplasmosis).* |
