# Supplementary material for: Randomised double-blind placebo-controlled trial protocol to evaluate the therapeutic efficacy of lyophilised faecal microbiota capsules amended with next-generation beneficial bacteria in individuals with metabolic dysfunction-associated steatohepatitis
Source: BMJ Open. 2025 Jan 9;15(1):e088290. doi: 10.1136/bmjopen-2024-088290 (PMC11784342; doi:10.1136/bmjopen-2024-088290)
Supplement: online supplemental file 4 [file bmjopen-15-1-s004.pdf]

The SYNCH trial

SYNbiotics and Conditioned Fecal Microbiota Transplantation to Treat Non-Alcoholic Steatohepatitis

Department of Vascular Medicine, Amsterdam UMC location AMC

Department of Gastroenterology and Hepatology, Amsterdam UMC location AMC

D3-314

PROTOCOL TITLE SYNbiotics and Conditioned FMT to treat non-alcoholic steatohepatitis

**Protocol ID**	SYNCH
**Short title**	SYNbiotics and Conditioned FMT to treat non-alcoholic steatohepatitis
**Version**	5.0
**Date**	March 27, 2023
**Coordinating investigator/project leader**	dr. A.G. Holleboom, internist<br>a.g.holleboom@amsterdamumc.nl<br>0205666612; 27413
**Principal investigator(s) (in Dutch: hoofdonderzoeker/ uitvoerder)**	drs. Q.J.J. Augustijn, MD<br>q.j.augustijn@amsterdamumc.nl<br>dr. H.J. Herrema<br>h.j.herrema@amsterdamumc.nl<br>dr. A. Grefhorst<br>a.grefhorst@amsterdamumc.nl<br>dr. A.G. Holleboom, internist<br>a.g.holleboom@amsterdamumc.nl<br>0205666612; 27413
**Sponsor (in Dutch: verrichter/opdrachtgever)**	Amsterdam UMC, location AMC
**Subsidising party**	Health~Holland - Topsector Kennisinstituut - Public Private Partnership
**Independent expert**	Nordin Hanssen, internist<br>n.m.j.hanssen@amsterdamumc.nl

**Laboratory sites**	Experimental Vascular Medicine, Amsterdam UMC<br>Dr. H.J. Herrema<br>h.j.herrema@amsterdamumc.nl<br><br>Dr. A. Grefhorst<br><br>a.grefhorst@amsterdamumc.nl
**Pharmacy**	*Not applicable*

PROTOCOL SIGNATURE SHEET

Name	Signature	Date
**Head of Department:**<br>Prof. dr. J.M. Prins		
**Project leader:**<br>Dr. A.G. Holleboom		

TABLE OF CONTENTS

SUMMARY 9
1. INTRODUCTION AND RATIONALE 15
2. OBJECTIVES 21
3. STUDY DESIGN 21
4. STUDY POPULATION 24
4.1 Population (base) 24
4.2 Inclusion criteria 24
4.3 Exclusion criteria 25
4.4 Sample size calculation 27
5. TREATMENT OF SUBJECTS 28
5.1 Investigational product/treatment 28
5.2 Use of co-intervention 29
5.3 Escape medication 29
6. INVESTIGATIONAL PRODUCT 30
6.1.1 Investigational product name and formulation 30
6.1.2 Other product name(s) and formulation(s) 30
6.2 Name and description of investigational product(s) 31
6.3 Summary of findings from non-clinical studies 31
6.4 Summary of findings from clinical studies 32
6.5 Summary of known and potential risks and benefits 34
6.6 Description and justification of route of administration and dosage 35
6.7 Dosages, dosage modifications and method of administration 35
6.8 Preparation and labelling of Investigational Medicinal Product 36
6.9 Drug accountability 38
7. NON-INVESTIGATIONAL PRODUCT 40
7.1 Name and description of non-investigational product(s) 40
7.2 Summary of findings from non-clinical studies 40
7.3 Summary of findings from clinical studies 40
7.4 Description and justification of route of administration and dosage 41
7.5 Dosages, dosage modifications and method of administration 41
7.6 Preparation and labelling of Non Investigational Medicinal Product 41
7.7 Drug accountability 42
8. METHODS 43
8.1 Study parameters/endpoints 43
8.1.1 Main study parameter/endpoint 43
8.1.2 Primary outcome 43
8.1.3 Secondary outcomes 43
8.1.4 Other study parameters 44
8.2 Randomisation, blinding and treatment allocation 44
8.3 Study procedures 44
8.4 Withdrawal of individual subjects 47

8.4.1 Specific criteria for withdrawal 47
8.5 Replacement of individual subjects after withdrawal 47
8.6 Follow-up of subjects withdrawn from treatment 47
8.7 Premature termination of the study 47
9. SAFETY REPORTING 48
9.1 Temporary halt for reasons of subject safety 48
9.2 AEs, and SAEs 48
9.2.1 Adverse events (AEs) 48
9.2.2 Serious adverse events (SAEs) 48
9.2.3 Recording and Reporting of Adverse Events 49
9.2.4 Recording of AEs/SAEs 50
9.2.5 Relationship 50
9.2.6 Severity 51
9.3 Follow-up of adverse events 51
9.4 [Data Safety Monitoring Board (DSMB) / Safety Committee] 51
10. STATISTICAL ANALYSIS 52
11. ETHICAL CONSIDERATIONS 53
11.1 Regulation statement 53
11.2 Recruitment and consent 53
11.3 Benefits and risks assessment, group relatedness 53
11.4 Compensation for injury 56
11.5 Incentives 57
12. ADMINISTRATIVE ASPECTS, MONITORING AND PUBLICATION 58
12.1 Handling and storage of data and documents 58
12.2 Monitoring and Quality Assurance 58
12.3 Amendments 58
12.4 Annual progress report 59
12.5 Temporary halt and (prematurely) end of study report 59
12.6 Public disclosure and publication policy 59
13. STRUCTURED RISK ANALYSIS 60
13.1 COVID-19 61
14. Amendments 62
14.1 First amendment 62
15. REFERENCES 63

LIST OF ABBREVIATIONS AND RELEVANT DEFINITIONS

**ABR**	**General Assessment and Registration form (ABR form), the application form that is required for submission to the accredited Ethics Committee; in Dutch: Algemeen Beoordelings- en Registratieformulier (ABR-formulier)**
**AE**	**Adverse Event**
**AR**	**Adverse Reaction**
**CA**	**Competent Authority**
**CCMO**	**Central Committee on Research Involving Human Subjects; in Dutch: Centrale Commissie Mensgebonden Onderzoek**
**CV**	**Curriculum Vitae**
**DSMB**	**Data Safety Monitoring Board**
**ESBL**	**Extended spectrum beta-lactamase producing bacteria**
**EU**	**European Union**
**EudraCT**	**European drug regulatory affairs Clinical Trials**
**FDA**	**(U.S) Food and Drug Administration**
**FMT**	**Fecal microbiota transplantation**
**FOS**	**Fructo-oligosaccharides**
**GCP**	**Good Clinical Practice**
**GMP**	**Good Manufacturing Practice**
**GDPR**	**General Data Protection Regulation; in Dutch: Algemene Verordening Gegevensbescherming (AVG)**
**GLP1**	**Glucagon-like peptide-1**
**HACCP**	**Hazard Analysis and Critical Control Points**
**IB**	**Investigator's Brochure**
**IC**	**Informed Consent**
**IMP**	**Investigational Medicinal Product**
**IMPD**	**Investigational Medicinal Product Dossier**
**ITF**	**Inulin-type fructans**
**LFMT**	**Lyophilized fecal microbiota transplantation**
**MDR**	**Multidrug resistant**
**METC**	**Medical research ethics committee (MREC); in Dutch: medisch-ethische toetsingscommissie (METC)**
**NAFLD**	**Non-alcoholic fatty liver disease**
**NASH**	**Non-alcoholic steatohepatitis**
**NGE**	**Next generation enteric [capsules]**

**(S)AE**	**(Serious) Adverse Event**
**SAF**	**In SAF-score: Steatosis, Activity, Fibrosis score.**
**SPC**	**Summary of Product Characteristics; in Dutch: officiële productinformatie IB1-tekst**
**Sponsor**	**The sponsor is the party that commissions the organisation or performance of the research, for example a pharmaceutical company, academic hospital, scientific organisation or investigator. A party that provides funding for a study but does not commission it is not regarded as the sponsor, but referred to as a subsidising party.**
**SUSAR**	**Suspected Unexpected Serious Adverse Reaction**
**UAVG**	**Dutch Act on Implementation of the General Data Protection Regulation; in Dutch: Uitvoeringswet AVG**
**WMO**	**Medical Research Involving Human Subjects Act; in Dutch: Wet Medisch-wetenschappelijk Onderzoek met Mensen**

SUMMARY

Rationale:

The prevalence and severity of non-alcoholic fatty liver disease (NAFLD) is increasing at an alarming rate, because ever more patients are living longer with the two main factors that drive NAFLD: obesity and type 2 diabetes mellitus (T2DM). To date, no treatment is available for progressive NAFLD stages, i.e. non-alcoholic steatohepatitis (NASH) and NASH-fibrosis, yet the gut microbiome and its metabolites are emerging as an innovative treatment approach.

Data from our pilot study indicate that 1) fecal microbiota transplantation (FMT) of vegan donors may have anti-inflammatory effects in NASH, and 2) vegan FMT may enrich the gut of the recipient with *Anaerobutyricum soehngenii*, a microbe thought to reduce NASH via glucagon-like peptide 1 (GLP1) and the conversion of lactate and acetate into the beneficial butyrate.

Another microbe, pasteurized *Akkermansia muciniphila* (pAkk), may reduce NASH because it enhances barrier function of the gut, potentially reducing inflammatory tone across the gut-liver axis in NASH.

A third probiotic, *Bifidobacterium animalis, subsp. lactis,* is known to convert prebiotics, e.g. fructooligosaccharides (FOS), into lactate and acetate. Not only does FOS supplementation induce a major increase of *Akkermansia muciniphila* in the gut, by adding FOS and *B. animalis, subsp. lactis,* we create a trophic chain, in which FOS is converted via *B. animalis, subsp. lactis* into lactate and acetate, which in turn are converted by *A. soehngenii* into butyrate.

Because NASH may be addressed best with combined approaches, we aim to combine the synbiotic treatment of *A. soehngenii* and *Akkermansia muciniphila* with *Bifidobacterium animalis, subsp. lactis* and FOS, in combination with FMT from vegan donors who were conditioned by using an inulin-rich plant extract. We hypothesize that this combined therapy will reduce NASH and liver fibrosis in patients with NASH and NASH-fibrosis.

Objective:

Main objective

To investigate the therapeutic potential of *A. soehngenii* and pasteurized *A. muciniphila* combined with *B. animalis* subsp. *lactis* and fructo-oligosaccharides with and without conditioned vegan lyophilized fecal microbiota transplantation (LFMT) capsules to reduce NASH in patients with NASH and NASH-fibrosis.

Secondary objective

To investigate the mechanisms of *A. soehngenii* and pasteurized *A. muciniphila* combined with *B. animalis* subsp. *lactis* and FOS with and without conditioned vegan LFMT capsules in reducing NASH in patients with NASH and NASH-fibrosis.

Study design:
Double-blind randomized placebo-controlled intervention study.

Study population:
Patients between age 18-75 years with biopsy-proven NASH obtained up to 32 weeks before screening based on tandem reading of two expert liver pathologists: SAF Steatosis score ≥1, Activity ≥2, Fibrosis <4; 50% of participants should at least have NASH fibrosis stage 1, 2 or 3 according to the NASH CRN fibrosis staging system.

Exclusion criteria are: Current or history of significant alcohol consumption for a period of more than 3 consecutive months within 1 year before screening (significant alcohol consumption is defined as more than 2 units/day for females and more than 3 international units/day for males, on average; 1 international unit contains ±14 grams of alcohol), liver cirrhosis or hepatocellular carcinoma, hepatitis B and/or C, auto-immune hepatitis, Wilson's disease, primary sclerosing cholangitis, primary biliary cholangitis, alpha-1-antitripsine deficiency and hemochromatosis, history of liver transplant, current placement on a liver transplant list, use of pre-, pro- or synbiotics, use of systemic antibiotics 3 month prior to randomization, use of tamoxifen, methotrexate or amiodarone, prior or planned bariatric surgery, diabetes mellitus with active GLP-1 receptor agonist treatment, bleeding disorder, International normalized ratio (INR) of prothrombin time >1.4 or platelet count <50 10^9/L at screening, anti-platelet/coagulant therapy use which cannot be temporarily discontinued, any major cardiovascular event within 6 months prior to screening (e.g. myocardial infarction, cerebrovascular accident), prolonged compromised immunity (e.g. recent cytotoxic chemotherapy, HIV-infection with a CD4 count < 240), active or prior history of invasive malignancy (except for curatively treated in situ carcinomas [e.g., cervix] or non-melanoma skin cancer) unless a complete remission was achieved, surgery scheduled for the trial duration period, except for minor surgical procedures in the opinion of the investigator, pregnant or nursing women, any condition which, in the investigator's opinion, might jeopardize participants' safety or compliance with the protocol, and participation in another concomitant clinical trial.

A total of 48 patients will receive synbiotic treatment with *A. soehngenii*, pasteurized *A. muciniphila*, *B. animalis* subsp. *lactis* and fructo-oligosaccharides, and will be randomized 1:1

to treatment with FMT or placebo, stratified for proton-pump inhibitor use, metformin use, and histopathological fibrosis score.

Intervention:

Recipient participants

From the first FMT until completion of the study, all recipient participants will receive an oral daily single dose of 5 g FOS. Subsequently, participants will be randomized to lean donor FMT capsules or placebo (blinded design)[1]. At baseline (day 0) and at week 8 (day 56) and week 16 (day 112) participants will ingest 21 LFMT or placebo capsules. In addition, participants will daily take 2 capsules of LFMT or placebo during the whole study period (24 weeks). The study visits will be 8 weeks apart, although a margin of -3 days to +3 days is implemented to take participants schedule and availability into account. We have previously observed that gut microbiota composition in the recipient is affected up to 8-12 weeks after donor FMT[1], so this time window ensures a stable donor gut microbiota composition during the study.

During the 24-week intervention period, all participants will take daily doses of 10^9 *A. soehngenii* CH-106 cells (dosage based on previous studies including toxicology study)[2,3], 10^{10} *B. animalis* subsp*. lactis* BLC1 (a well-studied strain marketed as a probiotic by Sacco SRL) and $3x10^{10}$ pasteurized *A. muciniphila* ATCC BAA-835[T] cells (dosage based on EFSA approval and obtained from A-Mansia Biotech).

Percutaneous liver biopsies will be performed as a part of screening when participants are theoretically eligible for participation, unless a liver biopsy has been performed in the previous 36 weeks. A tandem-read by two liver pathologists blinded to any other result will determine if patients will be included in the study. At 24 weeks, another liver biopsy will be performed to examine the effect of the FMT, which will also be reviewed by two liver pathologists (again blinded to any other result). The NASH-CRN classification[4] will be assessed on H&E slides, for steatosis, inflammation and ballooning, and with a Sirius red–stained slide for evaluation of fibrosis. RNA for RNA-sequencing will be isolated. Differential gene expression will be assessed over time (baseline and 24 weeks) and by treatment allocation (vegan donor FMT versus placebo).

Feces will be collected at baseline, and after week 2, 8, 10, 16, 18, and 24, in order to investigate the changes in gut microbiome. Also, markers of gut barrier function will be assessed.

Blood will be collected at baseline and at 8, 16 and 24 weeks to investigate common liver enzymes, indicators of glycemic control, lipids, and general and more NASH-specific inflammation parameters.

A multiparametric MRI of liver (MRI-PDFF, MR elastography, corrected T1) and of visceral and subcutaneous fat will be performed before baseline and after 24 weeks to estimate visceral and subcutaneous adipose tissue depot volume, hepatic fat content as well as hepatic fibrosis and inflammation.
Continuous glucose measurements will be performed at home using portable devices, during a consecutive period of 1 week (7 days) in the week before baseline, week 1, 9, 17 and 25.

Vegan donors
Fecal donor samples will be collected, lyophilized ('freeze-dried'), and encapsulated (see paragraph 6.8). Thereafter, the LFMT capsules will be stored at -80°C. Donors will be extensively screened for infectious diseases.

Main study parameters/endpoints:

Primary outcome
To demonstrate that *A. soehngenii* combined with pasteurized *A. muciniphila* and *B. animalis* subsp. lactis, FOS and conditioned vegan LFMT capsules reduce NASH as defined by an improvement of liver histology in individuals with NASH and fibrosis stage 0-3, with improvement defined as reduction of steatohepatitis by ≥1 SAF-A point and no worsening of liver fibrosis, or improvement in ≥ 1 stage liver fibrosis and no worsening of steatohepatitis.

Secondary outcomes
To demonstrate that the combined treatment improves:

- non-invasive outcomes of NAFLD, i.e. multiparametric MRI of liver and surrounding subcutaneous adipose tissue (MRI-PDFF, MR elastography, corrected T1), FibroScan Elastography and Controlled Attenuation Parameters, and plasma panel Enhanced Liver Fibrosis (ELF) panel, pro-C3.
- change in blood markers from baseline to end of treatment, namely: liver enzymes (i.e. alanine amino transferase (ALT), aspartate amino transferase (AST), gamma glutamyl transferase (GGT), alkaline phosphatase (ALP)), inflammatory blood markers (i.e. leukocytes, monocytes, CRP, Il-1(β), Il-6, Il-11, Il-17, Il-32, TNF-α, IFN-γ, other inflammatory markers), SCFA (i.e. propionate, butyrate, acetate), lactate, ethanol, plasma lipids (i.e. LDL, HDL, triglycerides, total cholesterol), albumin, kreatinine, hemoglobin, FGF-21, adiponectin, leptin, lipopolysaccharides, estrogen, vitamin B12, folate acid,zonulin, and other metabolomic and lipodomic outcomes.
- microbiome read outs (composition, engraftment, strain tracking) and metabolites, fecal SCFA-composition, and fecal albumine.

- glycemic control, insulin resistance, body weight/BMI, waist circumference and percentage body fat
- MetSy criteria / %
- SCFA production and GLP1 release
- liver gene expression profile: lipogenic, inflammatory and fibrogenic pathways.
- liver pathology, histopathological features, immunofluorescence and assessment of pathophysiological proteins.
- NAFLD histology as assessed with a deep-learned algorithm scoring whole slide images of liver biopsies.
-
- Continuous glucose monitoring (4x 7 consecutive days)
- quality of life (general (SF36) and NAFLD/NASH-specific (CDLQ-NAFLD)).

Nature and extent of the burden and risks associated with participation, benefit and group relatedness:

This study is a follow up to our previous intervention study aiming to examine the effect of FMT, now lyophilized and encapsulated, and in combination with synbiotic treatment and their underlying mechanisms of gut microbiota, in individuals with NASH.

FMT-donors
Fecal microbiotica will be collected of healthy, vegan donors. FMT-donors will be screened for infectious diseases through blood withdrawal and fecal analysis, which will be repeated after 1-2 months, and thereafter every 3 months.

Recipient participants
Individuals with NASH will be recruited from our outpatient clinic. After the screening is completed, participants will be telephoned with further instructions.
All individuals will have a total of four study visits, and time invested will approximately be 30 hours per person. At baseline and after 24 weeks, we will collect anthropometric data (e.g. body weight, height, waist circumference, bioelectrical impedance etc.). In addition, participants will hand in fecal samples at week 0, 2, 8, 10, 16, 18, and 24. Blood will be checked at week 0, 8, 16, and 24. Participants will, moreover, keep a diary of their daily food intake for 5 days in the week before and after study visits. In addition, they will fill in questionnaires physical endurance and quality of life at baseline and 24 weeks.
Participants will visit the study center at baseline (week 0), week 8, 16, and 24. On the first three visits, participants will ingest 21 LFMT capsules (or placebo) containing approximately

235mg of freeze-dried FMT (this equals 1500mg of fresh FMT) each. In addition, participants will ingest 2 LFMT-capsules (or placebo) daily for the duration of the study (24 weeks).
From the baseline visit until the end of the study (24 weeks later), patients will daily take 5 g FOS (powder). After baseline visit, participants will also ingest capsules of 10^9 *A. soehngenii* CH-106 cells, 10^{10} B. animalis subsp. *lactis* BLC1 and $3x10^{10}$ pasteurized *A. muciniphila* cells. All of these will be taken in the morning. All bacterial strains and FOS are well researched on dosage, toxicity and adverse events, and show no serious hazards.

Our study participants will be referred to the AMC intervention radiology department for liver biopsy to assess NASH histology severity, pathophysiological pathways and gene expression in liver tissue. As an experienced interventional radiologist will perform the liver biopsy ultrasound-guided, the risk (comprising mostly bleeding from the biopsy site) of complications will be very low (<0.1 %). Moreover, local hemostasis after the procedure will be observed and bleeding disorders or anticoagulant therapy are an exclusion criterion. Based on our previous experience, we might encounter ±1 patient with a complication (most likely small bleeding) in our entire study. To date, a liver biopsy is still the gold standard for discerning the severity of NASH and provides the possibility to study pathophysiological pathways and gene expression.
In addition, a multiparametric MRI of the liver and MRI of visceral and subcutaneous fat will be performed with a 3T Philips Ingenia MRI scanner. Participants will be screened for contra-indications for MRI prior to inclusion in this study. Participants will be provided with metal free hospital clothing if necessary. The total scan time will be approximately 45 minutes to estimate visceral and subcutaneous adipose tissue depot volume, hepatic and pancreatic fat content, as well as hepatic fibrosis and inflammation.
Last, continuous glucose measurements will be performed at house using portable devices, during a consecutive period of 1 week (7 days) in the week before baseline, week 1, 9, 17 and 25.

1. INTRODUCTION AND RATIONALE

NAFLD: a significant liver disease

The prevalence and severity of NAFLD - excessive hepatic fat accumulation without excessive alcohol consumption – are increasing at an alarming rate, because more and more patients are living longer with the two main factors that drive NAFLD: obesity and T2DM.[5,6] 20-30% of the global population including The Netherlands[7] has some stage of NAFLD[8], and in patients with T2DM, this is even as high as 60-80%.[5] NAFLD is regarded as the hepatic component of the metabolic syndrome (MetSy) and T2DM.[9–11] It can silently progress from simple steatosis to NASH (prevalence 2-3%) and NASH-related fibrosis, and ultimately to cirrhosis and hepatocellular carcinoma (fig.1).[5,9–11]

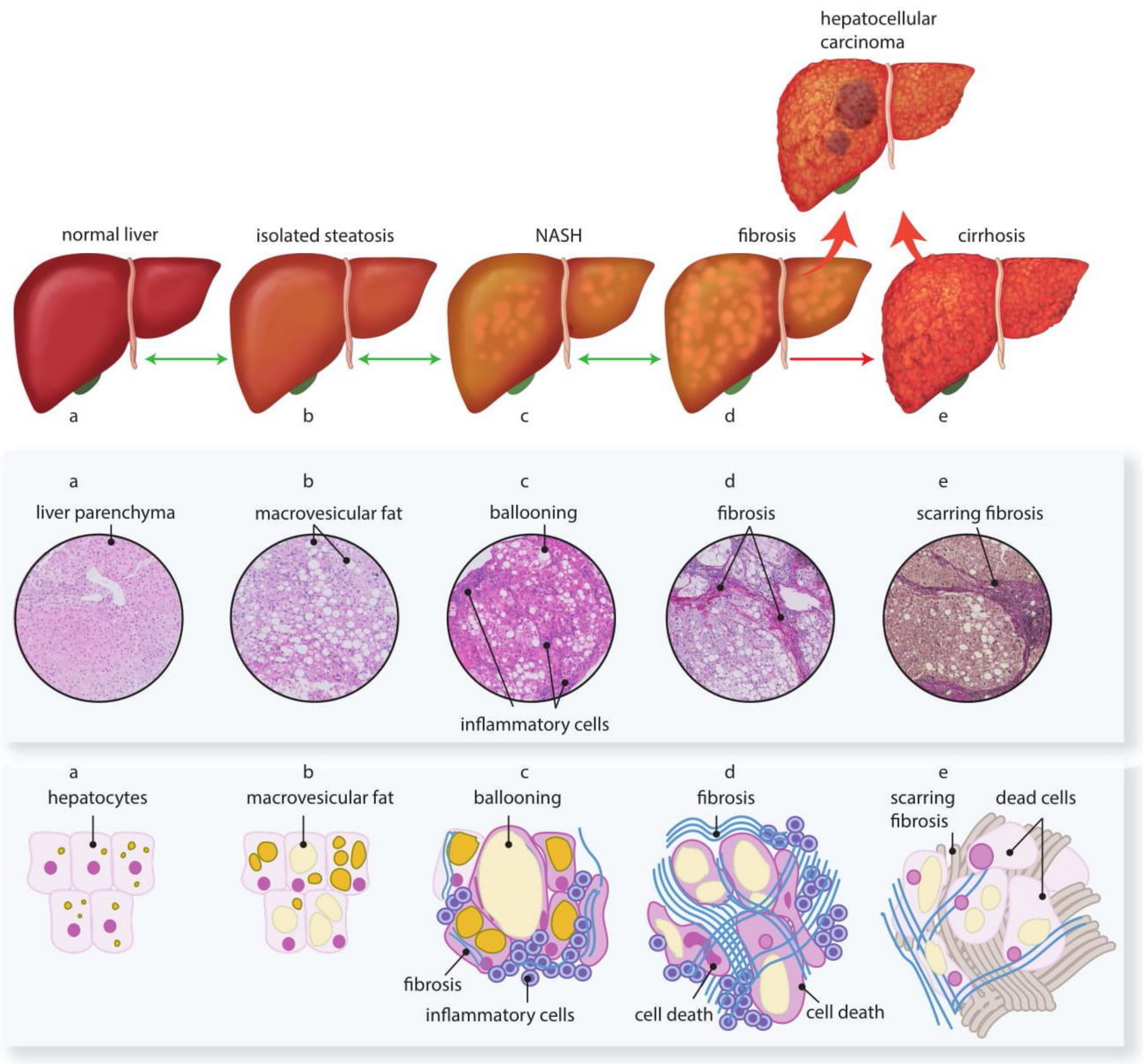

Figure 1. The disease spectrum of NAFLD

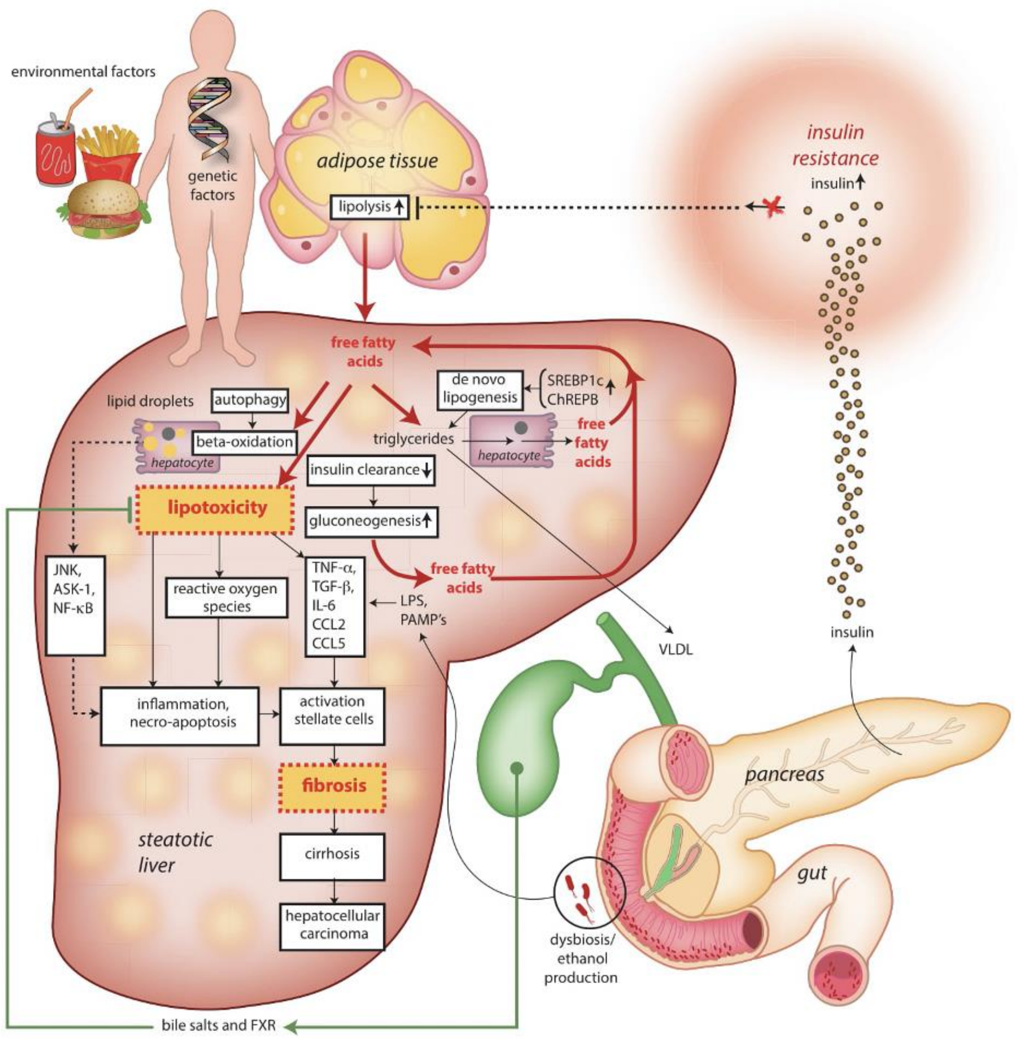

Figure 2. The pathogenesis of NAFLD-NASH. NAFLD-NASH has a complex pathogenesis with a multiplicity of pathways. Yet centrally stands insulin resistance, causing lipolysis in peripheral adipose tissue, resulting in increased hepatopetal free fatty acid (FFA) flux. These FFAs are stored as triglycerides in hepatic lipid droplets. This in turn reduces hepatic insulin sensitivity, increasing hepatic gluconeogenesis and glucose secretion contributing to hyperglycemia, but also to subsequent intrahepatic conversion of glucose to even more lipids. Systemic insulin resistance amplifies this process, since high plasma insulin and glucose upregulate Sterol Regulatory Element-Binding transcription factor 1c and Carbohydrate-Responsive Element-Binding Protein signaling, further stimulating lipogenesis. This drives hepatic lipotoxicity, triggering NASH, necroinflammation and activation of hepatic stellate cells, initiating fibrogenesis.
From Ruissen, Holleboom, European Journal of Endocrinology 2020

Progression into the fibrotic stages of NAFLD is strongly associated with liver-related and overall mortality[12,13] and increased risk of atherosclerotic cardiovascular disease[14,15], the latter most likely via induction of dyslipidemia. Although NAFLD-NASH has a complex pathogenesis with a multiplicity of pathways, it is thought that insulin resistance is a crucial trigger since it causes lipolysis in peripheral adipose tissue, resulting in increased hepatopetal free fatty acid (FFA) flux.[16–18] This drives hepatic lipotoxicity, triggering NASH, necroinflammation[19–21] and activation of hepatic stellate cells, initiating fibrogenesis.[9] (for detail, see figure 2).

NAFLD: paucity of treatments and the potential of the gut microbiome

To date, no treatment is available for progressive NAFLD stages.[11,22] However, based on insights from observational studies and animal experiments, the gut microbiome and its metabolites are emerging as an innovative approach to treat this challenging metabolic liver disease, Potentially detrimental alterations in gut microbial composition have frequently been observed in NAFLD: abberant microbiota composition, often depleted in butyrate producers, reduced diversity, small intestinal bacterial overgrowth and signs of increased gut permeability.[11,23–26] This project aims to study the effects of the combination of bacterial strains on NAFLD.

Anaerobutyricum soehngenii

We recently published the first vegan fecal microbiota transplantation study in patients with NASH and aberrant microbial composition.[27] This pilot study found anti-inflammatory effects of vegan FMT into NASH patients, with a trend towards reduced hepatic necro-inflammation (fig.3). Upon vegan FMT engraftment, the gut microbiome of the NASH patients was enriched in *Anaerobutyricum soehngenii* (also/formerly known as *Eubacterium hallii)* and other butyrate-producing Lachnospiracae (fig.4).[27] Furthermore, in obese mice, *A. soehngenii* treatment improved insulin sensitivity and reduced intrahepatic triglyceride content.[28] Of note, *A. soehngenii*[29] is well known to not only convert sugars but also lactate and acetate into butyrate, a short chain fatty acid that may protect against NASH.[23,28] Butyrate may reduce the inflammatory component of NAFLD by promoting differentiation of regulatory T cells.[30] It also has an anti-migratory effects on neutrophils[31], and in the gut, it protects epithelial barrier integrity[32], both potentially protective effects on NASH. Moreover, since lactate is found to be increased in T2DM; the efficient conversion of lactate into butyrate by *A. soehngenii* is expected to contribute to its beneficial actions.

Bifidobacterium.animalis subsp. *lactis*

Another species thought to be benifical is *B.animalis* subsp. *lactis* of which the well-studied strain BLC1 is marketed as a probiotic and well characterized.[33] In vitro studies have shown that this strain can form a trophic chain with *A. soehngenii* to accelerate butyrate production from FOS. In addition, the BLC1 strain can in conjuction with *A. soehngenii* improve butyrate production in a model of the small intestine (Deyaert et al 2022, in preparation). Moreover, *B.animalis* subsp *lactis* with FOS has been shown to improve intestinal discomfort and promote anti-inflammatory properties.[34]

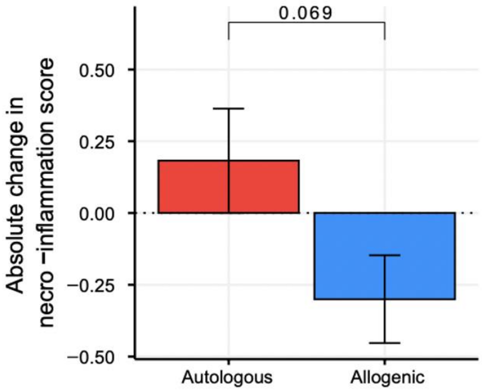

Figure 3. Changes in necro-inflammation score of liver histology following either autologous (i.e. from the patients with NASH, ergo control; red) or allogenic (i.e. from vegan donors; blue) FMT in patients with NASH.
From Witjes et al, Hepatology Communications 2020.

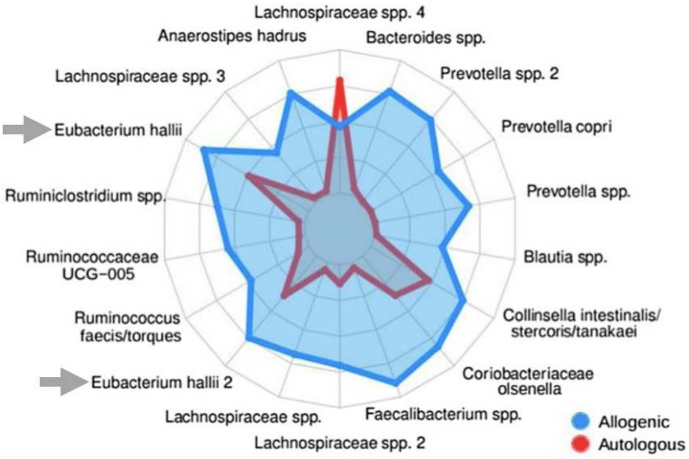

Figure 4. Radar plots of significantly altered fecal microbial strains following either autologous (red) or allogenic (blue) vegan FMT. Grey arrows indicate higher E. hallii species (now known as A. soehngenii).
From Witjes et al, Hepatology Communications 2020.

Akkermansia muciniphila

Pasteurized *A. muciniphila* is involved in host immunological homeostasis at the gut mucosa, improvement of gut barrier function and contributes to metabolic health. Indeed, in a study assessing the effects of pasteurized *A. muciniphila* on diet-induced metabolic disorders in mice, it was not only found that pasteurization did not diminish the beneficial effects, but it also unexpectedly enhanced the beneficial impact of *A. muciniphila* on insulin resistance and dyslipidemia.[35] Also, *A. muciniphila* and *A. soehngenii* work in a bidirectional syntrophy. A. soehngenii produces butyrate and pseudovitamin B_{12}. Pseudovitamin B_{12}, in turn, stimulates *A. muciniphila* to convert of acetate into propionate, thereby stimulating A. soehngenii to its production of butyrate and pseudovitamin B_{12}.[36] In addition, a randomized, double-blind, placebo-controlled proof-of-concept study in overweight/obese insulin-resistant human volunteers showed that the daily supplementation with pasteurized *A. muciniphila* at a daily

dose of 10^{10} cells (based upon CFU count) for 12 weeks improved several metabolic parameters such as insulin sensitivity, insulinemia, plasma total cholesterol, as well as relevant blood markers for liver dysfunction and inflammation while serum lipopolysaccharide (LPS) levels were decreased, indicative of improved barrier function.[37] A recent study in a high fat induced fatty liver mouse model showed that administration of *A. muciniphila* cells prevented fatty liver disease by regulation of the expression of genes that regulate fat synthesis and inflammation in the liver.[38] Since these studies all found that pasteurized *A. muciniphila* cells were similar or more efficient than the same amount of live *A. muciniphila* cells, we aim to use pasteurized *A. muciniphila* ATCC BAA-835^T cells in the present study. Of importance, these cells have recently received EFSA approval based on the extensive toxicological safety assessment.[39]

The used probiotic bacteria may reduce NAFLD via glucagon-like peptide 1

Recently studies found an interesting mechanism by which *A. soehngenii* may reduce NASH. In patients with MetSy, of which NAFLD is considered the hepatic equivalent, duodenal administration of *A. soehngenii* strongly increased glucagon-like peptide 1 (GLP1) secretion from the enteric endocrine L-cells, potentially relating to the observed reduced plasma glucose fluctuations (fig.5).[40] Of note, *A. muciniphila* administration induced GLP1 secretion in a mouse model.[41] Interestingly, clinical evidence is mounting that enhancing GLP1 may be a plausible approach to reduce NASH. In a subset of patients from the phase 2A LEAN trial with a GLP1-receptor agonist, it was shown that in addition to weight loss and improved glycemic control, insulin-resistance driven hepatopetal FFA-flux – the main contributor to lipid overload in NAFLD - was reduced.[42] In a recent Ph2B trial with a next generation GLP1 receptor agonist, NASH histology improved and signs of fibrosis regression were noted.[43] Thus among the aims of our studies is the explore the effects of the combined bacterial strains on GLP1 secretion.

Combination therapy may be required to treat NAFLD; the potential of synbiotic treatment

Yet of note, in larger phase 3 designs, some other single-drug studies have recently reported futility for NASH.[11,44] It is therefore quite broadly anticipated by both experts in the field as well as pharmaceutical companies that combination therapy may be more effective for NASH, as it is for other elements of MetSy such as T2DM, hypertension and dyslipidemia.[11]

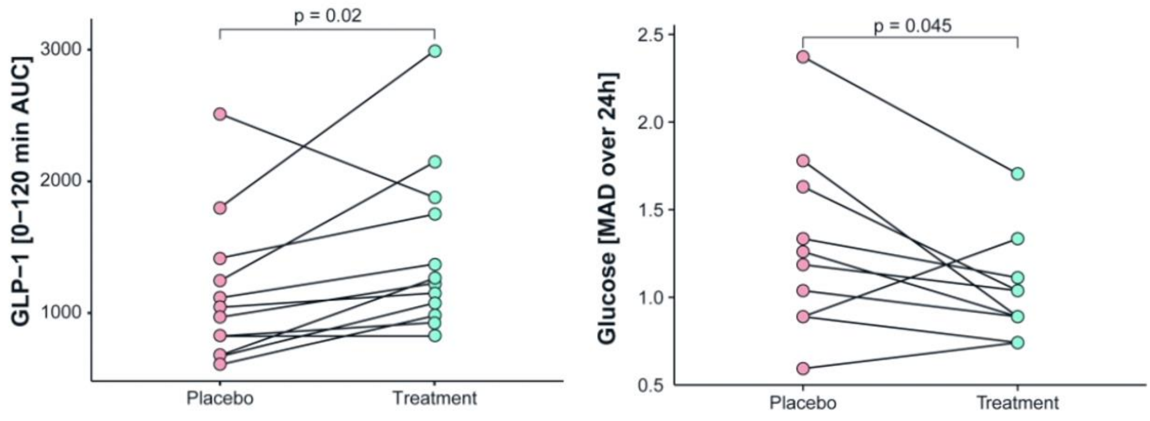

Figure 5. Duodenal A. soehngenii infusion in 12 patients with metabolic syndrome. Left panel: increased plasma GLP1 during mixed-meal test; right panel: median absolute deviation of continuous glucose measurements over the first 24 hours after placebo/treatment intervention. From Koopen et al, Gut 2021

This notion may also hold true when aiming to tap into the therapeutic potential of the gut microbiome for NASH. In particular, candidate therapeutic bacteria may be potentiated when combined with prebiotics, i.e. non-digestible food substances, which can be fermented by bacteria, promoting their growth. Such combinations are termed synbiotics. Early evidence from murine studies and pilot studies with synbiotic cocktails in NASH patients underscore the feasibility of this approach. NASH-fibrosis was reduced by a cocktail of 4 prebiotics and 4 probiotics in a high fat- choline-deficient diet mouse model.[45] In addition, ALT and TNFα were reduced in patients with NAFLD who received a synbiotic cocktail of 7 bacterial strains with the prebiotic fructo-oligosaccharides (FOS; oligomer of <10 fructose molecules).[46] In another pilot study in patients with NASH, a 12-week treatment with a synbiotic cocktail of 5 probiotic bacteria and fructo-oligosaccharides reduced ALT and liver stiffness on elastography.[47] Together these reports are supportive, yet the murine study did not decipher any mechanisms underlying the protective effects and the human studies e.g. did not provide liver histology, the gold standard in therapeutic development for NASH.

Furthermore, FOS is degraded to lactate and acetate by Bifidobacteria spp. such as *Bifidobacterium longum* or *B. animalis* subsp. *lactis,* a well-studied strain applied as a probiotic.[48,49] The generated lactate and acetate has shown to be effectively converted by *A. soehngenii* into butyrate in *in vitro* experiments, including simulated ileum and colon models at ProDigest (Deyaert et al 2022, manuscript in preparation). Such a cross-feeding mechanism can play a role in the colon ecosystem and contribute to the combined bifidogenic/butyrogenic effect observed after addition of FOS to the diet.[50,51] In this context a recent human intervention study should be noted where administration of *B. animalis* subsp. *lactis* with FOS was found to improve intestinal discomfort and promote anti-inflammatory properties.[34]

In our study, we will apply the complementary mode of actions of the individual components in the synbiotic treatment, the established effects of their combinations, and their expected impact on both upper intestinal tract and colon. We hypothesize that for NASH, synbiotic combination treatment will change the tipping point in the gut microbiome in order to reduce steatohepatitis and liver fibrosis.[52]

2. OBJECTIVES

Primary objective
To investigate the therapeutic potential of *A. soehngenii* and pasteurized *A. muciniphila* combined with *B. animalis* subsp. *lactis* and FOS with and without conditioned vegan Lyophylized fecal microbiota transplantation (LFMT) capsules to reduce NASH in patients with fibrotic NASH.

Secondary objective
To investigate the mechanisms of *A. soehngenii* and pasteurized *A. muciniphila* combined with *B. animalis* subsp. *lactis* and FOS with and without conditioned vegan LFMT capsules in reducing NASH in patients with fibrotic NASH.

3. STUDY DESIGN

Study visits:
Participants who are foreseen to fulfil the inclusion / exclusion criteria for enrolment into the clinical study will be informed and interviewed. When theoretically eligible for participation, participants are asked to give informed consent in writing prior to any clinical study specific procedures. Both the participant and investigator (or designee) will sign and date the informed consent form. After, a liver biopsy will be performed (unless there is a liver biopsy available obtained < 32 weeks prior to screening) to assess if histopathological inclusion criteria are met.
A MRI of the liver and surrounding area (including pancreas and visceral adipose tissue; as previously specified) will be performed for all eligible participants before the biopsy, unless the biopsy was already performed prior to inclusion.
All eligible participants will visit the study physician 4 times; at baseline, after 8, 16, and 24 week. The study visits will be 8 weeks apart, although a margin of -3 days to +3 days is implemented to take participants schedule and availability into account.. At baseline, blood

and feces, will be collected, a Fibroscan will be made and participants will fill out the SF36.2 and CDLQ-NAFLD quality of life questionnaires. After, participants receive 21 LFMT or placebo capsules to orally ingest. The ingestion of the 21 capsules will be spread over the day to prevent clotting of capsules in the stomach. In addition, for the whole duration of the study participants will daily ingest 2 LFMT capsules (or placebo). At the next visit after 8 weeks blood will be withdrawn, feces will be collected and patients will again ingest 21 LFMT capsules (or placebo). The visit at 16 weeks will be the same as the visit at 8 weeks. The visit at 24 week will be the same as the baseline visit, however, at 24 weeks the visit will also include a liver biopsy. Moreover, patients will hand in fecal samples at baseline, week 2, 8, 10, 16, 18, and 24. Also, continuous glucose measurements will be performed at home using portable devices, during a consecutive period of 1 week (7 days) in the week before baseline, week 1, 9, 17 and 25.

Throughout the study period, the participants will, in addition to the LFMT or placebo capsules, take the 3 bacterial strains in the morning combined with FOS. They will further be asked to retain their usual dietary habits during the study and to keep a nutritional diary from week -1 to 24 to monitor caloric intake including total calories, dietary carbohydrates, fat, proteins, and fibers. Blood pressure, body weight, and changes in health status will be documented.

Interventions: From the day of the first LFMT capsules until completion of the study, all participants will receive an oral daily dose of 5 grams of fructo-oligosaccharides [Sensus, Roosendaal]. Subsequently, participants will be randomized to receive lean donor LFMT or placebo capsules. This consists of 21 capsules at the first 3 study visits, and a daily ingestion of 2 capsules during the duration of the study (24 weeks). Placebo capsules will consist of saline with a brown pigment.[1] During the 24 week study duration, all participants will also daily take 10^9 *A. soehngenii* CH-106 cells (dosage based on previous studies and safety study[2,3]; provided by Caelus Health), 10^{10} B. animalis subsp. *lactis* BLC1 (commercially available Sacco SRL) and $3x10^{10}$ pasteurized *A.muciniphila* cells (provided by A-Mansia Biotech), and 5 grams of FOS. We have previously observed that gut microbiota composition in the recipient is affected up to 8-12 weeks after donor FMT,[1] so this time window ensures a stable donor gut microbiota composition during the study. The fecal donor samples will be collected, lyophylized (‘freeze-dried’), encapsulated and stored at -80°C (see also paragraph 6.8). At home, participants will store the LFMT capsules in the freezer (-20°C). See figure 6 below.

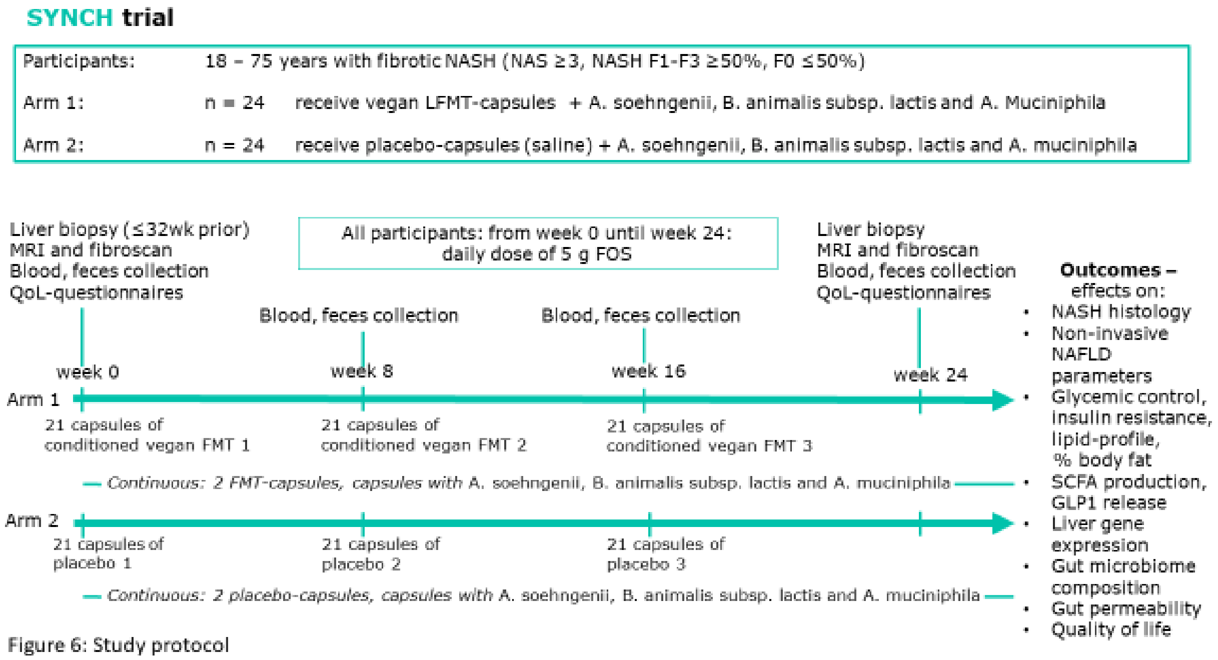

Figure 6: Study protocol

4. STUDY POPULATION

4.1 Population (base)

Recipient participants will be recruited from the multidisciplinary clinic for NAFLD-NASH Vascular Medicine/Hepatology by dr. Holleboom (location AMC) and dr. Ramsoekh (location VUmc, not part of the study staff) in Amsterdam UMC.
In order to forestall the influence of the dependent patient-physician relationship on the considerations of possible participants to enroll in the study, the following measures will be implemented. Dr. Holleboom is allowed to talk about the study with possible participants. In such a conversation, dr. Holleboom shall point out that the patient is completely free to choose to either participate or refuse the study, and that his attitude towards the patient and the care that he provides will definitely not change as a result of the choice of the patient. In addition, we implemented a mandatory period of 7 days between the provision of information and the signing of the informed consent form.

Fecal donors will be healthy, lean (BMI 18 - 25 kg/m^2), male or female individuals on a stable (>3 months) plant-based vegan diet, with negative results for infectious diseases as recommended.[1,54] Donors will be recruited by advertisements in the hospital, on social media and in local newspapers.

4.2 Inclusion criteria

Patients

- age 18-75 years
- biopsy-proven NASH obtained up to 32 weeks before screening: SAF Steatosis score ≥1, Activity ≥2, Fibrosis <4; 50% of participants should at least have NASH fibrosis stage 1, 2 or 3 according to the NASH CRN fibrosis staging system based on tandem reading of two expert liver pathologists
- fluency in Dutch or English
- participants should be able to understand the information and give informed consent

FMT donors

Donor feces will be collected from:

- Lean: BMI 18-25 kg/m2

- Age 18 to 75 years
- No protonpump-inhibitor use or use of antibiotics in 3 months prior to donation
- Adherence to vegan diet

4.3 Exclusion criteria

A potential participant who meets any of the following criteria will be excluded from participation in this study:

Patients

- Current or history of significant alcohol consumption for a period of more than 3 consecutive months within 1 year before screening (significant alcohol consumption is defined as more than 2 international units/day for females and more than 3 international units/day for males, on average; 1 international unit contains ±14 grams of alcohol)
- liver cirrhosis or hepatocellular carcinoma
- hepatitis B and/or C
- auto-immune hepatitis
- Wilson's disease
- primary sclerosing cholangitis
- primary biliary cholangitis
- alpha-1-antitripsine deficiency and hemochromatosis
- history of liver transplant, current placement on a liver transplant list
- use of pre-, pro- or synbiotics
- use of systemic antibiotics 3 month prior to randomization
- use of tamoxifen, methotrexate or amiodarone
- prior or planned bariatric surgery
- active GLP-1 receptor agonist treated diabetes mellitus
- bleeding disorder
- International normalized ratio (INR) of prothrombin time >1.4 or platelet count <100 10^9/L at screening
- anti-platelet/coagulant therapy use which cannot be temporarily discontinued
- any major cardiovascular event within 6 months prior to screening (e.g. myocardial infarction, cerebrovascular accident)
- prolonged compromised immunity (e.g. recent cytotoxic chemotherapy, HIV-infection with a CD4 count < 240)

- active or prior history of invasive malignancy (except for curatively treated in situ carcinomas [e.g., cervix] or non-melanoma skin cancer) unless a complete remission was achieved
- surgery scheduled for the trial duration period, except for minor surgical procedures, in the opinion of the investigator
- pregnant or nursing women
- any condition which, in the investigator's opinion, might jeopardize participants' safety or compliance with the protocol
- participation in another concomitant clinical trial.

FMT donors

FMT donors with one of the following will be excluded, screening will be performed at the first visit, once after 1-2 months, and thereafter every 3 months (as recommended by 'fecal transplantation guidelines'). The screening will be executed following a a step by step approach. First, potential donors will receive the information letter. The informed consent will be executed via telephone, wereafter potential donors sign the informed consent forms and send via mail to the researcher. When potential donors are eligible and informed consent is obtained, potential donors will receive a questionnaire. Depending on eligibility as assessed with the questionnaire, potential donors will be screened for parasites using a double feces test (DFT). When potential donors pass this screening, the rest of the screening, consisting of further fecal analysis and a blood withdrawal, will be completed. In addition, not for screening ends, but for investigational purposes (to assess the composition of donors gut microbiome , the last step of the screening of the potential donors will also included a 16s-sequencing to analyse the gut microbiome of potential donors. The parameters that the screening include are:

- Unsafe sex practice and other factors that imply a high risk of acquiring infectious diseases (questionnaire)
- Any medication use including PPI and antibiotics in the past 3 months
- Serological presence HIV, hepatitis A, B and/or C, active cytomegalovirus (CMV), active Epstein-Barr virus (EBV), lues, amoebiasis or strongyloides
- Presence of fecal bacterial pathogens (Salmonella, Shigella, Campylobacter, Yersinia, enteropathogenic E. coli), transmittable viruses (Rotavirus, Norovirus, enterovirus, parechovirus, sapovirus, adenovirus 40/41/52, astrovirus), parasites, multidrug resistant bacteria (MDR), extended spectrum beta-lactamase producing bacteria (ESBL).

- Positive C. difficile stool test
- Individuals with an increased risk for one of the above conditions (e.g. high-risk sexual contacts, recent blood transfusions) will be excluded.

4.4 Sample size calculation

Based on our FMT pilot study [27] and the study of *A. soehngenii* in MetSy (average GLP1 increase: 42%), taking into account the additional potential benefits of the synbiotic treatment approach, we performed a power analysis to calculate the number of participants necessary to detect a 25% reduction in our primary outcome parameter, reversal of steatosis and necro-inflammation following donor FMT combined with the synbiotic treatment. For a desired alpha of 0.05 and a desired power of 0.8, a sample size of 24 participants/arm will be required, ergo 48 total.

5. TREATMENT OF SUBJECTS

5.1 Investigational product/treatment

Allogenic Lyophilized Faecal Microbiota Transplantation (LFMT) capsules
Fecal microbiota of vegan donors will be lyophilized, encapsulated in stomach acid resistant capsules (next generation enteric capsules; NGE capsules), and stored in -80°C (for an in depth description, see paragraph 6.8). Participants will ingest 21 capsules at the first 3 study visits (baseline, 8 weeks, 16 weeks), and will daily ingest 2 capsules. FMT of vegan donor contains a large amount of butyrogenic bacteria. FMT in itself is a safe therapeutic, and FMT administered via nasoduodenal tube showed no (serious) adverse events in our pilot study or other studies of this group (> 500 performed). Moreover, studies investigating LFMT capsules did not report an increase of any adverse events (see also paragraph 6.4).
The LFMT capsules will be produced in the Amsterdam UMC, location AMC, under supervision of our pharmacist K. Wortelboer, who has experience in the research and production of LFMT capsules.

A. soehngenii CH106
A. soehngenii CH106 is a Gram-positive, catalase negative bacterium belonging to the clostridial cluster XIVa of the phylum Firmicutes and is a normal inhabitant of the human intestinal tract. *A. soehngenii* CH106 was produced at NIZO according to Hazard Analysis and Critical Control Points (HACCP) standards (see product dossier). After growth cells were harvested, washed and lyophilized in the presence of sucrose, maltodextrin, proline and NaCl. The freeze-dried powder was supplemented with maltodextrin, magnesium stearate and silicium oxide and capsulated in NGE caps to a final concentration of >$1x10^9$ cells per capsule.

B. animalis subsp. lactis BLC1
B. animalis subsp. *lactis* BLC1 is a Gram-positive, rod-shaped bacterium belonging to the Bifidobacterium genus of the phylum Actinobacteria and is commonly found in the human intestinal tract.
B. animalis subsp. *lactis* BLC1 was produced at SACCO Srl. in Italy according to Hazard Analysis and Critical Control Points (HACCP) standards and was obtained as a commercial product from SynBiotec (SynBiotec S.r.l., Via Gentile III da Varano 62032 Camerino (MC), Italy).

Pasteurized A. muciniphila MucT

Pasteurized *A. muciniphila* MucT (ATCC BAA-835^{T}), abbreviated to pAkk, is a gram-negative anaerobic bacterium belonging to the phylum Verrucomicrobia. It is a 'commensal' bacterium, meaning that it is naturally found in the gastrointestinal tract of humans and animals in relatively significant quantities. In fact, it represents between 0.5 and 5% of all the bacteria colonizing the intestine. Its production in line with good manufacturing practice (GMP) and HACCP consists of an anaerobic fermentation followed by pasteurisation and concentration of the bacterial cells. The cells are then mixed with cryoprotectants and freeze-dried in order to produce a powder.

5.2 Use of co-intervention

In addition to the FMT and probiotics, participants will receive FOS. This is discussed in the following chapters.

5.3 Escape medication

Not applicable

6. INVESTIGATIONAL PRODUCT

6.1.1 Investigational product name and formulation

Allogenic Lyophilized Faecal Microbiota Transplantation (LFMT) capsules

6.1.2 Other product name(s) and formulation(s)

Anaerobutyricum soehngenii CH106

- Product name: *Anaerobutyricum soehngenii* CH106*,* ≥ $1x10^9$ live bacterial cells (per capsule).
- Product Sponsor: Caelus Health
- Product Manufacturer: NIZO
- Active compound: *Anaerobutyricum soehngenii* CH106
- Formulation: active compound and sucrose, dextrinized corn-starch, magnesium stearate, silica dioxide and NaCl
- One capsule to be ingested once daily before breakfast, with a glass of water
- If the participant misses a dose in the morning, they can continue to take the missed dose later in the day (before meal). Participants should resume the normal instruction on the following day and missed doses should not be moved to the following day.

Bifidobacterium animalis subsp. Lactis BLC1

- Product name: *Bifidobacterium animalis* subsp. *Lactis BLC1,* ≥ $1x10^{10}$ live bacterial cells (per capsule).
- Product Sponsor: Caelus Health
- Product Manufacturer: SACCO Srl
- Active compound: *Bifidobacterium animalis subsp. Lactis BLC1*
- Formulation: information will follow upon delivery (as it will be stated in the certificate of analysis)
- One capsule to be ingested once daily before breakfast, with a glass of water.
- If the participant misses a dose in the morning, they can continue to take the missed dose later in the day (before meal). Participants should resume the normal instruction on the following day and missed doses should not be moved to the following day.

Pasteurized Akkermansia muciniphila MucT

- Product name: pasteurized *Akkermansia muciniphila MucT,* around $3x10^{10}$ pasteurized bacterial cells (per capsule).
- Product Sponsor: A-Mansia Biotech
- Product Manufacturer: SACCO Srl
- Active compound: *pasteurized Akkermansia muciniphila MucT*
- Formulation: active compound and food excipients authorized for use in food for human consumption, being microcrystalline cellulose (E460), magnesium stearate (E470b), Talcum powder (E553b) and hydroxypropylmethylcellulose caps.
- One capsule to be ingested once daily 15 minutes before breakfast, with a glass of water.

- If the participant misses a dose in the morning, they can continue to take the missed dose later in the day (before meal). Participants should resume the normal instruction on the following day and missed doses should not be moved to the following day.

Fructo-oligosaccharides

- Product name: Frutalose® OFP
- Product Sponsor: Sensus BV provides the oligofructose as a gift to UMCA.
- Product Manufacturer: Sensus BV and co-packer (for tubs/ sachets)
- Active compound: oligofructose (fructo-oligosaccharides or oligo-saccharides)
- Formulation: Frutalose® OFP contains 92 ± 2% oligofructose. Oligofructose is a polydisperse mixture of linear fructose polymers partly ended by a glucose molecule, coupled by means of β(2-1) bonds; it comprises a polydisperse mixture of linear fructose oligomers with degree of polymerization ranging between 2 and 10 units.
- Powder of 5 g to be ingested at same time daily after sprinkling on or mixed in foods (e.g. breakfast cereal/ porridge/ yoghurt) or drinks (e.g. coffee/ tea). Fresh orange juice is possible too provided immediately consumed after mixing. Soft drinks are not suitable due to very low pH. Powder will be delivered in sachets.
- If the participant misses a dose in the morning, they can continue to take the missed dose later in the day (before meal). Participants should resume the normal instruction on the following day and missed doses should not be moved to the following day.

6.2 Name and description of investigational product(s)

Participants will be treated with either allogenic LFMT capsules of a vegan donor or capsules with saline placebo with brown pigment (NaCl 0.9%). Stool of lean vegan donors is collected, lyophilized (freeze-dried), encapsulated in NGE capsules, and stored in -80°C (see paragraph 6.8). The day prior to the first study visit, participants will ingest laxatives (macrogol/kleanprep) to enhance engraftment. At the study visits at baseline, week 8 and 16, participants ingest 21 LFMT-capsules. All other days of the 24 week study period, participants ingest 2 LFMT capsules.

Daily, participants will have to take one capsule of Anaerobutyricum soehngenii CH106 (≥ $1x10^9$ live bacterial cells), one capsule of Bifidobacterium animalis subsp. Lactis BLC1 (≥ $1x10^{10}$ live bacterial cells), one capsule of pasteurized *Akkermansia muciniphila* MucT (≥ $3x10^{10}$ pasteurized bacterial cells), and 5 grams of fructo-oligosaccharides.

6.3 Summary of findings from non-clinical studies

Anaerobutyricum soehngenii

In obese mice, *A. soehngenii* treatment improved insulin sensitivity and reduced intrahepatic triglyceride content.[28] *A. soehngenii*[29] is well known to not only convert sugars but also lactate and acetate into butyrate. This latter SCFA may protect against NASH.[23,28] Butyrate may reduce the inflammatory component of NAFLD by promoting differentiation

of regulatory T cells.[30] It also has an anti-migratory effects on neutrophils[31], and in the gut, it protects epithelial barrier integrity[32], both potentially protective effects on NASH. Moreover, lactate is found to be increased in T2DM; the efficient conversion of lactate into butyrate by *A. soehngenii* is expected to contribute to its beneficial actions.

Bifidobacterium animalis, subsp. lactis

In this context, the role of *B.animalis* subsp. *lactis* is important to note as the well-studied strain BLC1 is marketed as a probiotic and well characterized.[33] Via cross-feeding it can form a trophic chain with *A. soehngenii.* Fermenting FOS*, B.animalis* subsp. *lactis* can produce lactate and acetate, which *A. soehngenii* uses to produce butyrate. This is, moreover, shown in in vitro studies of a model of the small intestine (Deyaert et al 2022, in preparation).

Akkermansia muciniphila

Pasteurized *A. muciniphila* is involved in host immunological homeostasis at the gut mucosa, improvement of gut barrier function and contributes to metabolic health. In a study assessing the effects of pasteurized *A. muciniphila* on diet-induced metabolic disorders in mice, it was not only found that pasteurization did not diminish the beneficial effects, but it also unexpectedly enhanced the beneficial impact of *A. muciniphila.*[35] A recent study in a high fat induced fatty liver mouse model showed that administration of *A. muciniphila* cells prevented fatty liver disease by regulation of the expression of genes that regulate fat synthesis and inflammation in the liver.[38] In both earlier mice and human studies it was found that pasteurized *A. muciniphila* cells were similar or more efficient than the same amount of live *A. muciniphila* cells.

6.4 Summary of findings from clinical studies

Allogenic Lyophilized Fecal Microbiota Transplantation (LFMT) capsules

We recently published the first vegan fecal microbiota transplantation study in patients with NASH and aberrant microbial composition.[27] This pilot study found anti-inflammatory effects of vegan FMT into NASH patients, with a trend towards reduced hepatic necro-inflammation. Although the exact mechanism is not well understood, the key hypothesis is that the microbiota of vegan donors consist of more butyrate producers and less gram negative lactate producers. By transplanting (and thus replacing) the microbiota, more butyrate (and less lactate) is produced in the intestines.

The current study will use LFMT capsules instead of (more traditional) fresh FMT administered per nasoduodenal tube, The first notion of LFMT capsules stems from 2015,

when Tian et al. effectively treated 1 rCDI patient with 5 LFMT capsules for two consecutive days.[55] Next, Hecker et al. treated 20 rCDI patients with LFMT capsules, which resulted in successful resolution of diarrhea in 17 (85%) patients.[56] These findings were reproduced three other prospective cohort studies reporting successful resolution of CDI in 43/49 (88%), 28/32 (88%) and 33/37 (89%) of patients.[57–59] The first RCT in which LFMT capsules were compared to frozen FMT via enema as treatment for rCDI was conducted in 2018 by Jiang et al.[60] This study showed an equal efficacy for both the LFMT and frozen FMT products, with 26/31 (84%) and 30/34% (88%) of patients achieving resolution of rCDI, respectively. In addition, authors did not find any differences in adverse events between both groups. Finally, one RCT has been published comparing LFMT capsules to placebo for treatment of small intestinal bacterial overgrowth.[61] After taking LFMT capsules once a week for four consecutive weeks, gastrointestinal symptoms significantly improved and gut microbiota diversity significantly increased compared to the placebo group.

In addition to a comparable efficacy of LFMT capsules with fresh FMT, above studies showed oral treatment with capsules is relatively simple, well tolerated and safe. Adverse events were limited to minor gastrointestinal symptoms and comparable to fresh FMT.[57–60] The most commonly reported adverse experiences are gastrointestinal symptoms, including abdominal pain, fecal urgency, nausea, diarrhoea and flatulence.[60] In addition, like fresh FMT, the lyophilized microbiota was able to increase bacterial diversity and shift the microbiota composition towards a donor profile, which was observed up to one year following LFMT treatment.[57,58]

Besides above-mentioned studies with LFMT capsules, multiple studies have been performed with capsules containing a frozen FMT suspension. Among these, FMT capsules have been used to treat irritable bowel syndrome[62], hepatic encephalopathy[63], metabolic syndrome[64] and obesity[65]. Finally, capsules containing a frozen autologous fecal suspension have been tested as a supplement to successfully prevent weight regain after a lifestyle intervention.[66] Like the LFMT capsules, these capsules were found to be well tolerated, safe and effective. Although the formulation and processing is different within most studies, these studies show that the fecal transplant remains viable and able positively influence the recipients’ intestinal microbiome.

Anaerobutyricum soehngenii

The pilot study also showed that upon vegan FMT engraftment, the gut microbiome of the NASH patients was enriched in *Anaerobutyricum soehngenii* and other butyrate-producing Lachnospiracae.[27] Furthermore, in obese mice, *A. soehngenii* treatment improved insulin sensitivity and reduced intrahepatic triglyceride content.[28] *A. soehngenii*[29] is well known to

not only convert sugars but also lactate and acetate into butyrate. As previously stated, this latter short chain fatty acid may protect against NASH.[23,28] Butyrate may reduce the inflammatory component of NAFLD by promoting differentiation of regulatory T cells.[30] It also has an anti-migratory effects on neutrophils[31], and in the gut, it protects epithelial barrier integrity[32], both potentially protective effects on NASH. Moreover, lactate is found to be increased in T2DM; the efficient conversion of lactate into butyrate by *A. soehngenii* is expected to contribute to its beneficial actions.

Bifidobacterium animalis subsp. *lactis BLC1*
Bacterium *Bifidobacterium animalis* subsp. *lactis* BB12 is arguably the most studied probiotic bacterium.[67] It's potential role in many conditions, varying from dental caries[68] to infant colic[69] and metabolic syndrome[70] has been studied. Some studies also addressed its potential role application in NAFLD.[71,72] The majority of these studies involved the use of additional bacteria.
The strain that is used for the SYNCH study is *Bifidobacterium animalis* subsp. *lactis* BLC1, the closest relative of BB12, showing 99,9% sequence identity at the nucleotide level.[73]

Akkermansia muciniphila
A randomized, double-blind, placebo-controlled proof-of-concept study in overweight/obese insulin-resistant human volunteers showed that the daily supplementation with pasteurized *A. muciniphila* for 12 weeks improved several metabolic parameters such as insulin sensitivity, insulinemia, plasma total cholesterol, as well as relevant blood markers for liver dysfunction and inflammation while serum lipopolysaccharide levels were decreased, indicative of improved barrier function.[37]

6.5 Summary of known and potential risks and benefits

Allogenic Lyophilized Fecal Microbiota Transplantation (LFMT) capsules
FMT is a well-studied and safe procedure.[74,75] Previous studies with LFMT capsules have indicated no procedure-related serious adverse events after treatment with LFMT capsules and only minor gastrointestinal symptoms were reported by participants. These gastrointestinal symptoms consisted mainly of abdominal pain, fecal urgency, nausea, diarrhoea and flatulence, and were similar in the placebo group.[60]
One potential adverse event with FMT delivered via the upper gastrointestinal route, is the risk of FMT reflux into the stomach. To date, no such events have been reported after ingestion of LFMT capsules. To mitigate this risk, the LFMT is encapsulated in NGE

capsules, which will not disintegrate until they have passed the stomach. In addition, the ingestion of the bulk of 21 capsules is spread evenly over the day, so that the capsules will not clot together in the stomach (impairing passage) and reducing the chance of nausea and vomiting. In comparison, the daily ingestion of 25 capsules for 3 consecutive days was well tolerated in a study with irritable bowel patients, without serious adverse events.[62]

Pre- and probiotics

Anaerobutyricum soehngenii and *Bifidobacterium animalis,* subsp*. lactis* are commensal inhabitants of the human intestine, and safe to administer. Pasteurized *A. muciniphila* is a pasteurized form of another commensal of the gut, and safe to administer in humans.[76] The combination of these 3 bacteria is not well studied, although we expect no negative effect of interbacterial actions.

Fructo-oligosaccharides are indigestible prebiotics, which are safe and over-the-counter available.

6.6 Description and justification of route of administration and dosage

FMT or placebo will be given per orally per capsule. All bacterial strains and FOS will be ingested orally per capsule. The dosage of 10^9 *A. soehngenii* CH-106 cells is based on previous studies and safety study[2,3] (provided by Caelus Health). The dosage of 10^{10} B. animalis subsp. *lactis* is widely recognized and used.[77,78] The dosage of $3x10^{10}$ pasteurized *A.muciniphila* cells is based on safety and efficacy studies (provided by A-Mansia Biotech).

6.7 Dosages, dosage modifications and method of administration

Allogenic Lyophilized Fecal Microbiota Transplantation (LFMT) capsules

One LFMT capsule will contain approximately 235mg of lyophilized feces. This equals the bacterial load of approximately 750mg fresh feces, or 1500mg of fresh FMT. Depending on the fecal matrix and the water content, this might be slightly more or less. The dosage of the capsules will consist of 3 times a loading dose, and a maintenance dose. The loading dose will be 21 capsules on 1 day, ingested at the study visits. The maintenance dose will consist of the ingestion of 2 capsules daily for 24 weeks. The loading doses and the maintenance dose together equal the amount of 100 grams of fresh FMT administered every 8 weeks.

Capsules are to be ingested with a glass of water on an empty stomach in the morning, preferably an hour before breakfast (while still fasted). Patients store the LFMT capsules at home in a freezer, but can let the capsules warm to room temperature before ingestion.

Pre- and Probiotics

Anaerobutyricum soehngenii CH106 is given orally per capsule in a dosage of approximately ≥ $1x10^9$ live bacterial cells. Bifidobacterium animalis subsp. Lactis BLC1 is given orally per capsule in a dosage of around ≥ $1x10^{10}$ live bacterial cells. Pasteurized *Akkermansia muciniphila* MucT is given orally per capsule in a dosage of around $3x10^{10}$ pasteurized bacterial cells. Fructo-oligosaccharides are given orally as a powder in a dosage of 5 grams, once a day.

6.8 Preparation and labelling of Investigational Medicinal Product

Allogenic Lyophilized Fecal Microbiota Transplantation (LFMT) capsules

Feces donors deliver their fresh feces cooled to the investigator at the AMC. The autologous feces are directly processed, whereby the feces are homogenized 1:1 with a lyoprotectant solution (10% trehalose and 5% maltodextrin in sterile saline, all pharmaceutical grade). Thereafter, the fecal suspension is filtered through double non-woven gauzes in a metal funnel (sterile) to remove any particulate matter. The resulting FMT is transferred to sterile flasks, 50 ml/flask, and frozen at -80°C. Next, the flasks with frozen FMT are placed in the drying chamber of a freeze-dryer (Zirbus VaCo 2) and lyophilized during 48 hours. The resulting lyophilized FMT is subsequently transferred to a mortar, ground to a fine powder and mixed with lubricant (silicon dioxide, pharmaceutical grade) to improve the flowing properties of the powder. If necessary, filler is added to the powder mixture (microcrystalline cellulose, pharmaceutical grade) to reach the necessary volume for the number of capsules to be filled. Finally, the LFMT powder is encapsulated in capsules with stomach acid resistant properties (NGE-caps, Capsugel). LFMT capsules are filled in opaque HDPE 50 ml containers (DUMA Twist-off), 28 capsules per container. Containers are labelled according to GMP-annex 13 and stored in a freezer at -80°C.

The image below shows a schematic overview of the preparation to the LFMT capsules.

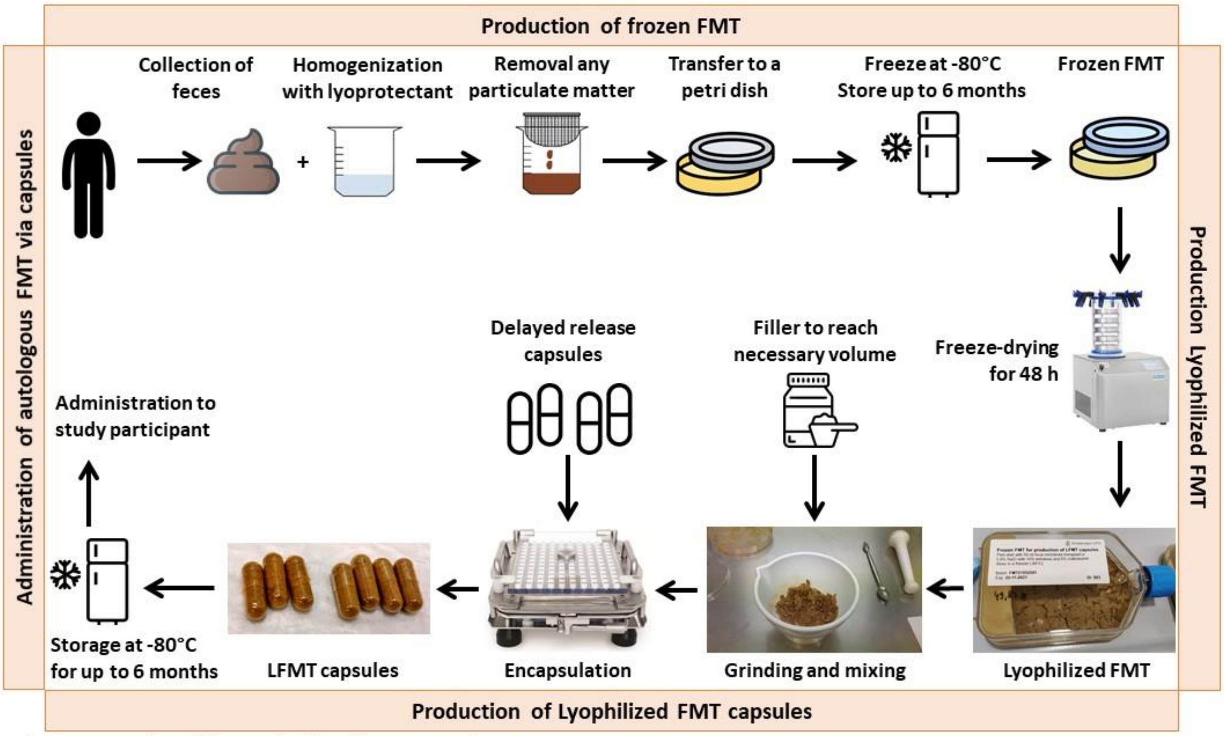

Figure 7: Overview of the production of LFMT capsules.

<u>The label of the LFMT capsules will contain:</u>

- the name of the study: the SYNCH-trial
- the Participant Number
- the Sponsor name
- the investigators name and contact details
- For investigational use only
- LFM-capsules.
- Contains: Bevat freezedried feces, natriumchloride, trehalose, maltodextrin and colloidal silicium dioxide.
- the Batch Number and the Expiry Date
- Storage conditions: Store at room temperature (-20 to -4°C), in a dry place away from direct sunlight – Keep away from children
- Instructions for use: Consume two caps per day, 15 minutes before breakfast with a glass of water.
- Emergency Contact Number
- Amsterdam UMCs address.

All labels of the bacterial strains and the FOS will contain:

- the name of the study: the SYNCH-trial
- the Participant Number
- the Sponsor name
- the investigators name and contact details
- For investigational use only
- Type of probiotic
- the Batch Number and the Expiry Date
- Storage conditions: Store at room temperature (15-25°C), in a dry place away from direct sunlight – Keep away from children
- Instructions for use: Consume one caps per day, 15 minutes before breakfast with a glass of water
- Emergency Contact Number
- Company name of the supplier.

6.9 Drug accountability

Fecal microbiota transplantation

Excipients for the LFMT capsules (trehalose, maltodextrin, sterile saline, silicon dioxide and microcrystalline cellulose, all pharmaceutical grade) will be ordered and handled by the Amsterdam UMC Hospital Pharmacy under supervision of Dr. Marleen Kemper, hospital pharmacist at the Amsterdam UMC. In addition, the stomach acid resistant capsules, NGE-caps, will be ordered at Capsugel via the Hospital Pharmacy. Containers will be ordered via the Amsterdam UMC Hospital Pharmacy as well. All excipients and materials will be stored in a dedicated production room for the LFMT capsules at a dry place below 25°C according to prescription by the manufacturer.

The executing researchers will be responsible for the production, storage and dispensing of the LFMT capsules to the respective study subjects, as well as the drug accountability. At the end of the intervention period, any remaining LFMT capsules will be returned by the study subjects to the investigator and will be counted and destroyed by the executive researcher.

Anaerobutyricum soehngenii CH106

Capsulated formulation of *A. soehngenii* CH106 will be delivered by Caelus in small bottles, each containing 50 capsules and a small silicabag to ensure low moisture inside the bottles. The sponsor will be responsible for storing *Anaerobutyricum soehngenii* CH106
in a proper place, inaccessible to unauthorized persons. The sponsor will be responsible for storing pAkk at the appropriate conditions and inaccessible to unauthorized persons.

The investigator agrees that *Anaerobutyricum soehngenii* CH106 will be dispensed only to study participants who have provided written consent. Participants will be instructed to store the product at room temperature (15-25°C), in a dry place away from direct sunlight.

Bifidobacterium animalis subsp. lactis BLC1

Capsulated formulation of *B. animalis* subsp. *lactis* BLC1, produced by SynBiotec will be delivered by Caelus. Further details can be provided upon delivery.
The sponsor will be responsible for storing *Bifidobacterium animalis subsp. lactis BLC1* in a proper place, inaccessible to unauthorized persons. The sponsor will be responsible for storing pAkk at the appropriate conditions and inaccessible to unauthorized persons.
The investigator agrees that *Bifidobacterium animalis subsp. lactis BLC1* will be dispensed only to study participants who have provided written consent. Participants will be instructed to store the product at room temperature (15-25°C), in a dry place away from direct sunlight.

Pasteurized Akkermansia muciniphila MucT

Pasteurized Akkermansia muciniphila will be provided to the sponsor by A-Mansia biotech. Production, packaging and labeling of pAkk will be performed by A-Mansia Biotech.
The sponsor will be responsible for storing pAkk in a proper place, inaccessible to unauthorized persons. The sponsor will be responsible for storing pAkk at the appropriate conditions and inaccessible to unauthorized persons.
The investigator agrees that pAkk will be dispensed only to study participants who have provided written consent. Participants will be instructed to store the product at room temperature (15-25°C), in a dry place away from direct sunlight.

7. NON-INVESTIGATIONAL PRODUCT

7.1 Name and description of non-investigational product(s)

Fructo-oligosaccharides

FOS belong to the group of inulin-type fructans (ITFs); water soluble, non-digestible and fermentable carbohydrates. A lot of vegetables contain inulin-like fructans, such as chicory roots, asparagus, onions, bananas and garlic.

Theses ITFs are β-(2-1)-linked fructans with a terminal α-linked glucose. Fructo-oligosaccharides have a chain length of <10.

ITF cannot be digested by humans due to the aforementioned bonds, but can however be fermented by bacteria, such as *Bifidobacterium animalis* subsp. *lactis* and other bifidobacterial species within the human colonic microbiota..

7.2 Summary of findings from non-clinical studies

Fructo-oligosaccharides are safe and extensively studied in humans in dosages ≤ 20 grams per day. Moreover, it is part of a healthy diet. Therefore, we will only discuss the findings from clinical studies.

7.3 Summary of findings from clinical studies

One approach of modifying gut microbiota in adults is by supplementation with fructo-oligosaccharides (FOS), which are also prebiotics. The most recent definition of prebiotics by the International Scientific Association for Pro- and Prebiotics (ISAPP) is “a substrate that is selectively utilized by the host microorganisms conferring a health benefit”.[79] FOS have been recognized as prebiotic by the ISAPP in this recent publication. The European Union (Reg EC No 1169/2011) definition comprises “carbohydrate polymers with three or more monomeric units, which are neither digested nor absorbed in the human small intestine” and again categories are defined. It is noteworthy that FOS prebiotics selectively stimulate certain microbiota species, while not all dietary fibres show prebiotic properties. FOS, besides being prebiotic, is also a dietary fibre.

As previously stated, FOS is safe and widely used by the food industry globally to enhance fibre in foods. It has been extensively studied in humans in dosages ≤ 20 grams a day whereby addition of FOS increased the abundance of *Bifidobacterium* in the gut microbiota.[80] The increase of *Bifidobacterium* seems dose-dependent, and significantly increasing from doses of 2.5-5 grams per day. In addition, a Belgium study found that liver histology improved in NAFLD-patients when treated with FOS.[81] Further data on ITFs in NAFLD-

patients is predominantly researched in the combination with probiotics, and in combination indirectly shows attenuation of contributing factors of NAFLD.[82–84]
The adverse effects of fructo-oligosaccharides are in decreasing order excess flatus, bloating, borborygmi, and in minor extent, mild abdominal pain. Although smaller studies describe a dose-dependent effect, with dosages above 5 grams a day increasing frequency and intensity of symptoms, this is not seen in larger sample sizes.[85–88] Serious adverse events are not reported.

7.4 Description and justification of route of administration and dosage

Inasmuch as FOS has to be fermented in the intestines, we will give it orally. It will be delivered in sachets (or tubs with scoops), which participants can dissolve in their (non-acidic) drinks, such as water, tea or coffee or sprinkled or mixed in foods such as breakfast cereal, porridge or yoghurt.

7.5 Dosages, dosage modifications and method of administration

We will give 5 grams of FOS (powder) once per day in the morning, to be taken in orally (can be easily dissolved in liquid as highly soluble or mixed in foods).

7.6 Preparation and labelling of Non Investigational Medicinal Product

The Frutalose® OFP powder will be supplied by Sensus, Roosendaal, in a 40 or 60 kg bag with a Certificate of Analysis to UAMC or the co-packer of choice for packing into sachets or tubs with scoops. Information will be provided by Sensus to facilitate the packing and labelling process as required.

- Product name: Frutalose® OFP
- Product Sponsor: Sensus BV provides the oligofructose as a gift to UMCA.
- Product Manufacturer: Sensus BV and co-packer (for tubs/ sachets)
- Active compound: oligofructose (fructo-oligosaccharides or oligo-saccharides)
- Formulation: Frutalose® OFP contains 92 ± 2% oligofructose. Oligofructose is a polydisperse mixture of linear fructose polymers partly ended by a glucose molecule, coupled by means of β(2-1) bonds; it comprises a polydisperse mixture of linear fructose oligomers with degree of polymerization ranging between 2 and 10 units.
- Powder of 5 g sachet to be ingested at same time daily after sprinkling on or mixed in foods (e.g. breakfast cereal/ porridge/ yoghurt) or drinks (e.g. coffee/ tea). Fresh orange juice is possible too provided immediately consumed after mixing. Soft drinks are not suitable due to very low pH. Powder will be delivered in sachets or in containers with a 5 gram scoop.

- If the participant misses a dose in the morning, they can continue to take the missed dose later in the day (before meal). Participants should resume the normal instruction on the following day and missed doses should not be moved to the following day.

7.7 Drug accountability

Study product Frutalose® OFP will be stored in a secure area with restricted access. The investigator agrees that the study product will be dispensed only to study subjects who have provided written consent. Compliance will be monitored by return of unused study product. Study participants will be instructed to store the sachets (or tubs with lids tightly shut) at room temperature away from moisture and direct sunlight. At the end of the study for each study participant, left over study products will be collected by the researchers for compliance and for destruction.

8. METHODS

8.1 Study parameters/endpoints

8.1.1 Main study parameter/endpoint

8.1.2 Primary outcome

To demonstrate that *A. soehngenii* combined with pasteurized *A. muciniphila* and *B. animalis* subsp. *lactis*, FOS and conditioned vegan FMT reduces NASH as defined by an improvement of liver histology in individuals with NASH and fibrosis stage 0-3, with improvement defined as reduction of steatohepatitis by ≥1 SAF-A point and no worsening of liver fibrosis, or improvement in ≥ 1 stage liver fibrosis and no worsening of steatohepatitis.

8.1.3 Secondary outcomes

Secondary outcomes

To demonstrate that the combined treatment improves:

- non-invasive outcomes of NAFLD, i.e. multiparametric MRI of liver and surrounding subcutaneous adipose tissue (MRI-PDFF, MR elastography, corrected T1), FibroScan Elastography and Controlled Attenuation Parameters, and plasma panel Enhanced Liver Fibrosis (ELF) panel, pro-C3.
- change in blood markers from baseline to end of treatment, namely: liver enzymes (i.e. alanine amino transferase (ALT), aspartate amino transferase (AST), gamma glutamyl transferase (GGT), alkaline phosphatase (ALP)), inflammatory blood markers (i.e. leukocytes, monocytes, CRP, Il-1(β), Il-6, Il-11, Il-17, Il-32, TNF-α, IFN-γ, additional markers of inflammatory pathways), SCFA (i.e. propionate, butyrate, acetate), lactate, ethanol, plasma lipids (i.e. LDL, HDL, triglycerides, total cholesterol), FGF-21, adiponectin, leptin, lipopolysaccharides, zonulin, estrogen, vitamin B12, folate acid, other metabolomic and lipodomic outcomes.
- microbiome read outs (composition, engraftment, strain tracking) en metabolites, fecal SCFA-composition, and fecal albumine
- glycemic control, insulin resistance, body weight/BMI, waist circumference and percentage body fat
- MetSy criteria / %
- SCFA production and GLP1 gene and protein expression indicating GLP-1 production
- liver gene expression profile: lipogenic, inflammatory and fibrogenic pathways
- liver pathology, histopathological features, immunofluorescence and assessment of pathophysiological proteins

- NAFLD histology as assessed with a deep-learned algorithm scoring whole slide images of liver biopsies.
-
- Continuous glucose monitoring (4x 7 consecutive days)
- quality of life (general (SF36) and NAFLD/NASH-specific (CDLQ-NAFLD)).

8.1.4 Other study parameters

- Sex
- Age
- Ethnicity
- Height
- Weight
- BMI
- Waist circumference
- Percentage body fat
- Comorbidities (e.g. diabetes)
- Smoking, yes/no
- Alcohol intake
- Polypharmacy (defined as chronic use of ≥ 5 different medications)
- Daily caloric intake
- Daily fat and sugar consumption

8.2 Randomisation, blinding and treatment allocation

Double blind, 1:1 to receive synbiotic treatment in combination with LFMT capsules or placebo. Groups will be stratified by proton-pump inhibitor use, metformin use, and histopathological fibrosis score.

8.3 Study procedures

Individuals with NASH will be recruited from our outpatient clinic.

Screening liver biopsy

Percutaneous liver biopsies will be performed as a part of screening when individuals are theoretically eligible for participation, unless a liver biopsy has been performed in the previous 36 weeks. A tandem-read by two liver pathologists blinded to any other result will determine if patients will be included in the study.

After the screening is completed, participants will be telephoned with further instructions. Individuals will have a total of one screening and four study visits, and time invested will approximately be 24 hours per person.

LFMT capsules and synbiotics

Participants will visit the study center at baseline (week 0), week 8, 16, and 24. The study visits will be 8 weeks apart, although a margin of -3 days to +3 days is implemented to take participants schedule and availability into account. On the first three visits, participants will ingest 21 LFMT capsules (or placebo) containing approximately 235mg of lyophilized FMT (this equals 1500mg of fresh FMT) each. In addition, participants will ingest 2 LFMT capsules (or placebo) daily for the duration of the study (24 weeks).

From the baseline visit until the end of the study (24 weeks later), patients will daily take 5 g FOS (powder).

Starting on baseline visit, all participants will also take daily doses of 10^9 *A. soehngenii* CH-106 cells (dosage based on previous studies including toxicology study[2,3]), 10^{10} *B. animalis* subsp*. lactis* BLC1 (a well-studied strain marketed as a probiotic by Sacco SRL) and $3x10^{10}$ pasteurized *A. muciniphila* ATCC BAA-835[T] cells (dose based on EFSA approval and obtained from A-Mansia Biotech) for the whole 24 weeks. We have previously observed that gut microbiota composition in the recipient is affected up to 8-12 weeks after donor FMT[1], so this time window ensures a stable donor gut microbiota composition during the study.

Physical examination

At baseline and after 24 weeks, we will collect anthropometric data (e.g. body weight, height, waist circumference, bioelectrical impedance etc.).

Fecal samples

In addition, feces will be collected at baseline, and after 2, 8, 10, 16, 18 and 24 weeks for extensive microbiota analysis and intestinal barrier function assessment.

Blood withdrawal

Blood will be collected at baseline, 8, 16 and 24 weeks. At baseline and 24 weeks, blood will be investigated on common liver enzymes, indicators of glycemic control, lipids, and general and more NASH-specific parameters. Blood withdrawal on 8 and 16 week is for safety and in a lesser extent for study purposes (e.g. enhanced liver fibrosis-panel).

Liver biopsy (after 24 weeks)

At 24 weeks, another liver biopsy will be performed to examine the effect of the FMT. The NASH-CRN classification[4] will be assessed on H&E slides, for steatosis, inflammation and ballooning, and with a Sirius red–stained slide for evaluation of fibrosis. RNA for RNA-sequencing will be isolated. Differential gene expression will be assessed over time (baseline and 24 weeks) and by treatment allocation (vegan donor FMT versus placebo).

MRI and Fibroscan

In addition, before baseline and at 24 weeks. a multiparametric MRI of the liver and MRI of visceral and subcutaneous fat will be performed with a 3T Philips Ingenia MRI scanner. Participants will be screened for contra-indications for MRI prior to inclusion in this study. Participants will be provided with metal free hospital clothing if necessary. The total scan time will be approximately 45 minutes to estimate visceral and subcutaneous adipose tissue depot volume, hepatic and pancreatic fat content, as well as hepatic fibrosis and inflammation. In addition, a Fibroscan (ultrasound) will be performed to investigate liver stiffness.

Food diary, glucose monitoring and questionnaires

Participants will, moreover, keep a diary of their daily food intake for 5 days in the week before and after study visits. In addition, they will fill in questionnaires physical endurance and quality of life at baseline and 24 weeks.

Last, continuous glucose measurements will be performed at house using portable devices, during a consecutive period of 1 week (7 days) in the week before baseline, week 1, 9, 17 and 25.

8.4 Figure 8 summarizes the study visits.Withdrawal of individual subjects

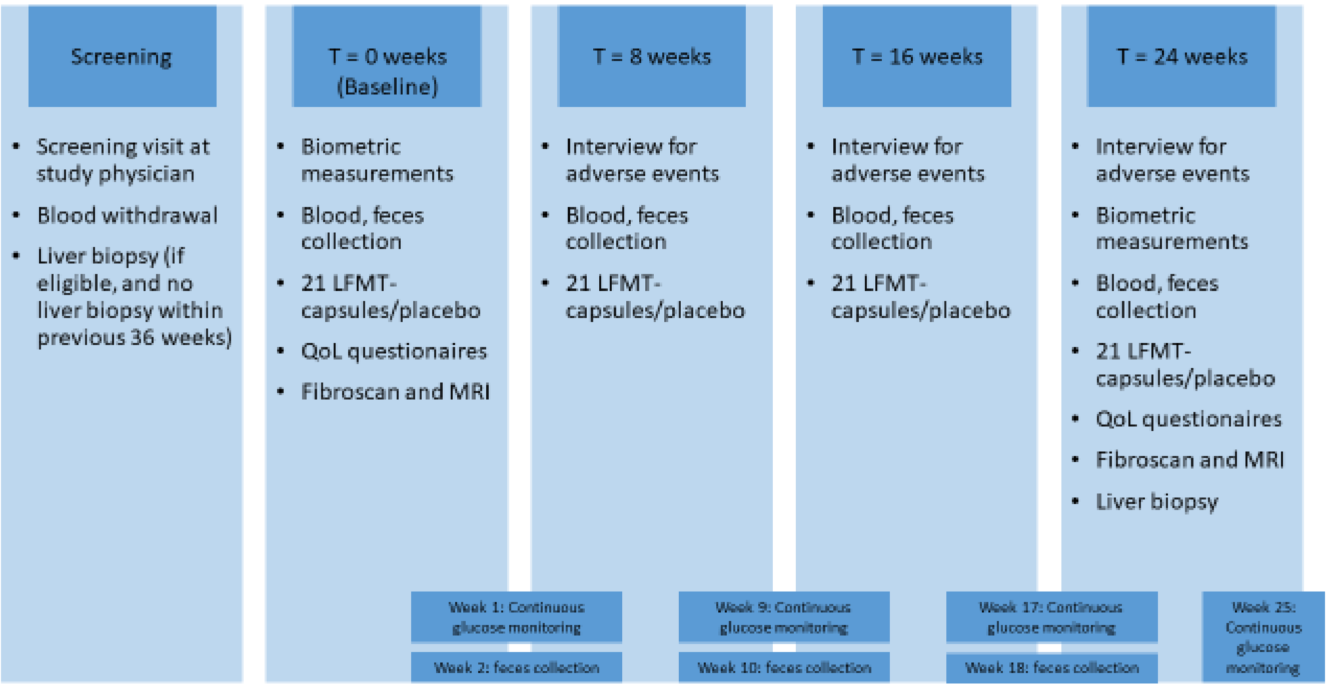

Figure 8: Overview of study visits

Participants can leave the study at any time for any reason if they wish to do so without any consequences. The investigator can decide to withdraw a participant from the study for urgent medical reasons.

8.4.1 Specific criteria for withdrawal

Participants with any new condition during the study that require hospital admission, change in drug prescriptions or likely to affect the outcome of this study will be withdrawn. Participants will also be withdrawn if instructions as written in the protocol are not followed.

8.5 Replacement of individual subjects after withdrawal

Withdrawn participants will be replaced in order to attain the desired sample size.

8.6 Follow-up of subjects withdrawn from treatment

Withdrawn patients will be followed for SAEs until 6 months after inclusion.
Participants withdrawn for medical reasons will be followed until the interfering condition has resolved or reached a stable state.

8.7 Premature termination of the study

In case of multiple SUSARS the study will be terminated.

9. SAFETY REPORTING

9.1 Temporary halt for reasons of subject safety

In accordance to section 10, subsection 4, of the WMO, the sponsor will suspend the study if there is sufficient ground that continuation of the study will jeopardize participants' health or safety. The sponsor will notify the accredited METC without undue delay of a temporary halt including the reason for such an action. The study will be suspended pending a further positive decision by the accredited METC. The investigator will take care that all participants are kept informed.

Deblinding of subjects can be done by drs. C.M. Fuhri Snethlage and drs. D. Stols-Goncalves, both researchers in the experimental vascular medicine department that are fully independent of this study.

9.2 AEs, and SAEs

9.2.1 Adverse events (AEs)

Adverse events are defined as any undesirable experience occurring to a individual during the study, whether or not considered related to [the investigational product / trial procedure/ the experimental intervention].

An event that is part of the natural course of the disease under study is captured in the study as a clinical activity measure. However, any specific symptoms or sequelae of the disease evolution (e.g., organ failure, respiratory distress) will be considered AEs. Clinically significant changes in laboratory values, blood pressure and pulse will not necessarily be reported as AEs. However, abnormal AEs that constitute an SAE or lead to discontinuation of administration of study drug must be reported and recorded as an AE.

The investigator is responsible for reviewing laboratory test results and determining whether an abnormal value in an individual study subject represents a change from values before the study. In general, abnormal laboratory findings without clinical significance (based on the Investigator's judgment) should not be recorded as AEs; however, laboratory value changes requiring therapy or adjustment in prior therapy should be considered AEs.

9.2.2 Serious adverse events (SAEs)

A serious adverse event is any untoward medical occurrence or effect that

- results in death;
- is life threatening (at the time of the event);
- requires hospitalisation or prolongation of existing inpatients' hospitalisation;
- results in persistent or significant disability or incapacity;

- is a congenital anomaly or birth defect; or
- any other important medical event that did not result in any of the outcomes listed above due to medical or surgical intervention but could have been based upon appropriate judgement by the investigator.

An elective hospital admission will not be considered as a serious adverse event.
A hospitalization meeting the regulatory definition for “serious” is any inpatient admission, regardless of the length of stay, even if as a precautionary measure for continued observation. Elective hospitalizations for the routine administration of protocol therapy, therapeutic blood products, treatment of a pre-existing condition that did not worsen from baseline, elective procedures, or for social or technical reasons are not considered SAEs. However, prolongation of hospitalization or re-admission after the individual has been discharged for reasons other than administrative, will be considered SAEs.

All SAEs that occur after any participant has been enrolled (i.e. who signed ICF), before treatment, during treatment, or within 30 days following the cessation of treatment, whether or not they are related to the study, must be recorded.

The investigator will report all SAEs to the sponsor without undue delay after obtaining knowledge of the events, except for the following SAEs:

The sponsor will report the SAEs through the web portal *ToetsingOnline* to the accredited METC that approved the protocol, within 7 days of first knowledge for SAEs that result in death or are life threatening followed by a period of maximum of 8 days to complete the initial preliminary report. All other SAEs will be reported within a period of maximum 15 days after the sponsor has first knowledge of the serious adverse events.

9.2.3 Recording and Reporting of Adverse Events

AE reporting and data management will be performed according to the following relevant guidelines:

- Directive 2001/20/EC (European Clinical Trial Directive)
- Committee for Proprietary Medicinal Products (CPMP)/ICH/E2A/377/95 (Clinical Safety Data Management: Definition and Standards for Expedited Reporting)
- CPMP/ICH/135/95 (GCP)
- 21 Code of Federal Regulations (CFR) 312.32 (FDA).

9.2.4 Recording of AEs/SAEs

All SAEs must be reported on the SAE form in the eCRF.
All SAEs that occur after the participant has signed the ICF up to 30 days after the last administration of the last investigational product will be reported. All non-serious AEs that are possibly related to the study are collected from randomization until 30 days after the last visit (at 24 weeks). All SAEs must be reported on the SAE form in the eCRF.
NCI CTCAE (National Cancer Institute; Common Terminology Criteria for Adverse Events) grading is used in haem/onc and refers to mild (G1) moderate (G2), severe (G3), life-threatening (G4), or dead (G5); currently v5.0 is used.
Participants with an AE of Grade > 1 will be further followed until the resolution of the AE to CTCAE Grade 0-1 or until initiation of a new therapy, whichever occurs first.
Participants with an adverse drug reaction (ADR) ongoing at EAPV must be followed until the ADR resolves, becomes stable, or is considered not clinically significant by the investigator.
A Data Safety Monitoring Board (DSMB) will be established to assess safety data, monitor trial integrity and make recommendations to the Sponsor.

9.2.5 Relationship

The relationship or association of the study medication in causing or contributing to the AE will be characterized by the Investigator using the following classification and criteria:

- Possibly or probably related: A clinical event, including laboratory test abnormality, with a reasonable time sequence to study medication administration that might or might not be also explained by concurrent disease or other drugs or chemicals.
- Not related: A clinical event, including laboratory test abnormality, judged to be clearly and incontrovertibly due to extraneous causes (e.g., diseases, environment), or with a temporal relationship to study medication administration that makes a causal relationship improbable, and/or for which other drugs, chemicals, or underlying diseases provide a much more plausible explanation.

If applicable, the relationship of the AE to the study medication will be assessed by means of the question: “Is there a reasonable possibility that the event may have been caused by the investigational product?” The Investigator should respond to this question with either “Yes” or “No” separately for each study medication.

9.2.6 Severity

Severity or intensity will be assessed according to the NCI CTCAE v5.0 severity grading scale.

Grade refers to the severity of the AE. The NCI CTCAE v5.0 displays Grades 1 through 5 with unique clinical descriptions of severity for each AE based on this general guideline:

- Grade 1: Mild (awareness of sign or symptom, but easily tolerated)
- Grade 2: Moderate (discomfort sufficient to cause interference with normal activities)
- Grade 3: Severe (incapacitating, with inability to perform normal activities)
- Grade 4: Life-threatening (urgent intervention indicated)
- Grade 5: Death related to AE.

9.3 Follow-up of adverse events

All AEs will be followed until they have abated, or until a stable situation has been reached. Depending on the event, follow up may require additional tests or medical procedures as indicated, and/or referral to the general physician or a medical specialist.

SAEs need to be reported till end of study within the Netherlands, as defined in the protocol.

9.4 [Data Safety Monitoring Board (DSMB) / Safety Committee]

An independent DSMB is installed,comprising chair: dr. M.E. Tushuizen gastroenterologist-hepatologist at Leiden UMC, prof. dr. H.R. Buller, emeritus professor of internal medicine at Amsterdam UMC, location AMC and dr. B.A. Hutten, statistician and epidemiologist at Amsterdam UMC, location AMC before the trial will be initiated. All members of the DSMB do not have a conflict of interest concerning this study.

The main focus of the DSMB will be safety of the participants and compliance to the study protocol (see also the enclosed DSMB charter).

The advice(s) of the DSMB will be sent to the sponsor of the study and the chief medical officers of A-Mansia Biotech and Caelus Health. Should the sponsor of the study decide not to fully implement the advice of the DSMB, the sponsor will send the advice to the reviewing METC, including a note to substantiate why (part of) the advice of the DSMB will not be followed.

10. STATISTICAL ANALYSIS

Statistical analyses will be performed by SPSS statistical analysis software and R-studio. Normality of the data will be assessed by a Kolmogorov–Smirnov test. Data transformation will be done if the data is not normally distributed. The primary analyses of studied primary parameters will be performed between the baseline and endpoint intervention by analysis of a two-tailed one-sample t-test, or chi-square test. If the data is not normally distributed and data transformation is not possible, the Mann-Whitney test will be used. Further associations will be assessed with Pearson rank correlation test / Pearson chi-square test.

Paired categorical data will be analyzed with McNemars test. Paired continuous data will be analyzed using a paired T-test when normally distributed, and a Wilcoxon signed-rank test when not normally distributed.

Using R, we will use statistical packages that employ machine-learning algorithms to reveal which metabolites and microbiota best predict favourable response. An Elastic Net machine learning classification algorithm in combination with a stability selection procedure will be used to identify biological features that change differently between the two treatment groups. Also using R, a deep-learned algorithm scoring whole slide images of liver biopsies will be applied and analyzed.

11. ETHICAL CONSIDERATIONS

11.1 Regulation statement

This study will be conducted in accordance with the principles of the Declaration of Helsinki (latest version, October 2013) and in accordance with the regulations in the Medical Research Involving Human Subjects Act (WMO).

11.2 Recruitment and consent

Individuals will be recruited by their treating physician or by advertisements in a newspaper, the patient association or at the outpatient clinics. Nature, aims, methods, anticipated benefits and potential hazards related to the study will be fully explained to the participant prior to applying any study-related procedure by principal investigator, or sub-investigators. Each participant will be informed that his medical records may be reviewed during the course of the Study or afterwards, including review by government agencies. Each participant will be informed that his medical data will be included in a database and may be reviewed during the course of the Study or afterwards, including review by government agencies. However, only authorized personnel will review these data. Each participant will be informed that the investigator will protect any personal information not related to the study, and that individuals associated with the study are bound by the same confidentiality obligations as other health care professionals with regard to subject confidentiality.

Each participant must give written consent according to local rules after an adequate time to consider their decision. Two copies of the consent will be signed before performing any screen evaluations. The consent form will be written in a language that the participant can read and understand. The patient information letter and informed consent form should be attached as a separate document.

The investigator will explain that the participants are completely free to refuse to enter the study or to withdraw from it at any time, without any consequences for their further care and without need for justification. The investigator will complete and sign the informed consent section of the CRF for each participant enrolled.

11.3 Benefits and risks assessment, group relatedness

Benefits

NAFLD and NASH have an enormous and increasing prevalence: 20-30% of the gobal population has NAFLD to some extent.[8] NAFLD-related livercirrosis is a growing indication for liver transplantation and the incidence of NASH-related hepatocellular carcinoma is

increasing.[9–11] The disease progresses slowly and NASH-liver fibrosis is strongly associated with atherosclerotic cardiovascular disease and liver-related and overall mortality.[12,13] Nevertheless, there is no registered or proven treatment for progressed stages of NAFLD-NASH.

As previously stated, extensive prior research associates the gut microbiome with NAFLD-NASH. Regardless, these studies did not entail an treatment targeting the microbiome to reduce NAFLD-NASH, which would be a big step in the field. Based on available literature on treatment with synbiotics in NAFLD/NASH-patients and our pilot study on FMT in NAFLD/NASH-patients [27], this trial with adequate sample size endeavours to study if modification of the gut microbiome can attenuate NASH and liver fibrosis.

The benefits the study entails will be discussed in three sections. First, we expect a short term benefit for participants. Our underpowered study in which we administered FMT in NAFLD, showed a trend to reduction of necro-inflammation (i.e. inflammation and ballooning) in histopathological examination. Inflammation and ballooning are the most important predictors of progression of NAFLD-NASH, and subsequent secondary complications.[9] The current study will use LFMT capsules instead of fresh FMT per nasoduodenal tube. This will strongly reduce the burden on participants and will more closely resemble a realistic treatment for NASH. Moreover, recent studies show that LFMT capsules have the same efficacy and show a similar shift of the microbiota composition towards a donor profile, indicating similar engraftment. Half of the individuals will receive the LFMT capsules, and we expect that this already will attenuate NASH. All participants will, in addition, be treated with a complementary synbiotic combination. Multiple small clinical trials already showed a beneficial effect of synbiotics on NAFLD-NASH, without entailing serious side effects. Therefore, we expect a beneficial effect of the synbiotics in both study arms.

Second, participants will be able to benefit from the development of a treatment for NASH. As stated, NASH is a slowly progressing, chronic disease, without an effective treatment. If this study leads to a treatment of NASH, it is likely that participants in the future will receive the investigated treatment. Moreover, participants will assist in further understanding of the pathophysiology of NASH and its relation to the gut microbiome, possibly rendering further leads for the development of a therapy for NASH. Furthermore, the current study and its subsequent insights could not only lead to a treatment, but could also be the basis for preventive measures such as effective and relative easily applicable lifestyle and dietary interventions targeting the gut microbiome.

Last, the current study might benefit health on a population scale. As mentioned, the prevalence of NAFLD and NASH is high and increasing subsequent to the increase of its two main risk factors: obesity and T2DM. Fibrotic-NASH is strongly associated with atherosclerotic cardiovascular events and liver-specific and overall mortality. Considering the

enormous prevalence, this study and its possibilty to develop an effective treatment has the potential to benefit the global population on an tremendous scale. Furthermore, in comparison to medicinal alternatives currently investigated in the phase 2 and 3 trials for the treatment of NASH, FMT and synbiotics give a significant reduction of adverse effects.

Risks and burdens

The study protocol is quite intense for participants. In summary, the burden consists of the ingestion FMT-capsules (3x on study visits 21 capsules, daily 2 capsules) and daily ingestion of pre- en probiotica for 168 days, 2x a liver biopsy, 2x a MRI and Fibroscan, 4x 7 days of continuous glucose measurements and 5x blood withdrawal. The risk for participants will mainly be due to the liver biopsies, with a complication rate of approximately 1 in 1000 per procedure. Below, the risks and burdens are discussed more extensively.

FMT and synbiotics

In the literature and our center, FMT procedures have not been associated with (serious) adverse events. Literature concerning the treatment with FMT-capsules reports a lower number of adverse events in comparison to the infusion of FMT via duodenal tube. The most prevalent side effects are mild, self-resolving abdominal pain, nausea, diarrhea, and flatulence (see paragraph 6.4).

Concerning possible infectious transmission, feces donors are extensively screened to mitigate the risk of potential infections by the FMT. Moreover, feces donors are screened again after 2 months (during the storage of the capsules) prior to administration of the FMT-capsules to the participants. Participants with an immune deficiency are excluded from the study. In our center, FMT procedures have not been associated with adverse events, and donors are extensively screened to mitigate the risk of potential infections by the FMT procedure.

The provided synbiotics, moreover, are well-researched and deemed safe in both toxicological studies and trials in humans, with no serious side effects reported. The predominant burden of the FMT-capsules and synbiotics will consist of taking the capsules. On the days of the study visits (3x), participants will ingests 21 capsules of FMT and 3 probiotic capsules. Daily, participants will take 2 FMT-capsules, 3 probiotic capsules, and 5 grams of the fructo-oligosaccharide powder. The ingestion of the bulk of 21 FMT-capsules on the days of the study visits will be spread over the day to mitigate the theoretical risk of clotting of the capsules in the stomach (which might prevent normal passage to the duodenum). All capsules are designed to open in the duodenum; therefore, the content of the capsules will not reach the stomach or a higher part of the digestive tract.

Liver biopsies

To date, liver biopsy is still the golden standard in the assessment of progression or reduction of NASH, and there is no alternative. In therapy development for NASH, the American FDA, for example, demands liver biopsy in clinical trials. This study will, however, contribute to the further development and validation of multiparametric MRI as a substitute for biopsy by comparing the two.

An experienced interventional radiologist will perform ultrasound-guided liver biopsy. Ultrasound-guided percutaneous liver biopsy is a safe method with very low the risk of complications (< 1 per 1000 persons) comprising mostly bleeding from the biopsy site. Moreover, local hemostasis after the procedure can be observed, and patients will be screened for bleeding disorders. Liver biopsy will be performed under local anesthesia, which can cause short discomfort.

Continuous glucose monitoring

Participants will have a continuous glucose monitoring-device (Freestyle libre) installed for 4 times 7 days, in order to investigate the relation with GLP1. This is usually well tolerated (as we see in diabetic patients), however the prick of the installment can be unpleasant.

Blood withdrawal

Last, blood withdrawal, which happens 5 times, can be unpleasant, and can cause self-limiting (sub)cutaneous hemorrhage/bruising.

Diaries and questionnaires

Participants have to keep a diary of food-intake for 5 days in both the week before and after a study visit, and have to fill out two questionnaires on quality of life at baseline and week 24.

11.4 Compensation for injury

The sponsor/investigator has a liability insurance which is in accordance with article 7 of the WMO. The sponsor (also) has an insurance which is in accordance with the legal requirements in the Netherlands (Article 7 WMO). This insurance provides cover for damage to research participants through injury or death caused by the study. The insurance applies to the damage that becomes apparent during the study or within 4 years after the end of the study.

1. € 650.000,-- (i.e. six hundred and fifty thousand Euro) for death or injury for each subject who participate in the Research;

2. € 5.000.000,-- (i.e. five million Euro) for death or injury for all subjects who participate in the Research;
3. € 7.500.000,-- (i.e. seven million five hundred thousand Euro) for the total damage incurred by the organisation for all damage disclosed by scientific research for the Sponsor as ‘verrichter’ in the meaning of said Act in each year of insurance coverage.

11.5 Incentives

Donors of feces will receive €50,- per delivered stool sample (the stool for screening is included, blood withdrawal for screening will not be compensated).

All study participants will receive a financial compensation €350,- and a refund for travel costs. Financial compensation is based on the burden and the time spent in the study (30 hours per participant). The compensation thus equals approximately 12 euros per hour.

12. ADMINISTRATIVE ASPECTS, MONITORING AND PUBLICATION

12.1 Handling and storage of data and documents

Data will be collected on data collecting forms and will be entered after validation in a computer system for subsequent tabulation and statistical analysis. All research and medical data will be kept strictly confidential and registered under a unique study code. The key to this code will only be known to the principal- and coordinating investigators. Only the researchers that are involved in this study will be able to see the data and to identify a participant. All data will be kept at the AMC after the study has been completed, study material will be stored for a period of 15 years, expect for biological samples, which will be stored for a period of maximum 5 years. By signing the informed consent, participants allow future analysis of stored material. Healthy and Youth Care Inspectorate and representatives of the Amsterdam UMC as sponsor of this study have access to the data at any time. This information will be included in the written informed consent.

12.2 Monitoring and Quality Assurance

Monitoring will be done by an independent monitor provided by the CRU of the Amsterdam UMC, location AMC. The monitor will focus on the quality of data collection for the primary endpoint and patient safety. A monitoring plan is currently developed by the monitor.

12.3 Amendments

A ‘substantial amendment’ is defined as an amendment to the terms of the METC application, or to the protocol or any other supporting documentation, that is likely to affect to a significant degree:

- the safety or physical or mental integrity of the participants of the trial;
- the scientific value of the trial;
- the conduct or management of the trial; or
- the quality or safety of any intervention used in the trial.

All substantial amendments will be notified to the METC and to the competent authority.

Non-substantial amendments will not be notified to the accredited METC and the competent authority, but will be recorded and filed by the sponsor.

12.4 Annual progress report

The sponsor/investigator will submit a summary of the progress of the trial to the accredited METC once a year. Information will be provided on the date of inclusion of the first participant, numbers of participants included and numbers of participants that have completed the trial, serious adverse events/ serious adverse reactions, other problems, and amendments.

12.5 Temporary halt and (prematurely) end of study report

The sponsor will notify the accredited METC and the competent authority of the end of the study within a period of 90 days. The end of the study is defined as the last patient's last visit.

The sponsor will notify the METC immediately of a temporary halt of the study, including the reason of such an action.
In case the study is ended prematurely, the sponsor will notify the accredited METC and the competent authority within 15 days, including the reasons for the premature termination.

Within one year after the end of the study, the investigator/sponsor will submit a final study report with the results of the study, including any publications/abstracts of the study, to the accredited METC and the Competent Authority.

12.6 Public disclosure and publication policy

The trial will be registered in the Netherland Trial before study onset. The results of this study will be submitted for publication in an international, peer-reviewed journal.

13. STRUCTURED RISK ANALYSIS

Also see the structured risk analysis performed for the SYNCH-study (enclosed).

The present study is considered an intermediate risk study. In summary, the two liver biopsies are the main contributors of risk for participants.
Liver biopsies know a complication rate of approximately 1:1000, with self-limiting bleeding as the main adverse event. We will use experienced personnel to reduce the chance of complications. To date, liver biopsy is still the golden standard in the assessment of progression or reduction of NASH, and there is no alternative. In therapy development for NASH, the American FDA, for example, demands liver biopsy in clinical trials. This study will, however, contribute to the further development and validation of multiparametric MRI as a substitute for biopsy by comparing the two.
To date, FMT procedures have not been associated with (serious) adverse events. Literature concerning the treatment with LFMT capsules reports a lower number of adverse events in comparison to the infusion of FMT via duodenal tube. The most prevalent side effects are mild, self-resolving abdominal pain, nausea, diarrhea, and flatulence (also see paragraph 6.4). Donors will be extensively screened for infectious diseases, rendering the chance of translocation of virulent actors small.
Fructo-oligosaccharides, in addition, are a subgroup of dietary fibers, which are already part of a varied diet. They are safe, well-researched and already over-the-counter available. Moreover, *A. soehngenii, B. animalis, subsp. Lactis,* and *A. muciniphila* are commensals of the gut and all deemed safe to administer orally in toxicological studies and studies in humans, with no significant adverse event reported.
Last, we will withdraw blood (5x) and collect feces (7x), patients will complete several questionnaires and keep track of a food diary.
To evaluate and maximize the safety of participants, the study will be monitored by the clinical monitoring center and the DSMB.

NAFLD and NASH are very prevalent (20-30%) among the global population. Moreover, fibrotic-NASH entails a serious disease burden with increased risks of cardiovascular events and mortality. To date, there is no treatment for progressed stages of NAFLD-NASH. In follow up to a promising pilot-study, this trial endeavours to study if modification of the gut microbiome can attenuate NASH and liver fibrosis. The benefits this trial might hold is multiplex. First, we expect a short-term benefit for participants. Half of the individuals will receive the LFMT capsules, and we expect that this already will attenuate NASH. The current study will use LFMT capsules to severely reduce the burden on participants and will more closely resemble a realistic treatment for NASH in comparison to fresh FMT per

nasoduodenal tube. All participants will, in addition, be treated with a complementary synbiotic combination. Multiple small clinical trials already showed a beneficial effect of synbiotics on NAFLD-NASH, without entailing serious side effects.
Second, If this study leads to a treatment of NASH, it is likely that participants in the future will receive the investigated treatment. Furthermore, in comparison to medicinal alternatives currently investigated in the phase 2 and 3 trials for the treatment of NASH, FMT and synbiotics give a significant reduction of adverse effects. Participants will, in addition, assist in further understanding of the pathophysiology of NASH and its relation to the gut microbiome, possibly rendering further leads for the development of a therapy for NASH. Moreover, the current study and its subsequent insights could not only lead to a treatment, but could also be the basis for preventive measures such as effective and relative easily applicable lifestyle and dietary interventions targeting the gut microbiome.
Last, the current study might benefit health on a population scale. Considering the enormous prevalence and the serious disease burden, this study and its possibilty to develop an effective treatment has the potential to benefit the global population on an very large scale.

In summary, although there are risks due to the biopsies, these are necessary and the risks are limited. The present study, moreover, renders both short term and long term benefits for the participants, and may prevent more invasive interventions in the future. Furthermore, developing a treatment with neglible adverse effects for a disease with such a prevalence and health burden, would lead to an enormous health benefit on a population level. Therefore, we deem it legitimate to ask our participants to bear the burdens and risks to conduct the present study.

13.1 COVID-19

This study will adhere to the general guidelines regarding the safe practice of clinical studies during the COVID19 pandemic. Participants will be asked whether they have COVID19 related complaints prior to all study visits and adequate social distancing will be maintained throughout all study visits whenever possible, in concordance with current applicable guidelines. Processing of feces, in which COVID19 viral particles may be present, already happens in closed fume-hoods adhering to good laboratory practice. Donors will be explicitly asked to report any complaints related to COVID19 prior to donation. This study will not intervene with vaccine eligibility of the study participants or the donors.

14. Amendments

14.1 First amendment

The changes of the protocol in the first amendment are:

1. The delayed release ‘(DR) capsules will be replaced with ‘next generation enteric’ (NGE) capsules.
2. A questionaire to screen donors is added to the accessory documents.
3. The dates of the study visits (week 0, 8, 16 and 24) are changed into a range of 6 days each.
4. The screening of fecal donors is changed to a step by step procedure.
5. A procedure to deblind is added tot he protocol.
6. The erroneous use of ‘duodenal tube’ is corrected to ‘LFMT capsules’, conform the rest of the protocol.
7. The label of the LFMT capsules in the protocol is changed.
8. Not all non-serious AEs, but only non-serious AEs that possibly are related to the study are reported.
9. The erroneous 6 months interval of donor screening is corrected to 3 months in the protocol.

14.2 Second amendment

The changes of the protocol in the second amendment are:

1. The first MRI will be performed in the screening period.
2. Donors will not be primed with Wholefiber.
3. Participants will not be primed 3 days prior to the baseline visit.
4. An English version of the PIF and ICF is added.
5. The English versions of the CDLQ NAFLD-NASH and SF-36 are added.
6. The donor screening for COVID will be done through anamnesis, not serological.
7. The inclusion criterium that donor need to be white is removed.

15. REFERENCES

1. Smits LP, Kootte RS, Levin E, Prodan A, Fuentes S, Zoetendal EG, Wang Z, Levison BS, Cleophas MCP, Kemper EM, Dallinga-Thie GM, Groen AK, Joosten LAB, Netea MG, Stroes ESG, de Vos WM, Hazen SL, Nieuwdorp M. Effect of vegan fecal microbiota transplantation on carnitine- and choline-derived trimethylamine-N-oxide production and vascular inflammation in patients with metabolic syndrome. *J Am Heart Assoc*. Published online 2018. doi:10.1161/JAHA.117.008342
2. Kootte RS, Levin E, Salojärvi J, Smits LP, Hartstra A V., Udayappan SD, Hermes G, Bouter KE, Koopen AM, Holst JJ, Knop FK, Blaak EE, Zhao J, Smidt H, Harms AC, Hankemeijer T, Bergman JJGHM, Romijn HA, Schaap FG, Olde Damink SWM, Ackermans MT, Dallinga-Thie GM, Zoetendal E, de Vos WM, Serlie MJ, Stroes ESG, Groen AK, Nieuwdorp M. Improvement of Insulin Sensitivity after Lean Donor Feces in Metabolic Syndrome Is Driven by Baseline Intestinal Microbiota Composition. *Cell Metabolism*. Published online 2017. doi:10.1016/j.cmet.2017.09.008
3. Seegers JFML, Gül IS, Hofkens S, Brosel S, Schreib G, Brenke J, Donath C, de Vos WM. Toxicological safety evaluation of live Anaerobutyricum soehngenii strain CH106. *J Appl Toxicol*. Published online June 29, 2021. doi:10.1002/jat.4207
4. Kleiner DE, Brunt EM, Van Natta M, Behling C, Contos MJ, Cummings OW, Ferrell LD, Liu YC, Torbenson MS, Unalp-Arida A, Yeh M, McCullough AJ, Sanyal AJ. Design and validation of a histological scoring system for nonalcoholic fatty liver disease. *Hepatology*. Published online 2005. doi:10.1002/hep.20701
5. Younossi ZM, Golabi P, de Avila L, Paik JM, Srishord M, Fukui N, Qiu Y, Burns L, Afendy A, Nader F. The global epidemiology of NAFLD and NASH in patients with type 2 diabetes: A systematic review and meta-analysis. *Journal of Hepatology*. 2019;71(4):793-801. doi:10.1016/j.jhep.2019.06.021
6. Estes C, Razavi H, Loomba R, Younossi Z, Sanyal AJ. Modeling the epidemic of nonalcoholic fatty liver disease demonstrates an exponential increase in burden of disease. *Hepatology*. 2018;67(1). doi:10.1002/hep.29466
7. Van Den Berg EH, Amini M, Schreuder TCMA, Dullaart RPF, Faber KN, Alizadeh BZ, Blokzijl H. Prevalence and determinants of non-Alcoholic fatty liver disease in lifelines: A large Dutch population cohort. *PLoS ONE*. Published online 2017. doi:10.1371/journal.pone.0171502
8. Younossi Z, Anstee QM, Marietti M, Hardy T, Henry L, Eslam M, George J, Bugianesi E. Global burden of NAFLD and NASH: Trends, predictions, risk factors and prevention. *Nature Reviews Gastroenterology and Hepatology*. 2018;15(1). doi:10.1038/nrgastro.2017.109
9. Friedman SL, Neuschwander-Tetri BA, Rinella M, Sanyal AJ. Mechanisms of NAFLD development and therapeutic strategies. *Nature Medicine*. Published online 2018. doi:10.1038/s41591-018-0104-9
10. Arab JP, Arrese M, Trauner M. Recent Insights into the Pathogenesis of Nonalcoholic Fatty Liver Disease. *Annual Review of Pathology: Mechanisms of Disease*. 2018;13(1):321-350. doi:10.1146/annurev-pathol-020117-043617

11. Ruissen MM, Mak AL, Beuers U, Tushuizen ME, Holleboom AG. Non-alcoholic fatty liver disease: A multidisciplinary approach towards a cardiometabolic liver disease. *European Journal of Endocrinology*. Published online 2020. doi:10.1530/EJE-20-0065
12. Taylor RS, Taylor RJ, Bayliss S, Hagström H, Nasr P, Schattenberg JM, Ishigami M, Toyoda H, Wai-Sun Wong V, Peleg N, Shlomai A, Sebastiani G, Seko Y, Bhala N, Younossi ZM, Anstee QM, McPherson S, Newsome PN. Association Between Fibrosis Stage and Outcomes of Patients With Nonalcoholic Fatty Liver Disease: A Systematic Review and Meta-Analysis. *Gastroenterology*. Published online 2020. doi:10.1053/j.gastro.2020.01.043
13. Dulai PS, Singh S, Patel J, Soni M, Prokop LJ, Younossi Z, Sebastiani G, Ekstedt M, Hagstrom H, Nasr P, Stal P, Wong VWS, Kechagias S, Hultcrantz R, Loomba R. Increased risk of mortality by fibrosis stage in nonalcoholic fatty liver disease: Systematic review and meta-analysis. *Hepatology*. 2017;65(5). doi:10.1002/hep.29085
14. Targher G, Byrne CD, Lonardo A, Zoppini G, Barbui C. Non-alcoholic fatty liver disease and risk of incident cardiovascular disease: A meta-analysis. *Journal of Hepatology*. 2016;65(3). doi:10.1016/j.jhep.2016.05.013
15. Stols-Gonçalves D, Hovingh GK, Nieuwdorp M, Holleboom AG. NAFLD and Atherosclerosis: Two Sides of the Same Dysmetabolic Coin? *Trends in Endocrinology and Metabolism*. 2019;30(12). doi:10.1016/j.tem.2019.08.008
16. Donnelly KL, Smith CI, Schwarzenberg SJ, Jessurun J, Boldt MD, Parks EJ. Sources of fatty acids stored in liver and secreted via lipoproteins in patients with nonalcoholic fatty liver disease. *Journal of Clinical Investigation*. Published online 2005. doi:10.1172/JCI23621
17. Hardy T, Oakley F, Anstee QM, Day CP. Nonalcoholic Fatty Liver Disease: Pathogenesis and Disease Spectrum. *Annu Rev Pathol*. 2016;11(1):451-496. doi:10.1146/annurev-pathol-012615-044224
18. Isokuortti E, Zhou Y, Peltonen M, Bugianesi E, Clement K, Bonnefont-Rousselot D, Lacorte JM, Gastaldelli A, Schuppan D, Schattenberg JM, Hakkarainen A, Lundbom N, Jousilahti P, Männistö S, Keinänen-Kiukaanniemi S, Saltevo J, Anstee QM, Yki-Järvinen H. Use of HOMA-IR to diagnose non-alcoholic fatty liver disease: a population-based and inter-laboratory study. *Diabetologia*. Published online 2017. doi:10.1007/s00125-017-4340-1
19. DeFronzo RA. Insulin resistance, lipotoxicity, type 2 diabetes and atherosclerosis: The missing links. The Claude Bernard Lecture 2009. *Diabetologia*. Published online 2010. doi:10.1007/s00125-010-1684-1
20. Musso G, Cassader M, Paschetta E, Gambino R. Bioactive Lipid Species and Metabolic Pathways in Progression and Resolution of Nonalcoholic Steatohepatitis. *Gastroenterology*. Published online 2018. doi:10.1053/j.gastro.2018.06.031
21. Hafizi Abu Bakar M, Kian Kai C, Wan Hassan WN, Sarmidi MR, Yaakob H, Zaman Huri H. Mitochondrial dysfunction as a central event for mechanisms underlying insulin resistance: The roles of long chain fatty acids. *Diabetes/Metabolism Research and Reviews*. Published online 2015. doi:10.1002/dmrr.2601

22. Francque S, Vonghia L. Pharmacological Treatment for Non-alcoholic Fatty Liver Disease. *Advances in Therapy*. Published online 2019. doi:10.1007/s12325-019-00898-6
23. Koopman N, Molinaro A, Nieuwdorp M, Holleboom AG. Review article: can bugs be drugs? The potential of probiotics and prebiotics as treatment for non-alcoholic fatty liver disease. *Alimentary Pharmacology & Therapeutics*. Published online 2019. doi:10.1111/apt.15416
24. Aron-Wisnewsky J, Vigliotti C, Witjes J, Le P, Holleboom AG, Verheij J, Nieuwdorp M, Clément K. Gut microbiota and human NAFLD: disentangling microbial signatures from metabolic disorders. *Nature Reviews Gastroenterology and Hepatology*. Published online 2020. doi:10.1038/s41575-020-0269-9
25. Zhao S, Jang C, Liu J, Uehara K, Gilbert M, Izzo L, Zeng X, Trefely S, Fernandez S, Carrer A, Miller KD, Schug ZT, Snyder NW, Gade TP, Titchenell PM, Rabinowitz JD, Wellen KE. Dietary fructose feeds hepatic lipogenesis via microbiota-derived acetate. *Nature*. Published online 2020. doi:10.1038/s41586-020-2101-7
26. Yuan J, Chen C, Cui J, Lu J, Yan C, Wei X, Zhao X, Li NN, Li S, Xue G, Cheng W, Li B, Li H, Lin W, Tian C, Zhao J, Han J, An D, Zhang Q, Wei H, Zheng M, Ma X, Li W, Chen X, Zhang Z, Zeng H, Ying S, Wu JX, Yang R, Liu D. Fatty Liver Disease Caused by High-Alcohol-Producing Klebsiella pneumoniae. *Cell Metabolism*. Published online 2019. doi:10.1016/j.cmet.2019.08.018
27. Witjes JJ, Smits LP, Pekmez CT, Prodan A, Meijnikman AS, Troelstra MA, Bouter KEC, Herrema H, Levin E, Holleboom AG, Winkelmeijer M, Beuers UH, Lienden K, Aron-Wisnewky J, Mannisto V, Bergman JJ, Runge JH, Nederveen AJ, Dragsted LO, Konstanti P, Zoetendal EG, Vos W, Verheij J, Groen AK, Nieuwdorp M. Donor Fecal Microbiota Transplantation Alters Gut Microbiota and Metabolites in Obese Individuals With Steatohepatitis. *Hepatology Communications*. Published online 2020. doi:10.1002/hep4.1601
28. Udayappan S, Manneras-Holm L, Chaplin-Scott A, Belzer C, Herrema H, Dallinga-Thie GM, Duncan SH, Stroes ESG, Groen AK, Flint HJ, Backhed F, De Vos WM, Nieuwdorp M. Oral treatment with Eubacterium hallii improves insulin sensitivity in db/db mice. *npj Biofilms and Microbiomes*. Published online 2016. doi:10.1038/npjbiofilms.2016.9
29. Shetty SA, Zuffa S, Bui TPN, Aalvink S, Smidt H, De Vos WM. Reclassification of eubacterium hallii as Anaerobutyricum hallii gen. nov., comb. nov., and description of Anaerobutyricum soehngenii sp. nov., a butyrate and propionate-producing bacterium from infant faeces. *International Journal of Systematic and Evolutionary Microbiology*. Published online 2018. doi:10.1099/ijsem.0.003041
30. Consolandi C, Turroni S, Emmi G, Severgnini M, Fiori J, Peano C, Biagi E, Grassi A, Rampelli S, Silvestri E, Centanni M, Cianchi F, Gotti R, Emmi L, Brigidi P, Bizzaro N, De Bellis G, Prisco D, Candela M, D'Elios MM. Behçet's syndrome patients exhibit specific microbiome signature. *Autoimmunity Reviews*. Published online 2015. doi:10.1016/j.autrev.2014.11.009

31. Cholan PM, Han A, Woodie BR, Watchon M, Kurz ARM, Laird AS, Britton WJ, Ye L, Holmes ZC, McCann JR, David LA, Rawls JF, Oehlers SH. Conserved anti-inflammatory effects and sensing of butyrate in zebrafish. *Gut Microbes*. Published online 2020. doi:10.1080/19490976.2020.1824563
32. Ye Z, Zhang N, Wu C, Zhang X, Wang Q, Huang X, Du L, Cao Q, Tang J, Zhou C, Hou S, He Y, Xu Q, Xiong X, Kijlstra A, Qin N, Yang P. A metagenomic study of the gut microbiome in Behcet's disease. *Microbiome*. Published online 2018. doi:10.1186/s40168-018-0520-6
33. Bottacini F, Dal Bello F, Turroni F, Milani C, Duranti S, Foroni E, Viappiani A, Strati F, Mora D, van Sinderen D, Ventura M. Complete genome sequence of Bifidobacterium animalis subsp. lactis BLC1. *J Bacteriol*. 2011;193(22):6387-6388. doi:10.1128/JB.06079-11
34. Neyrinck AM, Rodriguez J, Taminiau B, Amadieu C, Herpin F, Allaert F-A, Cani PD, Daube G, Bindels LB, Delzenne NM. Improvement of gastrointestinal discomfort and inflammatory status by a synbiotic in middle-aged adults: a double-blind randomized placebo-controlled trial. *Sci Rep*. 2021;11(1):2627. doi:10.1038/s41598-020-80947-1
35. Plovier H, Everard A, Druart C, Depommier C, Van Hul M, Geurts L, Chilloux J, Ottman N, Duparc T, Lichtenstein L, Myridakis A, Delzenne NM, Klievink J, Bhattacharjee A, van der Ark KCH, Aalvink S, Martinez LO, Dumas M-E, Maiter D, Loumaye A, Hermans MP, Thissen J-P, Belzer C, de Vos WM, Cani PD. A purified membrane protein from Akkermansia muciniphila or the pasteurized bacterium improves metabolism in obese and diabetic mice. *Nat Med*. 2017;23(1):107-113. doi:10.1038/nm.4236
36. Belzer C, Chia LW, Aalvink S, Chamlagain B, Piironen V, Knol J, de Vos WM. Microbial Metabolic Networks at the Mucus Layer Lead to Diet-Independent Butyrate and Vitamin B12 Production by Intestinal Symbionts. *mBio*. 2017;8(5). doi:10.1128/mBio.00770-17
37. Depommier C, Everard A, Druart C, Plovier H, Van Hul M, Vieira-Silva S, Falony G, Raes J, Maiter D, Delzenne NM, de Barsy M, Loumaye A, Hermans MP, Thissen J-P, de Vos WM, Cani PD. Supplementation with Akkermansia muciniphila in overweight and obese human volunteers: a proof-of-concept exploratory study. *Nat Med*. 2019;25(7):1096-1103. doi:10.1038/s41591-019-0495-2
38. Kim S, Lee Y, Kim Y, Seo Y, Lee H, Ha J, Lee J, Choi Y, Oh H, Yoon Y. Akkermansia muciniphila Prevents Fatty Liver Disease, Decreases Serum Triglycerides, and Maintains Gut Homeostasis. *Appl Environ Microbiol*. 2020;86(7). doi:10.1128/AEM.03004-19
39. Druart C, Plovier H, Van Hul M, Brient A, Phipps KR, de Vos WM, Cani PD. Toxicological safety evaluation of pasteurized Akkermansia muciniphila. *J Appl Toxicol*. 2021;41(2):276-290. doi:10.1002/jat.4044
40. Koopen A, Witjes J, Wortelboer K, Majait S, Prodan A, Levin E, Herrema H, Winkelmeijer M, Aalvink S, Bergman JJGHM, Havik S, Hartmann B, Levels H, Bergh P-O, van Son J, Balvers M, Bastos DM, Stroes E, Groen AK, Henricsson M, Kemper EM, Holst J, Strauch CM, Hazen SL, Bäckhed F, De Vos WM, Nieuwdorp M,

Rampanelli E. Duodenal Anaerobutyricum soehngenii infusion stimulates GLP-1 production, ameliorates glycaemic control and beneficially shapes the duodenal transcriptome in metabolic syndrome subjects: a randomised double-blind placebo-controlled cross-over study. *Gut*. Published online October 25, 2021. doi:10.1136/gutjnl-2020-323297

41. Yoon HS, Cho CH, Yun MS, Jang SJ, You HJ, Kim J-H, Han D, Cha KH, Moon SH, Lee K, Kim Y-J, Lee S-J, Nam T-W, Ko G. Akkermansia muciniphila secretes a glucagon-like peptide-1-inducing protein that improves glucose homeostasis and ameliorates metabolic disease in mice. *Nat Microbiol*. 2021;6(5):563-573. doi:10.1038/s41564-021-00880-5
42. Armstrong MJ, Hull D, Guo K, Barton D, Hazlehurst JM, Gathercole LL, Nasiri M, Yu J, Gough SC, Newsome PN, Tomlinson JW. Glucagon-like peptide 1 decreases lipotoxicity in non-alcoholic steatohepatitis. *J Hepatol*. 2016;64(2). doi:10.1016/j.jhep.2015.08.038
43. Newsome PN, Buchholtz K, Cusi K, Linder M, Okanoue T, Ratziu V, Sanyal AJ, Sejling A-S, Harrison SA. A Placebo-Controlled Trial of Subcutaneous Semaglutide in Nonalcoholic Steatohepatitis. *New England Journal of Medicine*. Published online 2020. doi:10.1056/nejmoa2028395
44. Harrison SA, Wong VWS, Okanoue T, Bzowej N, Vuppalanchi R, Younes Z, Kohli A, Sarin S, Caldwell SH, Alkhouri N, Shiffman ML, Camargo M, Li G, Kersey K, Jia C, Zhu Y, Djedjos CS, Subramanian GM, Myers RP, Gunn N, Sheikh A, Anstee QM, Romero-Gomez M, Trauner M, Goodman Z, Lawitz EJ, Younossi Z. Selonsertib for patients with bridging fibrosis or compensated cirrhosis due to NASH: Results from randomized phase III STELLAR trials. *Journal of Hepatology*. Published online 2020. doi:10.1016/j.jhep.2020.02.027
45. Cortez-Pinto H, Borralho P, Machado J, Lopes MT, Gato I V., Santos AM, Guerreiro AS. Microbiota Modulation With Synbiotic Decreases Liver Fibrosis in a High Fat Choline Deficient Diet Mice Model of Non-Alcoholic Steatohepatitis (NASH). *GE Portuguese Journal of Gastroenterology*. Published online 2016. doi:10.1016/j.jpge.2016.01.004
46. Eslamparast T, Poustchi H, Zamani F, Sharafkhah M, Malekzadeh R, Hekmatdoost A. Synbiotic supplementation in nonalcoholic fatty liver disease: A randomized, double-blind, placebo-controlled pilot study. *American Journal of Clinical Nutrition*. Published online 2014. doi:10.3945/ajcn.113.068890
47. Manzhalii E, Virchenko O, Falalyeyeva T, Beregova T, Stremmel W. Treatment efficacy of a probiotic preparation for non-alcoholic steatohepatitis: A pilot trial. *Journal of Digestive Diseases*. Published online 2017. doi:10.1111/1751-2980.12561
48. Saxelin M, Tynkkynen S, Mattila-Sandholm T, de Vos WM. Probiotic and other functional microbes: from markets to mechanisms. *Curr Opin Biotechnol*. 2005;16(2):204-211. doi:10.1016/j.copbio.2005.02.003
49. de Vos WM. Systems solutions by lactic acid bacteria: from paradigms to practice. *Microb Cell Fact*. 2011;10 Suppl 1(Suppl 1):S2. doi:10.1186/1475-2859-10-S1-S2

50. Falony G, Vlachou A, Verbrugghe K, De Vuyst L. Cross-feeding between Bifidobacterium longum BB536 and acetate-converting, butyrate-producing colon bacteria during growth on oligofructose. *Applied and Environmental Microbiology*. Published online 2006. doi:10.1128/AEM.01296-06
51. Moens F, Verce M, De Vuyst L. Lactate- and acetate-based cross-feeding interactions between selected strains of lactobacilli, bifidobacteria and colon bacteria in the presence of inulin-type fructans. *International Journal of Food Microbiology*. Published online 2017. doi:10.1016/j.ijfoodmicro.2016.10.019
52. Lahti L, Salojärvi J, Salonen A, Scheffer M, de Vos WM. Tipping elements in the human intestinal ecosystem. *Nat Commun*. 2014;5:4344. doi:10.1038/ncomms5344
53. Carlson JL, Erickson JM, Hess JM, Gould TJ, Slavin JL. Prebiotic dietary fiber and gut health: Comparing the in vitro fermentations of beta-glucan, inulin and xylooligosaccharide. *Nutrients*. Published online 2017. doi:10.3390/nu9121361
54. De Groot P, Scheithauer T, Bakker GJ, Prodan A, Levin E, Khan MT, Herrema H, Ackermans M, Serlie MJM, De Brauw M, Levels JHM, Sales A, Gerdes VE, Ståhlman M, Schimmel AWM, Dallinga-Thie G, Bergman JJ, Holleman F, Hoekstra JBL, Groen A, Bäckhed F, Nieuwdorp M. Donor metabolic characteristics drive effects of faecal microbiota transplantation on recipient insulin sensitivity, energy expenditure and intestinal transit time. *Gut*. Published online 2019. doi:10.1136/gutjnl-2019-318320
55. Tian H, Ding C, Gong J, Wei Y, McFarland L v., Li N. Freeze-dried, Capsulized Fecal Microbiota Transplantation for Relapsing Clostridium difficile Infection. *Journal of Clinical Gastroenterology*. 2015;49(6):537-538. doi:10.1097/MCG.0000000000000330
56. Hecker MT, Obrenovich ME, Cadnum JL, Jencson AL, Jain AK, Ho E, Donskey CJ. Fecal Microbiota Transplantation by Freeze-Dried Oral Capsules for Recurrent Clostridium difficile Infection. *Open Forum Infectious Diseases*. 2016;3(2). doi:10.1093/ofid/ofw091
57. Haifer C, Paramsothy S, Borody TJ, Clancy A, Leong RW, Kaakoush NO. Long-Term Bacterial and Fungal Dynamics following Oral Lyophilized Fecal Microbiota Transplantation in Clostridioides difficile Infection. *mSystems*. 2021;6(1). doi:10.1128/mSystems.00905-20
58. Staley C, Hamilton MJ, Vaughn BP, Graiziger CT, Newman KM, Kabage AJ, Sadowsky MJ, Khoruts A. Successful Resolution of Recurrent Clostridium difficile Infection using Freeze-Dried, Encapsulated Fecal Microbiota; Pragmatic Cohort Study. *American Journal of Gastroenterology*. 2017;112(6):940-947. doi:10.1038/ajg.2017.6
59. Reigadas E, Olmedo M, Valerio M, Vázquez-Cuesta S, Alcalá L, Marín M, Muñoz P, Bouza E. Fecal microbiota transplantation for recurrent Clostridium difficile infection: Experience, protocol, and results. *Revista espanola de quimioterapia : publicacion oficial de la Sociedad Espanola de Quimioterapia*. 2018;31(5):411-418.
60. Jiang Z-D, Jenq RR, Ajami NJ, Petrosino JF, Alexander AA, Ke S, Iqbal T, DuPont AW, Muldrew K, Shi Y, Peterson C, Do K-A, DuPont HL. Safety and preliminary efficacy of orally administered lyophilized fecal microbiota product compared with frozen product given by enema for recurrent Clostridium difficile infection: A

randomized clinical trial. *PLOS ONE*. 2018;13(11):e0205064. doi:10.1371/journal.pone.0205064

61. Xu F, Li N, Wang C, Xing H, Chen D, Wei Y. Clinical efficacy of fecal microbiota transplantation for patients with small intestinal bacterial overgrowth: a randomized, placebo-controlled clinic study. *BMC Gastroenterology*. 2021;21(1):54. doi:10.1186/s12876-021-01630-x
62. Aroniadis OC, Brandt LJ, Oneto C, Feuerstadt P, Sherman A, Wolkoff AW, Kassam Z, Sadovsky RG, Elliott RJ, Budree S, Kim M, Keller MJ. Faecal microbiota transplantation for diarrhoea-predominant irritable bowel syndrome: a double-blind, randomised, placebo-controlled trial. *The Lancet Gastroenterology & Hepatology*. 2019;4(9):675-685. doi:10.1016/S2468-1253(19)30198-0
63. Bajaj JS, Salzman NH, Acharya C, Sterling RK, White MB, Gavis EA, Fagan A, Hayward M, Holtz ML, Matherly S, Lee H, Osman M, Siddiqui MS, Fuchs M, Puri P, Sikaroodi M, Gillevet PM. Fecal Microbial Transplant Capsules Are Safe in Hepatic Encephalopathy: A Phase 1, Randomized, Placebo-Controlled Trial. *Hepatology*. 2019;70(5):1690-1703. doi:10.1002/hep.30690
64. Allegretti JR, Kassam Z, Hurtado J, Marchesi JR, Mullish BH, Chiang A, Thompson CC, Cummings BP. Impact of fecal microbiota transplantation with capsules on the prevention of metabolic syndrome among patients with obesity. *Hormones*. 2021;20(1):209-211. doi:10.1007/s42000-020-00265-z
65. Yu EW, Gao L, Stastka P, Cheney MC, Mahabamunuge J, Torres Soto M, Ford CB, Bryant JA, Henn MR, Hohmann EL. Fecal microbiota transplantation for the improvement of metabolism in obesity: The FMT-TRIM double-blind placebo-controlled pilot trial. *PLoS Med*. 2020;17(3):e1003051. doi:10.1371/journal.pmed.1003051
66. Rinott E, Youngster I, Yaskolka Meir A, Tsaban G, Zelicha H, Kaplan A, Knights D, Tuohy K, Fava F, Scholz MU, Ziv O, Reuven E, Tirosh A, Rudich A, Blüher M, Stumvoll M, Ceglarek U, Clement K, Koren O, Wang DD, Hu FB, Stampfer MJ, Shai I. Effects of Diet-Modulated Autologous Fecal Microbiota Transplantation on Weight Regain. *Gastroenterology*. 2021;160(1):158-173.e10. doi:10.1053/j.gastro.2020.08.041
67. Dronkers TMG, Ouwehand AC, Rijkers GT. Global analysis of clinical trials with probiotics. *Heliyon*. 2020;6(7):e04467. doi:10.1016/j.heliyon.2020.e04467
68. Sakhare S, Shantanu C, Mopagar V, Hadpe H, Choughule K, Dahapute S, Shetty S, Joshi S. A comparative evaluation of probiotic formulations in prevention of dental caries: A clinical study. *Journal of Indian Society of Pedodontics and Preventive Dentistry*. 2021;39(4):416. doi:10.4103/jisppd.jisppd_236_21
69. Chen K, Zhang G, Xie H, You L, Li H, Zhang Y, Du C, Xu S, Melsaether C, Yuan S. Efficacy of *Bifidobacterium animalis* subsp. *lactis* , BB-12 ® on infant colic – a randomised, double-blinded, placebo-controlled study. *Beneficial Microbes*. 2021;12(6):531-540. doi:10.3920/BM2020.0233
70. Rezazadeh L, Alipour B, Jafarabadi MA, Behrooz M, Gargari BP. Daily consumption effects of probiotic yogurt containing Lactobacillus acidophilus La5 and

Bifidobacterium lactis Bb12 on oxidative stress in metabolic syndrome patients. *Clinical Nutrition ESPEN*. 2021;41:136-142. doi:10.1016/j.clnesp.2020.12.003

71. Scorletti E, Afolabi PR, Miles EA, Smith DE, Almehmadi A, Alshathry A, Moyses HE, Clough GF, Wright M, Patel J, Bindels L, Delzenne NM, Calder PC, Byrne CD. Design and rationale of the INSYTE study: A randomised, placebo controlled study to test the efficacy of a synbiotic on liver fat, disease biomarkers and intestinal microbiota in non-alcoholic fatty liver disease. *Contemporary Clinical Trials*. 2018;71:113-123. doi:10.1016/j.cct.2018.05.010
72. Nabavi S, Rafraf M, Somi MH, Homayouni-Rad A, Asghari-Jafarabadi M. Effects of probiotic yogurt consumption on metabolic factors in individuals with nonalcoholic fatty liver disease. *Journal of Dairy Science*. 2014;97(12):7386-7393. doi:10.3168/jds.2014-8500
73. Bottacini F, Dal Bello F, Turroni F, Milani C, Duranti S, Foroni E, Viappiani A, Strati F, Mora D, van Sinderen D, Ventura M. Complete Genome Sequence of Bifidobacterium animalis subsp. lactis BLC1. *Journal of Bacteriology*. 2011;193(22):6387-6388. doi:10.1128/JB.06079-11
74. Witjes JJ, Smits LP, Pekmez CT, Prodan A, Meijnikman AS, Troelstra MA, Bouter KEC, Herrema H, Levin E, Holleboom AG, Winkelmeijer M, Beuers UH, van Lienden K, Aron-Wisnewky J, Mannisto V, Bergman JJ, Runge JH, Nederveen AJ, Dragsted LO, Konstanti P, Zoetendal EG, de Vos W, Verheij J, Groen AK, Nieuwdorp M. Donor Fecal Microbiota Transplantation Alters Gut Microbiota and Metabolites in Obese Individuals With Steatohepatitis. *Hepatol Commun*. 2020;4(11):1578-1590. doi:10.1002/hep4.1601
75. Ooijevaar RE, Terveer EM, Verspaget HW, Kuijper EJ, Keller JJ. Clinical Application and Potential of Fecal Microbiota Transplantation. *Annu Rev Med*. 2019;70:335-351. doi:10.1146/annurev-med-111717-122956
76. Depommier C, Everard A, Druart C, Plovier H, van Hul M, Vieira-Silva S, Falony G, Raes J, Maiter D, Delzenne NM, de Barsy M, Loumaye A, Hermans MP, Thissen J-P, de Vos WM, Cani PD. Supplementation with Akkermansia muciniphila in overweight and obese human volunteers: a proof-of-concept exploratory study. *Nat Med*. 2019;25(7):1096-1103. doi:10.1038/s41591-019-0495-2
77. Larsen CN, Nielsen S, Kaestel P, Brockmann E, Bennedsen M, Christensen HR, Eskesen DC, Jacobsen BL, Michaelsen KF. Dose-response study of probiotic bacteria Bifidobacterium animalis subsp lactis BB-12 and Lactobacillus paracasei subsp paracasei CRL-341 in healthy young adults. *Eur J Clin Nutr*. 2006;60(11):1284-1293. doi:10.1038/sj.ejcn.1602450
78. Jungersen M, Wind A, Johansen E, Christensen JE, Stuer-Lauridsen B, Eskesen D. The Science behind the Probiotic Strain Bifidobacterium animalis subsp. lactis BB-12(®). *Microorganisms*. 2014;2(2):92-110. doi:10.3390/microorganisms2020092
79. Gibson GR, Hutkins R, Sanders ME, Prescott SL, Reimer RA, Salminen SJ, Scott K, Stanton C, Swanson KS, Cani PD, Verbeke K, Reid G. Expert consensus document: The International Scientific Association for Probiotics and Prebiotics (ISAPP)

consensus statement on the definition and scope of prebiotics. *Nat Rev Gastroenterol Hepatol*. 2017;14(8):491-502. doi:10.1038/nrgastro.2017.75

80. Swanson KS, de Vos WM, Martens EC, Gilbert JA, Menon RS, Soto-Vaca A, Hautvast J, Meyer PD, Borewicz K, Vaughan EE, Slavin JL. Effect of fructans, prebiotics and fibres on the human gut microbiome assessed by 16S rRNA-based approaches: a review. *Benef Microbes*. 2020;11(2):101-129. doi:10.3920/BM2019.0082
81. Bomhof MR, Parnell JA, Ramay HR, Crotty P, Rioux KP, Probert CS, Jayakumar S, Raman M, Reimer RA. Histological improvement of non-alcoholic steatohepatitis with a prebiotic: a pilot clinical trial. *Eur J Nutr*. 2019;58(4):1735-1745. doi:10.1007/s00394-018-1721-2
82. Behrouz V, Aryaeian N, Zahedi MJ, Jazayeri S. Effects of probiotic and prebiotic supplementation on metabolic parameters, liver aminotransferases, and systemic inflammation in nonalcoholic fatty liver disease: A randomized clinical trial. *J Food Sci*. 2020;85(10):3611-3617. doi:10.1111/1750-3841.15367
83. Scorletti E, Afolabi PR, Miles EA, Smith DE, Almehmadi A, Alshathry A, Childs CE, del Fabbro S, Bilson J, Moyses HE, Clough GF, Sethi JK, Patel J, Wright M, Breen DJ, Peebles C, Darekar A, Aspinall R, Fowell AJ, Dowman JK, Nobili V, Targher G, Delzenne NM, Bindels LB, Calder PC, Byrne CD. Synbiotics Alter Fecal Microbiomes, But Not Liver Fat or Fibrosis, in a Randomized Trial of Patients With Nonalcoholic Fatty Liver Disease. *Gastroenterology*. 2020;158(6):1597-1610.e7. doi:10.1053/j.gastro.2020.01.031
84. Mofidi F, Poustchi H, Yari Z, Nourinayyer B, Merat S, Sharafkhah M, Malekzadeh R, Hekmatdoost A. Synbiotic supplementation in lean patients with non-alcoholic fatty liver disease: a pilot, randomised, double-blind, placebo-controlled, clinical trial. *Br J Nutr*. 2017;117(5):662-668. doi:10.1017/S0007114517000204
85. Bouhnik Y, Vahedi K, Achour L, Attar A, Salfati J, Pochart P, Marteau P, Flourié B, Bornet F, Rambaud JC. Short-chain fructo-oligosaccharide administration dose-dependently increases fecal bifidobacteria in healthy humans. *J Nutr*. 1999;129(1):113-116. doi:10.1093/jn/129.1.113
86. Bouhnik Y, Neut C, Raskine L, Michel C, Riottot M, Andrieux C, Guillemot F, Dyard F, Flourié B. Prospective, randomized, parallel-group trial to evaluate the effects of lactulose and polyethylene glycol-4000 on colonic flora in chronic idiopathic constipation. *Aliment Pharmacol Ther*. 2004;19(8):889-899. doi:10.1111/j.1365-2036.2004.01918.x
87. Bouhnik Y, Raskine L, Simoneau G, Paineau D, Bornet F. The capacity of short-chain fructo-oligosaccharides to stimulate faecal bifidobacteria: a dose-response relationship study in healthy humans. *Nutr J*. 2006;5:8. doi:10.1186/1475-2891-5-8
88. Bonnema AL, Kolberg LW, Thomas W, Slavin JL. Gastrointestinal tolerance of chicory inulin products. *J Am Diet Assoc*. 2010;110(6):865-868. doi:10.1016/j.jada.2010.03.025
