# Supplementary material for: Randomised double-blind placebo-controlled trial protocol to evaluate the therapeutic efficacy of lyophilised faecal microbiota capsules amended with next-generation beneficial bacteria in individuals with metabolic dysfunction-associated steatohepatitis
Source: BMJ Open. 2025 Jan 9;15(1):e088290. doi: 10.1136/bmjopen-2024-088290 (PMC11784342; doi:10.1136/bmjopen-2024-088290)
Supplement: online supplemental file 5 [file bmjopen-15-1-s005.pdf]

Subject information for participation in medical scientific research

SYNCH - treating non-alcoholic fatty liver disease with inflammation and scarring with intestinal bacteria

Official title: Synbiotics and conditioned fecal microbiota transplantation to treat non-alcoholic steatohepatitis.

Introduction

Dear Reader,

With this information letter we would like to ask you if you would like to participate in medical scientific research. Participation is voluntary. You are receiving this letter because you have non-alcoholic fatty liver with inflammation (NASH) and possible scarring (fibrosis). This letter will tell you what type of research this is, what it means for you, and what the advantages and disadvantages are. It is a lot of information. We would like you to read through the information and decide if you want to participate. If you want to participate, please fill out the form found in Appendix G.

Ask your questions

You can make your decision with the information found in this information letter. In addition we encourage you to:

- Ask questions of the researcher giving you this information.
- Talk to your partner, family or friends about this study.
- Ask questions of the independent expert, Dr. N.M.J. Hanssen.
- Read the information at www.rijksoverheid.nl/mensenonderzoek.

1. General information

This research is being conducted by Drs. Quinten Augustijn, physician-investigator, and Dr. Onno Holleboom, internist-vascular medicine and endocrinologist at the Amsterdam UMC, AMC location. The medical ethics review committee of the AMC has approved this study.

2. What is the purpose of this research?

In this study, we are looking at how well a poop transplant (in medical language: 'fecal microbiota transplantation'; hereafter, we will name this FMT), supplemented with three gut bacteria and fiber, can treat NASH.
We compare the effect of the FMT with the effect of a placebo. A placebo is a drug with no active ingredient, a "fake drug”.

3. What is the background for this research?

Non-alcoholic fatty liver disease (NAFLD) is a highly prevalent condition that can lead to inflammation and scarring (also known as fibrosis) of the liver, resulting in non-alcoholic steatohepatitis (NASH). Research has shown that when fibrosis occurs, the risk of serious health problems increases, including an elevated risk of cardiovascular disease, and, in lesser extent, liver cancer, and premature death. Unfortunately, apart from adopting a healthier lifestyle, such as losing weight and increasing exercise, there are no proven effective treatments for NASH.

Although the exact mechanisms by which NASH and fibrosis develop are not fully understood, research indicates that insulin resistance, a condition in which the hormone that regulates sugar in the blood is less effective, plays a significant role in their development. This helps explain why NAFLD and NASH are often associated with other metabolic conditions such as type 2 diabetes and obesity. Additionally, studies have shown that the composition of gut bacteria also plays a major role in NASH development.

The human intestines contain a large number of bacteria that convert the nutrients we eat and drink into other substances. The type of bacteria in the gut determines the resulting substances that are produced. Some bacteria convert substances differently than others, and some can affect how well the intestinal wall keeps out unhealthy substances. In patients with NAFLD and NASH, certain types of "bad" bacteria are prevalent, while "good" bacteria are almost nonexistent. Studies have also shown that giving "good" bacteria to animals or humans can improve NASH.

In this study, we aim to investigate the efficacy of using fecal microbiota transplantation (FMT) from healthy donors and probiotics, along with prebiotic nutrition for good bacteria, to improve NASH. While this may sound strange, it actually makes a lot of sense. After all, stool contains an enormous amount of bacteria. So when you give stool from a healthy and pretreated donor, you can give a lot of good bacteria. It is also good that these bacteria already lived together, and therefore already work well together. This research group has previously conducted a study with FMT in people with NAFLD. That study gave a strong

indication that inflammation and scarring decreased in people with NAFLD and NASH, but included too few subjects to really prove it. The second method is to give certain types of good bacteria in a capsule - probiotics. These probiotics can, for example, convert nutrients to good substances or cause the gut to absorb fewer bad substances. We hope that by supplementing the FMT with good bacteria, we can esthablish an even bigger improvement of NASH. Several studies already showed a beneficial effect, but not yet large enough. We will give bacteria that cooperate with each other to form good substances and show a beneficial effect on the intestinal wall in studies.
Finally, in addition to the FMT and probiotics, we will also give nutrition for the good bacteria ("prebiotics"). These foods for the bacteria are fibers (like those found in fruits and vegetables) that give the bacteria energy and are the source for making the healthy substances.
In summary, given the increasing prevalence and serious health implications of NASH and the lack of drug treatments, we are investigating a novel approach to treating NASH by targeting the gut bacteria. By improving the composition of gut bacteria, we want to improve the hepatic fatty accumulation, inflammation and scarring. We will change that composition of bacteria by giving FMT capsules, bacteria in capsules and nutrition for those bacteria.

4. How will this research proceed?

How long will the study take?
Are you participating in the study? If so, the study will take a total of 24 weeks.

Step 1: Are you eligible to participate in the study?
We first want to know if you are suitable to participate. Therefore, the researcher does a number of tests:

An interview.
You will have an interview with the researcher, during which they will ask you questions to determine if you are a good fit for the study.

Physical examination.
The examiner will for example listen to your heart and lungs, measure your blood pressure, and check your heart rate.

Blood test.
The researcher will take a blood sample to check for blood clotting and to make sure you don't have any infectious diseases like hepatitis B or C.

Examination of your medical history.
The researcher will check your medical history to ensure you don't have any conditions or medications that could seriously affect the study, or have conditions that make it unsafe for you to participate in the study.

<u>MRI</u>
We will perform an MRI before the liver biopsy and at the end of the study (at the visit of 24 weeks). This allows us to get a good look at the liver. The MRI scan takes about 45 minutes. If you have claustrophobia, you will not get an MRI.

<u>Liver biopsy.</u>
If you are eligible for the study, we will take a liver biopsy. This is a little piece of the liver. An experienced doctor ('interventional radiologist') will, after numbing the skin, puncture the liver while guided by ultrasound, so that he/she can clearly see where the puncture is happening. Then two other doctors ("pathologists") will assess how bad the liver fattening, inflammation and scarring is. If this is not bad enough, or in rare cases too bad, you cannot participate in the study. If you had a liver biopsy in the 32 weeks before the study began, it does not need to be repeated and that liver biopsy will be re-evaluated for participation.

Step 2: treatment.
If you are eligible for the study, we will treat you in several ways. All participants will receive:

- Probiotics: These are bacteria found in the gut. We will give you three types of bacteria called Bifidobacterium animals subspp. lactis, Anaerobutyricum soehngenii, and pasteurized Akkermansia muciniphila. You will take three capsules of these bacteria each day for 24 weeks.
- Prebiotics: This is a sweet powder called fructo-oligosaccharide that you can dissolve in water, coffee, or tea. You will take this powder every day for 24 weeks.

We will also divide participants into two groups:

- Group 1: These participants will receive FMT capsules.
- Group 2: These participants will receive placebo capsules.

Half of the participants will receive FMT capsules and the other half will receive placebo capsules. You and the researcher will not know which group you are in.

On the days of the first three study visits, participants will receive 21 capsules on one day, spread throughout the day. On every other day of the study, participants will take 2 FMT/placebo capsules. The other half of the participants will receive placebo capsules (which contain water with salt and a dye) instead of FMT capsules, so we can compare these groups. You can read exactly what this means in the next section. Lottery determines which treatment you get. Neither you nor the researcher know which group you are in. However, if it is important for your health, this can be looked up.

Step 3: examinations and measurements
after the screening, you will need to visit Amsterdam UMC, location AMC four times over 24 weeks. The duration of a visit varies. There are 2 visits that take about 7 hours (a whole

working day), and 2 visits that take about 3 hours. During these days you must fast (not eat or drink, except for a little water) for an extended period of time. In addition, you will be asked to send in your stool samples 3 times. The tests we do vary from time to time. The first and last day are similar, and the visit after 8 and 16 weeks are similar. Below we first discuss treatment with the FMT and then the tests.

Appendix D contains a schedule of what examinations and tests we do at which visit.

Fecal microbiota transplantation (FMT)

We will schedule three study visits at which you will have examinations and take a large amount (namely: 21) of FMT capsules or placebo capsules. We can imagine that you may find the thought of taking FMT capsules crazy or unpleasant. Therefore, we feel it is important to mention that the capsules themselves are odorless and tasteless, and open only after they have already passed through the stomach. In this way, we ensure that the contents of the capsules cannot enter the stomach, esophagus or mouth. During the visits, you will be accompanied by an experienced team, who will discreetly and skillfully guide you throughout taking the capsules and undergoing the examinations.
We will ask you to take a laxative the day before your first visit to help the new bacteria stay in your bowel. On all four days of the examinations, we ask that you bring your stool from the morning. It is important that this is as fresh as possible.

Examinations
We do the following examinations:

Physical examination (4x).

The examiner will listen to your heart and lungs and take various measurements such as your blood pressure, height, and weight. This will be done four times during the study.

Blood examination (4x).

The examiner will take several tubes of blood from you at each of the four visits. Together, we will take between 40 and 60 mL of blood from you per visit. With the blood test, we test several factors such as liver function, fats, sugar, inflammation, and substances important in NASH and its development. We also check your blood for general issues to ensure your safety. Blood clotting will also be checked before you undergo a liver biopsy to make the procedure as safe as possible.

Fecal examination (7x).

You will be asked to submit stool samples at all four visits. It is preferable that you submit stool samples that you have produced the morning before the visit, preferably refrigerated. In addition, we ask that you submit three more stool samples, and we will provide you with materials and instructions for storage and sending.

Ultrasound (Fibroscan) of the liver (2x).
We will do a type of ultrasound examination of liver on the first and last examination. This is a painless external ultrasound measurement of your liver that takes a few minutes.

Liver biopsy (2x, at screening and at 24 weeks).
A liver biopsy will be performed twice, at the screening and 24-week visits, to assess the treatment's effectiveness in improving NASH.

Blood sugar measurement (4x).
A sticker with a tube inserted into the skin on your upper arm for 7 days that can measure your blood sugar (Freestyle libre) will be given to you four times during the study.

Questionnaires (2x).
You will complete two questionnaires on both the first and the last visit. The questions are about your symptoms and how you are doing with your condition.

Dietary diary
You will keep a diet diary at home, 5 days a week before and 5 days a week after the study visits, to see what effect diet has on gut bacteria. You can do this online at: https://mijn.voedingscentrum.nl/nl/eetmeter/.

What is different from regular care?
Participating in this study means you will receive more tests than you would in regular care. For example, blood will be drawn more frequently, and you will undergo scans and biopsies, which you would not otherwise undergo. However, there will be no change to your regular appointments with your doctor.

5. Which agreements do we make with you?

To ensure the study goes according to plan, we make the following agreements with you:

- You will take the capsules containing the FMT, bacteria, and powder as explained by the researcher.
- You will store the study drug as instructed and return any unused and empty containers at the next visit.
- You are not participating in any other medical science study during this study.
- You will attend every appointment.
- You must come to the hospital sober on the 4 days you visit and receive tests. This means that you may not eat or drink anything for 6 hours before the examinations. However, you may drink water.
- You will carry the study participant card with you, stating that you are participating in this study and who to notify in case of an emergency.
- For women: You must not be or become pregnant during the study and must not breastfeed.

- You will contact the investigator in these situations:
  - You want to start taking other medications, for example an antacid or antibiotics. Also, if these are homeopathic remedies, natural remedies, vitamins or drugs from the drugstore, please contact us.
  - You are admitted or treated in a hospital.
  - You suddenly experience problems with your health.
  - You no longer wish to participate in the study.
  - Your phone number, address or e-mail address changes.

Can you or your partner become pregnant during the study?
Women who are pregnant or breastfeeding cannot take part in the study. You also should not become pregnant during the study because we don't know if the treatment is safe for pregnant or breastfeeding women.

If you do become pregnant during the study, please let the researcher know right away and stop the study.

6. What side effects, adverse effects or discomforts may you experience?

Taking 21 capsules of FMT in one day may cause side effects, namely:
- Short-term abdominal pain,
- Nausea (the capsules do not open until after the stomach, so there is no risk of vomiting the contents of the capsules).

The capsules containing bacteria and the powder (the food for the bacteria) often give (in more than 1 in 10 of persons)
- Slightly increased flatulence ("farting")
- Slightly increased abdominal rumbling
- A bloated abdomen
- Uncomfortable feeling in the abdomen.

7. What are the advantages and disadvantages of participating in the study?

Participating in the study can have advantages and disadvantages. Below we list them. Think about these carefully, and talk about them with others.

Advantages
The therapy may improve NASH but it is not certain. In addition, with your participation, you will help researchers understand more about the treatment of NASH and the mechanism by which gut bacteria exert an effect on the liver.

Participating in the study may have these disadvantages:

- You may suffer from the side effects or adverse effects of pre- and probiotics and capsule poop transplantation, as described in Section 6.
- You may be bothered by the measurements during the study.
- Because we transfer microorganisms with the FMT capsules, there is also a theoretical risk of infection from the donor, but we screen very extensively (including HIV, hepatitis B and antibiotic-resistant organisms, etc) to make this chance almost zero.
- Bowel laxation on the day prior to the first study visit results in frequent toilet visits on that day.
- Liver biopsy has a risk of about 1 in 1000 that you may have minor bleeding. We will then need to observe you in the hospital for several hours.
- The blood draw may cause some pain and you may experience bruising as a result.
- The questionnaires can sometimes be perceived as confronting.

Participation in the study also means:

- That it will cost you extra time
- Additional examinations and tests
- That you must keep the agreements made.

It is possible that during the study something is accidentally discovered that is not directly relevant to the study but is important for your health or that of your family members. In this case, your own primary care physician or specialist will discuss with you what to do next. The costs are covered by your own health insurance.

Don't want to participate?

It is up to you whether or not to participate in the study. Declining to participate will never affect the relationship with your physician or your quality of care.

8. When does the research end?

The researcher will let you know if there is any new information about the study that is important to you. The researcher then asks you if you will continue to participate.

In these situations, the study stops for you:

- All studies according to the schedule are over.
- The end of the whole study is reached after the visit after 24 weeks.
- You have become pregnant.
- You want to stop the study yourself. You may do so at any time. Please report this to the investigator immediately. You do not have to say why you are stopping. You will return to your regular care for your NASH. The investigator may schedule one or more more checkups for your safety.
- The researcher feels it is better for you to stop. However, the investigator will still invite you for follow-up checks.
- One of the following decides that the study should stop:

o the Amsterdam UMC,
o the government, or
o the medical ethics committee reviewing the research.

What happens if you stop the research?
The researchers will use the data and biopsies, scans, stool samples and blood samples collected up to the time of stopping. If you want, collected body material can be destroyed. You can inform the researcher of this.

The entire study ends when all participants are finished.

9. What happens after the research is finished?

Can you continue to use the capsules?
You cannot continue the capsules after the study.

Will you receive the results of the study?
About 6-12 months after the study is completed, the researcher will let you know the main results of the study. The researcher may also tell you what treatment you received. Do you not want to know this? If so, please inform the researcher. He will then not tell you.

10. What we do with your data and bodily materials?

Are you participating in the study? Then you also give us permission to collect, use and store your data and body material.

What data do we store?
We save the following data
- your name
- your gender
- your address
- your date of birth
- your ethnicity
- data about your health
- (medical) data we collect during the study
- scans

What body material do we store?
We store
- Blood samples
- Liver biopsies
- Stool samples

Why do we collect, use and store your data and body material?

We collect, use and store your data and body material to answer the questions of this study. And to publish the results.

How do we protect your privacy?
To protect your privacy, we code your data and body material. We put only this code on all your data and body material. We keep the key to the code in a secure place in the hospital. When we process your data and body material, we always use only this code. Even in reports and publications about the research, no one can recall that it was about you.

Who can see your data?
Some people can see your name and other personal data without a code. These are people who check whether the researchers are conducting the research properly and reliably. These individuals can access your data:
- Members of the committee monitoring the safety of the research.
- Controllers of the Amsterdam UMC
- Inspectors from the Health Care and Youth Inspectorate.

These persons will keep y our data confidential. We ask you to give permission for this access.

How long do we keep your data and bodily material?
We keep your data in the hospital for 15 years.
We keep your body material in the hospital. It is kept for 5 years so that new measurements can be made on it during the course of this study that are related to this study. As soon as this is no longer necessary, we will destroy your body material.

May we use your data and body material for other research?
Your data and your (remaining) body material may also be important for other scientific research in the field of NASH after this study is completed. For this purpose, your data and body material will be kept in the hospital for 5 years. In the consent form, please indicate whether you approve of this. Do you not give your consent? Then you can still participate in this study. You will receive the same care.

What happens in case of unexpected discoveries?
During the study, we may accidentally find something important to your health or to the health of your family members. The researcher will then contact your primary care physician, or if necessary, your specialist. You will then discuss with your primary care physician or specialist what to do. You consent to informing your family doctor or specialist with the form.

Can you withdraw your consent to the use of your data?
You can withdraw your consent to the use of your data at any time. But please note: if you withdraw your consent when researchers have already collected data for the study, then they may still use this data. For your bodily material, the researchers will destroy it after you

withdraw your consent. However, if measurements are already been done on your body material, the researcher may continue to use these results.

Want to know more about your privacy?

- Would you like to know more about your rights when processing personal data? Please visit www.autoriteitpersoonsgegevens.nl.
- Do you have any questions about your rights? Or do you have a complaint about the processing of your personal data? If so, please contact the person responsible for processing your personal data. For your research this is:
- Amsterdam UMC, location AMC. See Appendix F for contact information, and website.
- If you have complaints about the processing of your personal data, we recommend that you first discuss them with the research team. You can also go to the Data Protection Officer at Amsterdam UMC, location AMC. Or you can file a complaint with the Authority for Personal Data.

Where can you find more information about the study?

You can find more information about the study on the following website(s): www.clinicaltrials.gov. After the survey, the website may show a summary of the results of this survey. You can find the study by searching NL81001.018.22.

11. Will you receive compensation if you participate in the study?

The research resources, additional tests and treatment for the study will cost you nothing. For participating in this study, you will receive an expense allowance of €350. In addition, you will be reimbursed for travel expenses. Do you stop before the study ends? Then you will receive part of the compensation (per research visit €75,-). The compensation for participating in this study will be reported to the Tax Office as income.

12. Are you insured during the research?

Insurance is provided for everyone participating in this study. The insurance pays for damages caused by the study. But not for all damages. Appendix E provides more information about the insurance and the exceptions. It also tells you who you can report damages to.

13. We inform your general practitioner

The researcher will send your doctor an electronic letter to let you know that you are participating in the study. This is for your own safety. In an emergency situation, we may contact your primary care physician, such as about your medical history or the medications you are taking.

14. Do you have questions?

You can ask questions about the study to the researcher. Would you like advice from someone who has no interest? Then go to Dr. N.M.J. Hanssen. He knows a lot about the study, but does not participate in this study.
Do you have a complaint? Please discuss this with the researcher or the doctor treating you. Would you rather not? Then go to the complaints officer of the Amsterdam UMC, location AMC. Appendix F shows where you can find them.

15. How do you consent to the study?

You can think about this research in first. Then you will tell the researcher if you understand the information and whether or not you want to participate. Do you want to participate? Then fill out the consent form found in Appendix G to this information letter. You and the researcher will both get a signed version of this consent form.

Thank you for your time.

16. Attachments to this information

A. Instructions to prepare for blood sampling study visits.
B. Instructions for the dietary diary
C. Instructions for morning stool collection
D. Schedule of study visits
E. Insurance information
F. Contact details Amsterdam UMC, location AMC.
G. Consent form test subject.

Appendix A: Instructions for preparing study visits.

If you are asked to come fastened, please adhere to the following instructions:

- Keep your eating habits the same prior to the visits.
- During fasting, water (1 glass of water in the evening and 1 glass on the morning of the intake) is allowed, but coffee, tea, milk or juice are not allowed. It is very important that you adhere to this.
- You may take your prescribed home medication as usual the morning of the study (with water!), except if you are taking insulin.
- If you use long-acting insulin in the evening, use half (50%) of your normal dose the night before the study appointment. If you normally use short-acting insulin with meals, you do not need to inject short-acting insulin on the study day when you are fastened. If you are in doubt, have questions or are still not completely clear on what exactly you should do, you can always consult the physician-investigator of this study. Please don’t hesitate to do so.
- Avoid any major physical strain 24 hours prior to the blood draw and/or liver biopsy.
- Blood and/or plasma donation during the study is not allowed and there must be a donation-free period of at least 1 month before the first visit.
- If you are ill (e.g., flu/severe cold), please contact the investigator. Infections can have major effects on study results. The visit might have to be postponed.

Appendix B: Instructions for dietary diary

For the SYNCH study, you will collect data about your diet before the study visits. You will do this in the week before and after each study day. Try to keep track of 4-5 days during those weeks. We use the Nutrition Center's "Eetmeter" for this purpose. Below are instructions for the online diet diary.

Directions

- Keep the diet diary at least **4-5 consecutive days** in the week before and after the study visit.
  - Make sure at least 1 day falls in the weekend
  - Collect more than 4-5 days in the diet diary anytime.
- Record **everything you eat and drink**, preferably immediately after consumption so that you do not forget anything.
- **Describe** the foods you eat **as accurately as possible** (e.g., all individual ingredients in salads and sauces).
- Record the **quantity of each food** item as accurately as possible (in grams, deciliters, teaspoons/dishes, slices, etc.).
- Also note the **quality** as accurately as possible (e.g., semi-skimmed milk, cheese with 24% fat, whole grain bread, etc.).
- Consider the **method of preparation** (oven, baked, cooked) and the use of fats and oils when baking.

Create Account

1. Go to https://mijn.voedingscentrum.nl/
2. Click on register and enter your information here.
3. Activate your account.

Fill out diet diary

1. Log in with your login credentials: https://mijn.voedingscentrum.nl/nl/login/. 'Eetmeter' can also be downloaded as an app on your smartphone.
2. Below you will see an overview of the interface.

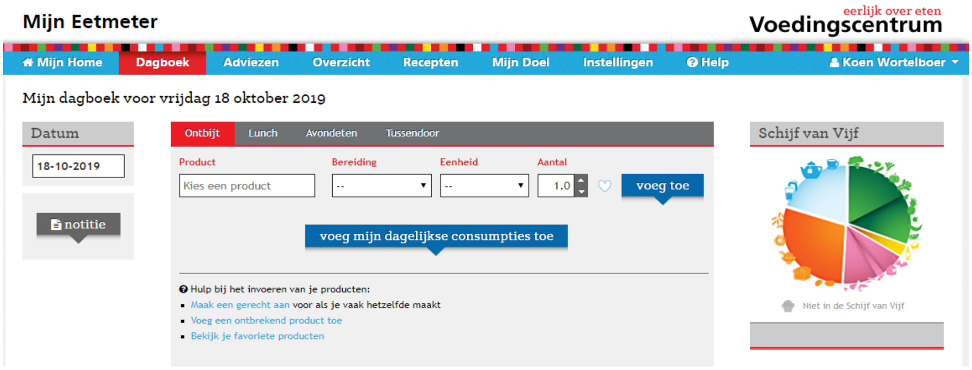

3. Here you record everything you eat and drink at breakfast, lunch, dinner and in between meals.
4. Add product or ingredient:
    a. Look up the product or ingredient. Note its quality (whole grain bread, skim milk, etc.).
    b. If applicable, select a preparation method (boiled, baked, etc.).
    c. Select a unit (grams, ladle, etc.)
    d. Select a number (how many grams/spoonfuls)
5. You can remove products by clicking on the red cross to the right of the products.
6. If desired, you can add a note to the day.
7. Below is an example of a completed diary.

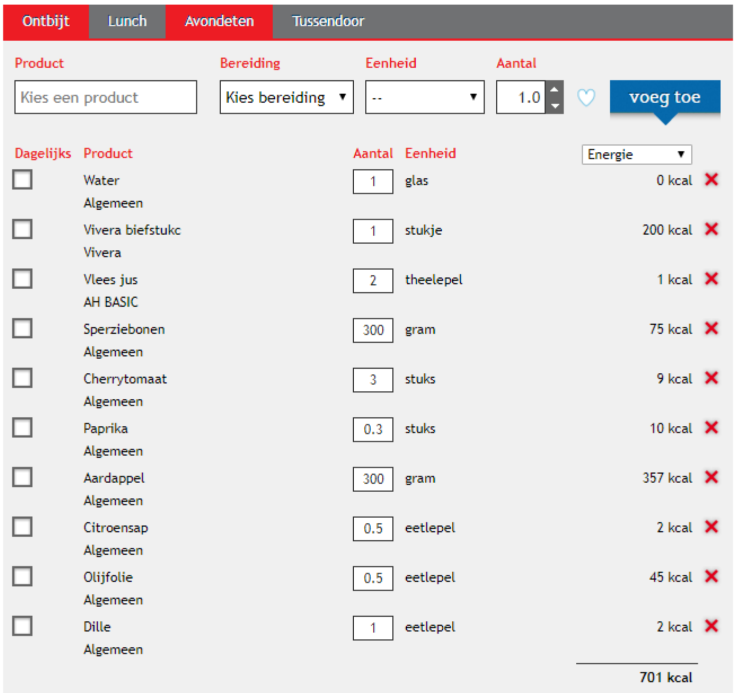

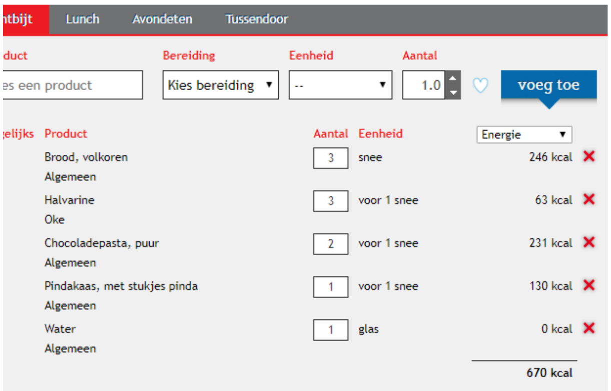

Exporting and sending data

After data collection, we will ask you to send us your diet diary. To do this, you must download your diet diary and send it to the researcher by e-mail. We can also do it together during the visit. You will do this as follows:

1. Go to "Overview" and under "date" select the appropriate week number(s) (see below).
2. Press the "Excel" button to download the summary in Excel format.
3. Send the data by mail to the researcher: q.j.augustijn@amsterdamumc.nl

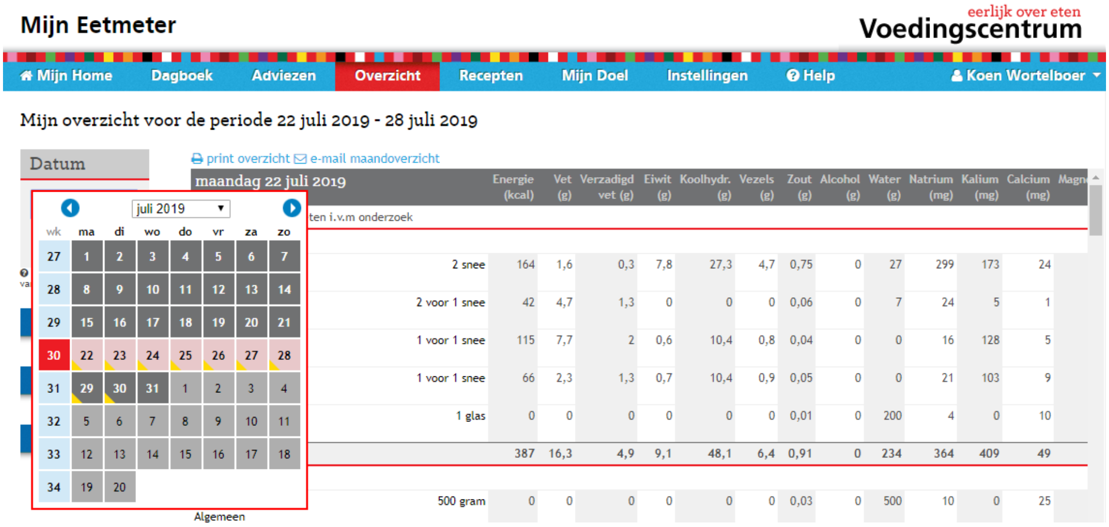
Mijn Eetmeter
eerlijk over eten
Voedingscentrum
Mijn Home
Dagboek
Adviezen
Overzicht
Recepten
Mijn Doel
Instellingen
Help
Koen Wortelboer
Mijn overzicht voor de periode 22 juli 2019 - 28 juli 2019
Datum
print overzicht
e-mail maandoverzicht
maandag 22 juli 2019
juli 2019
Energie (kcal)
Vet (g)
Verzadigd vet (g)
Eiwit (g)
Koolhydr. (g)
Vezels (g)
Zout (g)
Alcohol (g)
Water (g)
Natrium (mg)
Kalium (mg)
Calcium (mg)
ten i.v.m onderzoek
2 snee
2 voor 1 snee
1 voor 1 snee
1 glas
500 gram
Algemeen

Appendix C: Instructions for morning stool collection.

For the SYNCH study, we ask you to collect morning stool before your visit, and separately send in your stool 3 times. Stools will thus be collected a total of 7 times.

Below are instructions for stool collection.

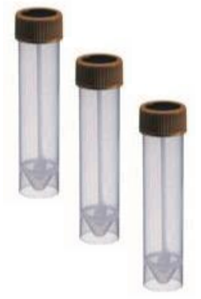

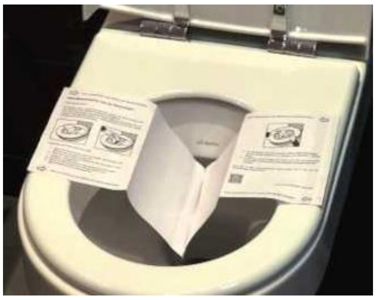

The package contains the following

- Spoon tubes with brown cap 3x
- Feces collector 1x
- Instructions for collection and return.

Collection morning stool

1. Collect your stool in the toilet using the feces catcher. Follow the instructions on the feces collector.
2. Fill three spoon tubes with stool using the spoon attached to the cap. Fill the tube (maximum) halfway with feces.
3. Screw the cap firmly onto the spoon tubes.
4. The used feces trap can be flushed down the toilet.
5. Please note that all tubes already contain a label with your information. Please return the tubes as soon as possible (see below).

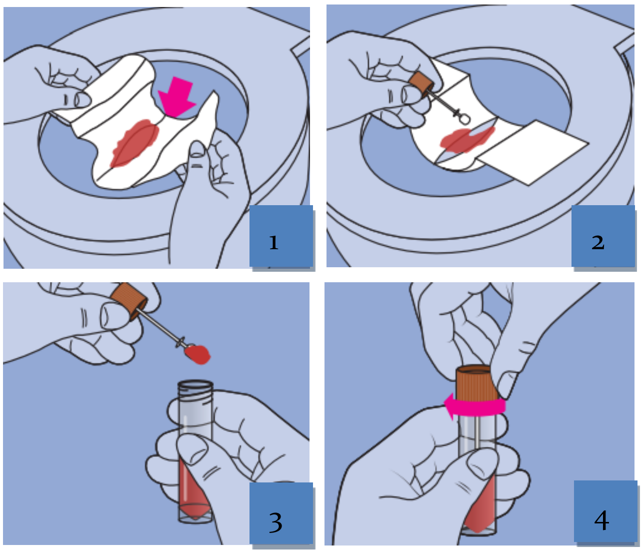

Collection and delivery of feces

- Collect your stool as indicated above on the morning before your first, midterm and final study visit.
- After filling the spoon tubes, these should be brought with you to the study visit. The tubes can be stored temporarily at room temperature.
- If you cannot collect stool in the morning, you can collect stool a day in advance. Then store the spoon tubes in the refrigerator and remember to bring them to the study visit.

Appendix D: Schematic overview of study visits

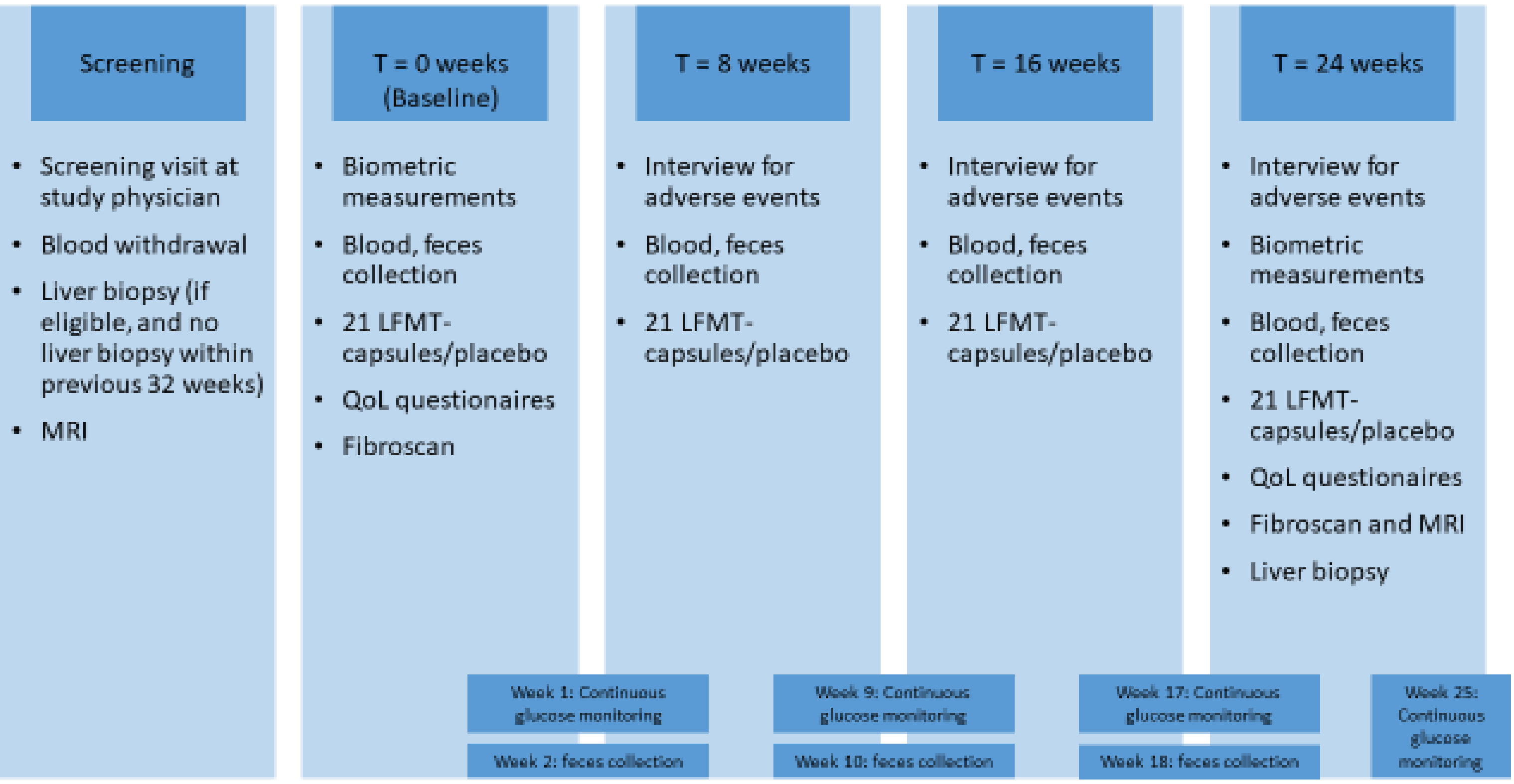

Figure 8: Overview of study visits

Appendix E: Insurance information

Amsterdam UMC has arranged insurance for everyone participating in the study. For everyone participating in this research, the AMC has taken out insurance. The insurance covers damage caused by participation in the study. This applies to damage during the study or within four years after the end of your participation in the study. You must have reported damage to the insurer within those four years.

The insurance does not cover all damages. The bottom of this text briefly states which damage is not covered.

These provisions can be found in the Decree on compulsory insurance in medical research involving human subjects. This decree can be found at www.ccmo.nl, the website of the Central Committee on Human Research (see 'Library' and then 'Laws and regulations').

In case of damage, please contact the insurer directly.

The insurer of the research is:
Name: Centramed B.A.
Address: P.O. Box 7374, 2701 AJ Zoetermeer
Telephone number: 070 301 70 70
E-mail: info@centramed.nl
Policy number: 624.528.303

The insurance provides coverage of € 650,000 per test subject with a maximum of € 5,000,000 for the entire study and € 7,500,000 for damages resulting from medical scientific research reported per insurance year.

The insurance does not cover the following damages:
- damage due to a risk about which you were informed in the written information. This does not apply if the risk occurs more seriously than was foreseen or if the risk was very unlikely;
- damage to your health that would have occurred even if you had not participated in the study;
- damage due to not (fully) following directions or instructions;
- damage to your offspring, as a result of a negative effect of the research on you or your offspring;
- damage as a result of research into existing treatment methods.

Furthermore, the test subject is requested to contact the principal investigator, Dr. A.G. Holleboom, tel. 020-5661925, in this regard.

Appendix F: Contact details Amsterdam UMC, location AMC.

Research physicians:

Executive Investigator
Drs. Q.J.J. Augustijn, MD
Department of vascular medicine
Amsterdam UMC, location AMC
Tel: 020 5661267 (available during office hours)
Email: q.j.augustijn@amsterdamumc.nl

Principal investigator
Dr. A.G. Holleboom, MD PhD
Department of vascular medicine
Amsterdam UMC, location AMC
Tel: 020 5661925
Email: a.g.holleboom@amsterdamumc.nl

Research nurse

D. Zwirs
Department of vascular medicine
Amsterdam UMC, location AMC
Tel: 020 5666638
Email: d.zwirs@amsterdamumc.nl

Independent physician

Dr. N.M.J. Hanssen, internist vascular medicine and endocrinologist.
Department of vascular medicine
Amsterdam UMC, location AMC.
Tel: 020 - 566 91 11, ask for Dr. Hanssen.
E-mail: n.m.j.hanssen@amsterdamumc.nl

Data protection officer

privacy@amsterdamumc.nl

Complaints

Patient Information & Complaints Department, telephone number: 020-5663355.Telephone access: during office hours.

Bijlage G: Informed consent form

Belonging to the SYNCH study.

- I read the information letter. I was also able to ask questions. My questions were answered well enough. I had enough time to decide whether to participate.
- I know that participating is voluntary. I also know that I can decide at any time not to participate in the study after all. Or to stop. I do not have to say why I want to stop.
- I give the researcher permission to inform my primary care physician who is treating me that I am participating in this study.
- I give the researcher permission to give my GP or specialist information about unexpected findings from the study that are important to my health.
- I give the researchers permission to collect and use my data and body material. The researchers do this only to answer the research question of this study.
- I know that to monitor the study, some people will be able to see all of my data. Those people are listed in this information letter. I give these people permission to see my data for this audit.
- I know that I cannot become pregnant during the study.
- The researcher has discussed with me how best to prevent me from becoming pregnant.
- Would you please check yes or no in the table below?

I give permission to keep my data to use this for other research, as stated in the information letter.	Yes ☐	No ☐
I give permission to keep my (remaining) body material to use it for other research, as stated in the information letter. The body material will be kept for 5 years for this purpose.	Yes ☐	No ☐
I give permission to ask me if necessary after this study if I want to participate in a follow-up study.	Yes ☐	No ☐
I give the researchers permission to let me know after the study what treatment I had/ in which group I was in.	Yes ☐	No ☐

- I want to participate in this study.

My name is (subject): ………………………………..

Signature: ……………………… Date: __ / __ / __

I certify that I have fully informed this subject about the said study.

Will any information become known during the research that may affect the subject's consent? If so, I will let this subject know in a timely manner.

Investigator's name (or representative):....................................

Signature:..........................   Date: __ / __ / __

Additional information was given by:
Name:.....................................
Position:...................................

Signature:..........................   Date:__ / __ / __

A complete information letter will be given to the subject along with a signed version of the consent form.
