# Supplementary material for: Randomised double-blind placebo-controlled trial protocol to evaluate the therapeutic efficacy of lyophilised faecal microbiota capsules amended with next-generation beneficial bacteria in individuals with metabolic dysfunction-associated steatohepatitis
Source: BMJ Open. 2025 Jan 9;15(1):e088290. doi: 10.1136/bmjopen-2024-088290 (PMC11784342; doi:10.1136/bmjopen-2024-088290)
Supplement: Supplementary file 6 [file bmjopen-2024-088290.reviewer_comments.pdf]

PEER REVIEW HISTORY

BMJ Open publishes all reviews undertaken for accepted manuscripts. Reviewers are asked to complete a checklist review form and are provided with free text boxes to elaborate on their assessment. These free text comments are reproduced below.

ARTICLE DETAILS

Title (Provisional)

Randomised double-blind placebo-controlled trial protocol to evaluate the therapeutic efficacy of lyophilised faecal microbiota capsules amended with next-generation beneficial bacteria in individuals with metabolic dysfunction-associated steatohepatitis

Authors

Augustijn, Quinten Jan Julian; Grefhorst, Aldo; de Groen, Pleun; Wortelboer, Koen; Seegers, Jos F.M.L.; Gül, Ismail Sahin; Suenaert, Peter; Verheij, Joanne; de Vos, Willem; Herrema, Hilde; Nieuwdorp, Max; Holleboom, Adriaan

---

VERSION 1 - REVIEW

Reviewer 1
Name Péterfi, Zoltán
Affiliation Division of Infectious Diseases, 1st Department of Medicine, Medical School, University of Pécs,, 1st Department of Internal Medicine
Date 11-Jun-2024
COI There are no competing interests.

We have read a well compiled and well thought out protocol.

The compilation of the protocol meets the prescribed criteria.

Some small improvements do not have a significant impact on ongoing patient enrolment.

- According to the checklist, it is necessary to indicate the version of the protocol.

Please replace this.

- In connection with donor screening, the Protocol recommends the donor age from 18 to 75 years among the donor selection criteria. In previous animal models, transplants from elderly donors increased the risk of colitis, colorectal cancer and possibly other metabolic diseases. Some fecal transplant protocols recommended setting the upper age to 45 years, which correlates with the criteria for most protocols. I suggest reducing the upper age to 45 years.

- In the references, the name of the microorganism should be in italics.

I support the protocol to be published.

**Reviewer**	**2**
**Name**	**Mullish, Benjamin**
**Affiliation**	**Imperial College London Faculty of Medicine, Surgery & Cancer**
**Date**	**24-Jul-2024**
**COI**	**I have no competing interests to declare**

This is overall clear and well-constructed, and most key information is included. However, I have a few particular comments:

-Introduction, line 28 - I am surprised that the only FMT study mentioned here is this group's previous MASLD, study, and not any mention of FMT/ metabolic syndrome studies (including their own extensive experience in this area), as well as the other (albeit small/ early) FMT/ MASLD studies, i.e. Craven study (https://pubmed.ncbi.nlm.nih.gov/32618656/), possibly the Frontiers study (https://www.frontiersin.org/journals/cellular-and-infection-microbiology/articles/10.3389/fcimb.2022.759306/full). A little critical analysis of the strengths/ weaknesses/ unknowns from this study might be of interest too.

-Re use of vegan donors - earlier trials using vegan donors by this group are quoted - but what is the evidence that vegan donors have more SCFA-producing capacity/ higher levels of SCFAs in biofluids vs carnivores?

-Study setting – this is a single centre study in Amsterdam – will you be taking measures to ensure equal male: female recruitment? How will you try and ensure recruitment from people from different ethnicities/ socio-cultural backgrounds?

-'Investigational products':

--One of the very unique aspects of this study is the use of at least some FMT capsules daily (with larger spaced loading/ top ups) - could you give some justification for this aspect of study design? What quality control do you have on capsules - do they have a particular CFU count of bacteria within them?

--If patients require antibiotics during the study – do they get any 'boost' in capsule number, or unchanged?

-Participant allocation and blinding – could you explain a bit more stratifying by metformin use? Does baseline HbA1c, diabetes status or lipid profile influence stratification/ expected outcomes?

-Discussion – there is no discussion, which is normally a useful resource for critical analysis of the previous work in this area/ discussion of strengths and weaknesses of this project/ horizon scanning for what might happen based on the results of this study – please consider this.

-PPIE – there is no described patient contribution to the study design, was this really the case? Why not if so?

-It would be interesting to consider adding the patient information sheet, consent form, and full protocol as supplements.

VERSION 1 - AUTHOR RESPONSE

Reviewer: 1
Dr. Zoltán Péterfi, Division of Infectious Diseases, 1st Department of Medicine, Medical School, University of Pécs,
Comments to the Author:
We have read a well compiled and well thought out protocol.
The compilation of the protocol meets the prescribed criteria.
Some small improvements do not have a significant impact on ongoing patient enrolment.

1.- According to the checklist, it is necessary to indicate the version of the protocol.
Please replace this.

Thank you for reviewing this paper and thank you for this comment. We agree and have added the version of the protocol (version 5; page 6, line 41, section 'Study design').

2. - In connection with donor screening, the Protocol recommends the donor age from 18 to 75 years among the donor selection criteria. In previous animal models, transplants from elderly donors increased the risk of colitis, colorectal cancer and possibly other metabolic diseases. Some fecal transplant protocols recommended setting the upper age to 45 years, which correlates with the criteria for most protocols. I suggest reducing the upper age to 45 years.

Many thanks for this suggestion. Although, this evidence is restricted to animal studies and no study in humans confirms this hypothesis, we agree with your consideration.
The choice to adopt 18-75 years as a donor selection criterium was made to expand the potential donor pool, and thereafter perform a case-by-case evaluation of the potential donor and possible risks. In our experience, recruiting donors that pass the stringent donor-screening, is difficult. However, it was our intention to indeed select donors below 45 years old. Luckily, this worked out; our oldest donor is 34 years old.
In retrospect, it would have been a good idea to change the age limit to 45 years. However, since we already have recruited the donors and used their faeces, we believe it would be not fair to change the criteria thereafter.

3. - In the references, the name of the microorganism should be in italics.

Thank you for this comment. We have adjusted it accordingly.

Reviewer: 2
Dr. Benjamin Mullish, Imperial College London Faculty of Medicine
Comments to the Author:
This is overall clear and well-constructed, and most key information is included.
However, I have a few particular comments:

1. -Introduction, line 28 - I am surprised that the only FMT study mentioned here is this group's previous MASLD, study, and not any mention of FMT/ metabolic syndrome studies (including their own extensive experience in this area), as well as the other (albeit small/ early) FMT/ MASLD studies, i.e. Craven study (https://pubmed.ncbi.nlm.nih.gov/32618656/), possibly the Frontiers study (https://www.frontiersin.org/journals/cellular-and-infection-microbiology/articles/10.3389/fcimb.2022.759306/full). A little critical analysis of the strengths/ weaknesses/ unknowns from this study might be of interest too.

Field Code Changed

Field Code Changed

Thank you for your thorough review and this valuable comment. We agree that in an introduction of a paper discussing the protocol for a trial investigating FMT in MASLD, we should briefly discuss previous trials that did so. We therefore have added (several) studies to the revised version of the manuscript (section 'Introduction', page 4, line 29 onwards):

Although several studies show beneficial effects of lean donor faecal microbiota transplantation (FMT) on cardiometabolic parameters in individuals with metabolic syndrome, to date only three studies have specifically investigated the therapeutic potential of FMT in individuals with MASLD.[1-3] Firstly, Xue and colleagues compared colonic administered FMT with oral probiotics in an non-randomised open-label trial with a one-month follow-up.[4] Although this study found a decrease in liver fat attenuation scores assessed by FibroScan after FMT treatment, the implications are difficult to interpret due to the study design, outcome measures, short follow-up and incomplete reporting of potential confounders. Secondly, an Canadian randomised placebo-controlled trial in 21 individuals comparing a single dose of allogenic with autologous FMT via nasoduodenal tube found an improvement in intestinal barrier function, but no improvement in liver fat content as assessed by MRI proton density fat fraction (MRI-PDFF).[5] However, this study is also difficult to interpret, mainly due to its single-dose nature, short six week follow-up and a small sample size. Thirdly, a randomised controlled trial from our group compared three infusions of allogenic *vs*. autologous FMT via nasoduodenal tube administered every 8 weeks.[6] Although, this study also had a small sample size, an allogenic FMT from lean donors on a vegan diet reduced the histopathological hepatic necroinflammation score in individuals with MASLD. Participants treated with the allogenic FMT displayed an increased relative abundance of butyrate-producing bacteria such as *Faecalibacterium prausnitzii* and *Anaerobutyricum soehngenii* (previously termed *Eubacterium hallii*[7]). Unfortunately, the latter study did not use validated imaging outcome measures to confirm histopathological scores and did not identify a clear causal mechanistic explanation.

2.-Re use of vegan donors - earlier trials using vegan donors by this group are quoted - but what is the evidence that vegan donors have more SCFA-producing capacity/ higher levels of SCFAs in biofluids vs carnivores?

Thank you for this question, which remains a grand topic of debate.
In general, the primary rationale -that was also used in the FMT-trial of Witjes and colleagues- is that MASLD is less prevalent in people on a vegan diet, and that the gut microbiome of vegans is relatively enriched with butyrate producers and has a low abundance of gram-negative lactate producers. In addition, people on a vegan diet also have more favourable cardiometabolic parameters. Moreover, vegans in general consume more dietary fiber than omnivores, which theoretically would lead to more SCFA-producing species in the gut. A recent study indeed found that people on a vegan diet have higher SCFAs concentrations in faeces than omnivores.[8]
Further, vegans appear to have a higher abundance of the Phylum *Bacteriodetes* (and especially *Prevotella*) compared to omnivores and an increased alpha diversity, which both are decreased in most of the studies that characterize the gut microbiota in MASLD patients.[9]

3.-Study setting – this is a single centre study in Amsterdam – will you be taking measures to ensure equal male: female recruitment? How will you try and ensure recruitment from people from different ethnicities/ socio-cultural backgrounds?

Thank you for this consideration. The Amsterdam UMC, location AMC, is located in the far south-East of Amsterdam. The population of this part of Amsterdam is highly diverse in socio-cultural backgrounds, as, for example, is displayed in several studies from the HELIUS-cohort that describe the same population (an example of an HELIUS-paper: Ethnic variations in metabolic syndrome components and their associations with the gut microbiota: the HELIUS study | Genome Medicine | Full Text (biomedcentral.com). In addition, part of the participants will be recruited via the MASLD outpatient clinic. This tertiary clinic partly comprises of patients that are referred from outside the region and therefore the geographical scope these patients originate from is wider than merely the surrounding of the AMC. Lastly, we spread the word on this trial through national meetings of gastroenterologists, endocrinologist and vascular medicine specialists.
Nevertheless, the reviewer is right that the single-centre set-up might be a limitation of our study, hence we added this fact to the list of limitations in the revised version of our manuscript (page 3).

Field Code Changed

No measures are taken to ensure equal male-to-female recruitment ratios. This is due to two factors. Firstly, it is our objective to include a population that is as similar as possible to that of the outpatient MASH clinic. Secondly, given the already challenging and prolonged process of inclusion for a double-biopsy FMT study (Witjes et al. terminated prematurely due to the slow inclusion rate of 21 participants over four years), it is not feasible to implement a selective recruitment process while maintaining a reasonable inclusion rate.

4. -'Investigational products':
a. --One of the very unique aspects of this study is the use of at least some FMT capsules daily (with larger spaced loading/ top ups) - could you give some justification for this aspect of study design? What quality control do you have on capsules - do they have a particular CFU count of bacteria within them?

Thank you for this question. As you will agree, one of the main advantages of the capsules is that they can be administered daily. Although not evidence-based, the rationale for the scheme of 3 times 21 lyophilised FMT-capsules (LFMCs) at baseline, week 8 and 16, is to mimic the intermittent nature of the trial of Witjes et al. 2019.[6] In addition, participants ingest 2 LFMCs daily to maintain the hypothesised effect. We added this to counter the variability due to unpredictable differences in engraftment and to prevent the gut microbiome composition to shift back towards the original gut microbiome under the influence of the diet (participants are instructed to not alter dietary

habit) and other host-factors. The total mass of faeces administered in our study equals approximately 3 times 200 grams of faeces, thereby again mimicking the study of Witjes et al.

Our study pharmacist Dr. Koen Wortelboer and coauthor on the study has done extensive research on the CFU's, viability and storage of the LFMCs. He and colleagues assessed the CFU after preparation and after storage among different circumstances and durations for both aerobic and anaerobic bacteria. The results of the LFMC stability studies will be send out for publication in the near future.

b. --If patients require antibiotics during the study – do they get any 'boost' in capsule number, or unchanged?

Thank you for this question. When a participant requires antibiotics, we will bring the next study visit with the ingestion of 21 capsules forward to be soon after the antibiotics. This aspect was indeed missing in the first version of our manuscript and has now been added in the section 'investigational products' (page 8, line 42 onwards).

c. -Participant allocation and blinding – could you explain a bit more stratifying by metformin use? Does baseline HbA1c, diabetes status or lipid profile influence stratification/ expected outcomes?

We chose metformin as a stratification criterium because of its known influences on the gut microbiota composition. (e.g. Metformin alters the gut microbiome of individuals with treatment-naive type 2 diabetes, contributing to the therapeutic effects of the drug | Nature Medicine; Effects of metformin on the gut microbiota: A systematic review - PubMed (nih.gov ; Disentangling type 2 diabetes and metformin treatment signatures in the human gut microbiota | Nature)).[10-12] However, we can imagine that a high HbA1c can imply increased metabolic derangement and a more severe pathophysiological driver of MASH, with subsequent effects on outcome.

Field Code Changed

Field Code Changed

Field Code Changed

d. -Discussion – there is no discussion, which is normally a useful resource for critical analysis of the previous work in this area/ discussion of strengths and weaknesses of this project/ horizon scanning for what might happen based on the results of this study – please consider this.

Thank you for this suggestion. In the submission guidelines and the articles that we scanned to investigate the style of BMJ protocols, we did not find a discussion. However, we certainly do agree that a discussion is useful and have added a concise discussion (page 14 and further).

For your convenience, below a copy of the included discussion:

Discussion

The objective of this study is to investigate the potential of continuous FMT treatment via capsules supplemented with next-generation beneficial bacteria as a viable treatment for MASH. We hypothesise that FMT and next-generation beneficial bacteria might not only complement each other, but also work synergistically to a surplus effect. The next-generation beneficial bacteria used in this study are less prevalent in patients with MASH. Thus, it is to be expected that these bacteria thrive less in the gut of individuals with MASLD and subsequently have less potential in exerting their beneficial effects when given as solo treatment.[6,13,14] The introduction of a richer microbial community through FMT creates a more favourable environment for next-generation beneficial

bacteria, which could theoretically make these bacteria thrive and engraft more effectively. This could enhance their efficacy, as they benefit from a bacterial network in which they naturally occur in greater abundance. *Vice versa*, the next-generation beneficial bacteria can stimulate the symbiotic bacteria present in the FMT. We therefore postulate that the combination of FMT and next-generation beneficial bacteria has the potential to lead to a more significant improvement in the pathophysiology of MASH than when applied separately.

Many studies suggest that the gut microbiome plays a pivotal role in the multifaceted pathophysiology of MASLD. To date, several studies have investigated the potential of pre- and probiotics as a treatment for MASLD. While some studies have reported positive outcomes with different formulations of pre-, pro-, and synbiotics, these studies unfortunately fall short to accurately assess the potential of gut microbiome modulation in the treatment of MASH in humans.[15,16] Firstly, the majority of controlled trials that report a reduction in MASLD following conventional probiotic therapy in humans utilise surrogate endpoints, such as anthropometric parameters, blood tests or controlled attenuation parameter by FibroScan. Secondly, the majority of studies conducted in humans have only employed the use of *Lactobacillus* or *Bifidobacterium spp.*, or a combination thereof, that are not found in large numbers in the human gut. Of note, certain *Lactobacillus* species of can worsen MASLD by the production of ethanol.[17] New next-generation beneficial bacteria that derive from the human intestinal tract are increasingly identified as having beneficial properties on the metabolic system.[18,19] However, despite the growing body of evidence on the potential of next-generation beneficial bacteria in the treatment of a plethora of metabolic diseases in humans and MASLD in mice, studies utilising next-generation beneficial bacteria in humans with MASLD have yet to be performed. Thirdly, the sample sizes of all probiotic studies are relatively small, possibly attributable to their investigator-initiated nature.

Although several studies indicate beneficial effects of lean donor FMT on cardiometabolic parameters in metabolic syndrome, only three have specifically examined its therapeutic potential in MASLD.[4-6] Xue *et al.* demonstrated a moderate reduction in liver fat attenuation scores assessed by FibroScan post-FMT.[4] This effect was more pronounced in lean MASH patients, supporting the hypothesis that gut microbiota are causally involved in MASH development independent of caloric overload. However, it should be noted that a fat attenuation score is a variable surrogate marker of hepatic steatosis and therefore requires caution in interpretation, especially in small sample sizes. Moreover, the absence of true randomization, short one-month follow-up, and incomplete reporting confounding factors limit interpretability of this trial. The second study, a placebo-controlled trial from Craven *et al.*, did not show changes in liver fat content after FMT, as assessed by MRI-PDFF.[5] However, it remains debatable if a single FMT of 2 grams has the potential to significantly improve MASLD within a six-week follow-up, especially when studied in only 15 subjects.[20] Nevertheless, the study did show improvements in gut barrier function after allogenic FMT, which is postulated to play a key role in the gut-liver axis and the development of MASLD.[21] Lastly, Witjes *et al.* compared three infusions via a nasoduodenal tube of allogenic versus autologous FMT every eight weeks. Allogenic FMT resulted in a reduction in histopathological hepatic necroinflammation and an increase in the faecal abundance of the butyrate-producers *F. prausnitzii* and *A. soehngenii*. However, the modest sample size and the broad inclusion criteria (obese patients with simple steatosis sufficed) are significant limitations in the latter study.

The current study is robust due to its randomised, double-blind design and the use of liver biopsy as a primary outcome measure, complemented by non-invasive tests such as MRI-PDFF and MRE to mitigate sample error. The use of capsules to administer FMT has been successfully applied in *Clostridium difficile* infections (CDI), yet its application in the context of metabolic diseases remains less explored.[22] However, there are multiple advantages associated with the use of capsules in comparison to the conventional FMT method via nasoduodenal tube. Firstly, capsules are more patient-friendly and safe.[23,24] Secondly, faecal microbiota transplantation via duodenal tube is administered intermittently, allowing the microbiome to revert to its original state between

treatments, which may scrutinize treatment effects. Continuous delivery of FMT via capsules could enhance therapeutic efficacy. Finally, capsules are closest to a regular treatment and therefore closest to translation into therapy.
The duration of 24-week might be a limitation as it raises questions about its sufficiency for demonstrating effects, particularly on liver fibrosis; however, previous pharmacological studies have shown efficacy within similar timeframes.[25,26] Another limitation is the absence of a control group or pure placebo group, which hampers the ability to draw definitive conclusions about the treatment's efficacy. This can be partly overcome by comparing to placebo arm biopsies from previous clinical trials with a comparable study population and duration.
To the best of our knowledge, this is the first study to combining FMT with next-generation beneficial bacteria to treat any metabolic disease. The study will help answer the question if gut microbiome modulation can be a feasible treatment for MASH. It will also test the novel hypothesis of providing a beneficial environment for beneficial bacteria utilizing FMT. Modulation of the gut microbiome is a safe and potentially inexpensive treatment with mild and few side effects. Further understanding of its applicability in common metabolic disease may be of great value.

e. -PPIE – there is no described patient contribution to the study design, was this really the case? Why not if so?

Thank you for this question. We indeed did not include a patient in the design of the study. The main reason for this is that we think this is a straightforward and lean trial. The main burden for the participants, namely the double liver biopsy, is a necessity in this trial. Further, many alterations were not possible within the means that we possess to complete this trial. We expected that a review by a patient would therefore not alter the design of the study. Lastly, this trial offers treatment in both arms and the treatment has little side effects and risks.
There is a patient in the Medical Ethics Committee of the Amsterdam UMC that reviewed the study. This patient specifically evaluates the study on the burden and point of view of the participants.

f. -It would be interesting to consider adding the patient information sheet, consent form, and full protocol as supplements.

Thank you for this suggestion. We have added the documents as supplement. The informed consent form is at the end of the patient information letter.

References

1. Vrieze A, Van Nood E, Holleman F, Salojarvi J, Kootte RS, Bartelsman JF, Dallinga-Thie GM, Ackermans MT, Serlie MJ, Oozeer R, et al. Transfer of intestinal microbiota from lean donors increases insulin sensitivity in individuals with metabolic syndrome. *Gastroenterology*. 2012;143:913-916 e917. doi: 10.1053/j.gastro.2012.06.031
2. Kootte RS, Levin E, Salojarvi J, Smits LP, Hartstra AV, Udayappan SD, Hermes G, Bouter KE, Koopen AM, Holst JJ, et al. Improvement of Insulin Sensitivity after Lean Donor Feces in Metabolic Syndrome Is Driven by Baseline Intestinal Microbiota Composition. *Cell Metab*. 2017;26:611-619 e616. doi: 10.1016/j.cmet.2017.09.008
3. Mocanu V, Zhang Z, Deehan EC, Kao DH, Hotte N, Karmali S, Birch DW, Samarasinghe KK, Walter J, Madsen KL. Fecal microbial transplantation and fiber supplementation in patients with severe obesity and metabolic syndrome: a randomized double-blind, placebo-controlled phase 2 trial. *Nat Med*. 2021;27:1272-1279. doi: 10.1038/s41591-021-01399-2

4. Xue L, Deng Z, Luo W, He X, Chen Y. Effect of Fecal Microbiota Transplantation on Non-Alcoholic Fatty Liver Disease: A Randomized Clinical Trial. *Front Cell Infect Microbiol*. 2022;12:759306. doi: 10.3389/fcimb.2022.759306
5. Craven L, Rahman A, Nair Parvathy S, Beaton M, Silverman J, Qumosani K, Hramiak I, Hegele R, Joy T, Meddings J, et al. Allogenic Fecal Microbiota Transplantation in Patients With Nonalcoholic Fatty Liver Disease Improves Abnormal Small Intestinal Permeability: A Randomized Control Trial. *Am J Gastroenterol*. 2020;115:1055-1065. doi: 10.14309/ajg.0000000000000661
6. Witjes JJ, Smits LP, Pekmez CT, Prodan A, Meijnikman AS, Troelstra MA, Bouter KEC, Herrema H, Levin E, Holleboom AG, et al. Donor Fecal Microbiota Transplantation Alters Gut Microbiota and Metabolites in Obese Individuals With Steatohepatitis. *Hepatol Commun*. 2020;4:1578-1590. doi: 10.1002/hep4.1601
7. Shetty SA, Zuffa S, Bui TPN, Aalvink S, Smidt H, De Vos WM. Reclassification of Eubacterium hallii as Anaerobutyricum hallii gen. nov., comb. nov., and description of Anaerobutyricum soehngenii sp. nov., a butyrate and propionate-producing bacterium from infant faeces. *Int J Syst Evol Microbiol*. 2018;68:3741-3746. doi: 10.1099/ijsem.0.003041
8. Losno EA, Sieferle K, Perez-Cueto FJA, Ritz C. Vegan Diet and the Gut Microbiota Composition in Healthy Adults. *Nutrients*. 2021;13. doi: 10.3390/nu13072402
9. Prochazkova M, Budinska E, Kuzma M, Pelantova H, Hradecky J, Heczkova M, Daskova N, Bratova M, Modos I, Videnska P, et al. Vegan Diet Is Associated With Favorable Effects on the Metabolic Performance of Intestinal Microbiota: A Cross-Sectional Multi-Omics Study. *Front Nutr*. 2021;8:783302. doi: 10.3389/fnut.2021.783302
10. Wu H, Esteve E, Tremaroli V, Khan MT, Caesar R, Manneras-Holm L, Stahlman M, Olsson LM, Serino M, Planas-Felix M, et al. Metformin alters the gut microbiome of individuals with treatment-naive type 2 diabetes, contributing to the therapeutic effects of the drug. *Nat Med*. 2017;23:850-858. doi: 10.1038/nm.4345
11. Forslund K, Hildebrand F, Nielsen T, Falony G, Le Chatelier E, Sunagawa S, Prifti E, Vieira-Silva S, Gudmundsdottir V, Pedersen HK, et al. Disentangling type 2 diabetes and metformin treatment signatures in the human gut microbiota. *Nature*. 2015;528:262-266. doi: 10.1038/nature15766
12. Pavlo P, Kamyshna I, Kamyshnyi A. Effects of metformin on the gut microbiota: A systematic review. *Mol Metab*. 2023;77:101805. doi: 10.1016/j.molmet.2023.101805
13. Koning M, Herrema H, Nieuwdorp M, Meijnikman AS. Targeting nonalcoholic fatty liver disease via gut microbiome-centered therapies. *Gut Microbes*. 2023;15:2226922. doi: 10.1080/19490976.2023.2226922
14. Aron-Wisnewsky J, Vigliotti C, Witjes J, Le P, Holleboom AG, Verheij J, Nieuwdorp M, Clement K. Gut microbiota and human NAFLD: disentangling microbial signatures from metabolic disorders. *Nat Rev Gastroenterol Hepatol*. 2020;17:279-297. doi: 10.1038/s41575-020-0269-9
15. Xing W, Gao W, Lv X, Zhao Z, Mao G, Dong X, Zhang Z. The effects of supplementation of probiotics, prebiotics, or synbiotics on patients with non-alcoholic fatty liver disease: A meta-analysis of randomized controlled trials. *Front Nutr*. 2022;9:1024678. doi: 10.3389/fnut.2022.1024678
16. Carpi RZ, Barbalho SM, Sloan KP, Laurindo LF, Gonzaga HF, Grippa PC, Zutin TLM, Girio RJS, Repetti CSF, Detregiachi CRP, et al. The Effects of Probiotics, Prebiotics and Synbiotics in Non-Alcoholic Fat Liver Disease (NAFLD) and Non-Alcoholic Steatohepatitis (NASH): A Systematic Review. *Int J Mol Sci*. 2022;23. doi: 10.3390/ijms23158805
17. Meijnikman AS, Davids M, Herrema H, Aydin O, Tremaroli V, Rios-Morales M, Levels H, Bruin S, de Brauw M, Verheij J, et al. Microbiome-derived ethanol in nonalcoholic fatty liver disease. *Nat Med*. 2022;28:2100-2106. doi: 10.1038/s41591-022-02016-6
18. Cani PD, Depommier C, Derrien M, Everard A, de Vos WM. Akkermansia muciniphila: paradigm for next-generation beneficial microorganisms. *Nat Rev Gastroenterol Hepatol*. 2022. doi: 10.1038/s41575-022-00631-9
19. Vallianou NG, Kounatidis D, Tsilingiris D, Panagopoulos F, Christodoulatos GS, Evangelopoulos A, Karampela I, Dalamaga M. The Role of Next-Generation Probiotics in Obesity and Obesity-Associated Disorders: Current Knowledge and Future Perspectives. *Int J Mol Sci*. 2023;24. doi: 10.3390/ijms24076755
20. Ianiro G, Porcari S, Gasbarrini A, Cammarota G. Quantity of Donor Stool for Fecal Microbiota Transplantation: The More, the Better? *Am J Gastroenterol*. 2021;116:1360-1361. doi: 10.14309/ajg.0000000000001130

21. Luther J, Garber JJ, Khalili H, Dave M, Bale SS, Jindal R, Motola DL, Luther S, Bohr S, Jeoung SW, et al. Hepatic Injury in Nonalcoholic Steatohepatitis Contributes to Altered Intestinal Permeability. *Cell Mol Gastroenterol Hepatol*. 2015;1:222-232. doi: 10.1016/j.jcmgh.2015.01.001
22. Halaweish HF, Boatman S, Staley C. Encapsulated Fecal Microbiota Transplantation: Development, Efficacy, and Clinical Application. *Front Cell Infect Microbiol*. 2022;12:826114. doi: 10.3389/fcimb.2022.826114
23. Staley C, Hamilton MJ, Vaughn BP, Graiziger CT, Newman KM, Kabage AJ, Sadowsky MJ, Khoruts A. Successful Resolution of Recurrent Clostridium difficile Infection using Freeze-Dried, Encapsulated Fecal Microbiota; Pragmatic Cohort Study. *Am J Gastroenterol*. 2017;112:940-947. doi: 10.1038/ajg.2017.6
24. Jiang ZD, Jenq RR, Ajami NJ, Petrosino JF, Alexander AA, Ke S, Iqbal T, DuPont AW, Muldrew K, Shi Y, et al. Safety and preliminary efficacy of orally administered lyophilized fecal microbiota product compared with frozen product given by enema for recurrent Clostridium difficile infection: A randomized clinical trial. *PLoS One*. 2018;13:e0205064. doi: 10.1371/journal.pone.0205064
25. Francque SM, Bedossa P, Ratziu V, Anstee QM, Bugianesi E, Sanyal AJ, Loomba R, Harrison SA, Balabanska R, Mateva L, et al. A Randomized, Controlled Trial of the Pan-PPAR Agonist Lanifibranor in NASH. *N Engl J Med*. 2021;385:1547-1558. doi: 10.1056/NEJMoa2036205
26. Harrison SA, Bashir MR, Guy CD, Zhou R, Moylan CA, Frias JP, Alkhouri N, Bansal MB, Baum S, Neuschwander-Tetri BA, et al. Resmetirom (MGL-3196) for the treatment of non-alcoholic steatohepatitis: a multicentre, randomised, double-blind, placebo-controlled, phase 2 trial. *Lancet*. 2019;394:2012-2024. doi: 10.1016/S0140-6736(19)32517-6

VERSION 2 - REVIEW

**Reviewer**	**2**
**Name**	**Mullish, Benjamin**
**Affiliation**	**Imperial College London Faculty of Medicine, Surgery & Cancer**
**Date**	**03-Dec-2024**
**COI**	

This is a very clear protocol, and I am entirely satisfied with the responses to my reviewer comments. The responses to the editorial comments and those of the other reviewer also look satisfactory to me. Thank you.
